# Detecting steps in spatial genetic data: Which diversity measures are best?

**DOI:** 10.1371/journal.pone.0265110

**Published:** 2022-03-14

**Authors:** Alexander T. Sentinella, Angela T. Moles, Jason G. Bragg, Maurizio Rossetto, William B. Sherwin

**Affiliations:** 1 Evolution & Ecology Research Centre, School of Biological, Earth and Environmental Sciences, UNSW Sydney, Sydney, NSW, Australia; 2 Research Centre for Ecosystem Resilience, Australian Institute of Botanical Science, The Royal Botanic Garden Sydney, Sydney, NSW, Australia; Universitat Pompeu Fabra, SPAIN

## Abstract

Accurately detecting sudden changes, or steps, in genetic diversity across landscapes is important for locating barriers to gene flow, identifying selectively important loci, and defining management units. However, there are many metrics that researchers could use to detect steps and little information on which might be the most robust. Our study aimed to determine the best measure/s for genetic step detection along linear gradients using biallelic single nucleotide polymorphism (SNP) data. We tested the ability to differentiate between linear and step-like gradients in genetic diversity, using a range of diversity measures derived from the *q*-profile, including allelic richness, Shannon Information, *G*_*ST*_, and *Jost-D*, as well as Bray-Curtis dissimilarity. To determine the properties of each measure, we repeated simulations of different intensities of step and allele proportion ranges, with varying genome sample size, number of loci, and number of localities. We found that alpha diversity (within-locality) based measures were ineffective at detecting steps. Further, allelic richness-based beta (between-locality) measures (e.g., Jaccard and Sørensen dissimilarity) were not reliable for detecting steps, but instead detected departures from fixation. The beta diversity measures best able to detect steps were: Shannon Information based measures, *G*_*ST*_ based measures, a *Jost-D* related measure, and Bray-Curtis dissimilarity. No one measure was best overall, with a trade-off between those measures with high step detection sensitivity (*G*_*ST*_ and Bray-Curtis) and those that minimised false positives (a variant of Shannon Information). Therefore, when detecting steps, we recommend understanding the differences between measures and using a combination of approaches.

## Introduction

Identifying a sudden change in genetic diversity (here referred to as a ‘step’) across a landscape is essential for many fields. A landscape geneticist may want to know if a barrier to gene flow is leading to geographical population structure [1]. An evolutionary ecologist may be examining how the frequencies of selectively important loci change across an environment [2]. Or a conservation manager may need to determine whether to treat a seemingly homogenous population as different management units [3]. By having the most effective set of measures to detect a step, scientists can make better inferences about their study system. However, there are a wide range of different metrics available, and little information about which are most effective at detecting changes in genetic diversity [4,5]. Here, we aim to bridge that gap by using simulated data to present a guide to molecular ecologists on the best way to detect steps in genetic data using a spectrum of diversity measures.

In molecular ecology, the identification of a step along a gradient of genetic diversity requires us to detect a significant change in alpha diversity (the genetic diversity of each sampled location) and/or beta diversity (pairwise genetic differentiation/distance between locations). Because beta diversity is, by definition, measuring differences between localities, it is seen to be more effective for detecting steps [5]. However, because alpha diversity does not change linearly with allele proportion changes, it can also be used in step detection in concert with beta diversity (e.g. [6]). Choosing a diversity measure that can best detect a step is difficult because there are many proposed measures for alpha and beta diversity, and there is much debate on which should be used in various situations [4].

Even with the wide variety of techniques and software available to investigate genetic steps [7], there is a surprising lack of variety in the genetic diversity measures used within them. Many of the most commonly used programs (such as GENELAND [8]; Barrier [9]) or techniques [10] measure genetic distance with *F*_*ST*_, *G*_*ST*_ and similar measures (also known as *q* = 2 based measures, see below). Other programs (such as dartR [11]; GENODIVE [12]) allow for the selection of a wider variety of measures, but the defaults are set to the commonly used *F*_*ST*_/*G*_*ST*_ measures. Lastly, some methods do not use genetic diversity measures in the classical sense at all, instead using a diffusion approximation model [13], estimated migration surfaces [14,15], or more cluster-based metrics (e.g. STRUCTURE [16]; fastSTRUCTURE [17]). However, because some of these methods rely upon detecting departure from Hardy-Weinberg equilibrium they can also be seen as *q* = 2 (see below) related measures. These *F*_*ST*_/*G*_*ST*_/*q* = 2 related metrics are used as the default despite their much-discussed limitations (see below) and the existence of more robust genetic diversity metrics [18]. The lack of uptake of different diversity measures stems partly from the lack of theoretical literature demonstrating the comparative benefits of different measures [18], but more likely a lack of more practical demonstration how they can be applied to current research. By testing multiple diversity measures here, we hope to identify the most effective metrics to detect steps, which could then be added into the above analysis tools to improve their accuracy and effectiveness.

We arrange the different metrics considered according to the ***q*-profile** of diversity measurement–a convenient framework of diversity measurement that unites many of the most commonly used measures [18]. The *q* denotes a variable whose choice determines the “order” of the measure. This profile can be split into three informative values when applied to genetic data: richness of alleles or haplotypes **(*q* = 0)**, Shannon Information **(*q* = 1)**, and heterozygosity or nucleotide diversity **(*q* = 2)** [18]. Each value has an entropy and complexity level (“*H*-measure”) and a numbers equivalent diversity metric (“*D*-measure”). While *H*-measures and *D*-measures have similar properties for the same value of *q*, *D*-measures have the benefit of being on the same scale (“effective numbers”) for all values of *q*, allowing better comparison of diversity across values of *q*, studies and systems. Each level of *q*-profile has different properties, strengths and weaknesses (outlined below), and therefore it has been recommended that all three are used in concert [18].

The ***q* = 0** metrics measure the number of allelic types in a population and do not consider the frequencies of the alleles. The alpha diversity metric, allelic richness, is based on the total number of alleles in a population, and when comparing populations (beta diversity), *q* = 0 represents the number of unshared alleles between populations (variants include Jaccard dissimilarity and Sørensen dissimilarity [18]). Allelic richness measures (*q* = 0) weight rare alleles equally to common alleles, which has benefits and drawbacks. By weighting rare alleles higher, *q* = 0 based measures are better at detecting standing variation within samples [18]. However, this can only occur if all alleles are sampled, and sampled correctly. Alleles are easily missed with incomplete sampling, and *q* = 0 measures are prone to being biased by genotyping errors (which are especially common in biallelic data [19]). This problem with *q* = 0 measures cannot be completely eliminated even by the most recent correction for sampling bias [20].

The ***q* = 1** based measures are based on Shannon Information theory and weight each allele by its proportional abundance. The *q* = 1 measure for alpha diversity is based on the chance that a newly sampled allele will be of a novel type, whereas for beta diversity the mutual information (MI) is the chance that knowing an individual’s allelic type will correctly identify its location of origin (also transformed to Shannon Differentiation). Because measures with *q* > 0 include information on the proportional abundance of alleles, *q* = 1 can give greater insight than richness alone. Despite *q* = 1 based measures being frequently used in studies examining species diversity, for instance Shannon diversity index, they are currently used less in molecular diversity studies [18]. By considering rare alleles proportional to their abundance in the population, the *q* = 1 measures have fewer sampling error problems than *q* = 0 measures, and these problems can be eliminated by suitable corrections [20]. The *q* = 1 measures also have an advantage over *q* = 2 measures because the latter are insensitive to rare alleles (see below [18]).

The ***q* = 2** measures are the most commonly used measures of alpha and beta genetic diversity. They reflect the chance that two randomly selected alleles from a population are the same type. Familiar *q* = 2 measures include heterozygosity (*H*_*e*_) for alpha diversity, and *F*_*ST*_, *G*_*ST*_, Jost-D for beta diversity (genetic differentiation [21]). The *q* = 2 measures intrinsically down-weight rare alleles, which simultaneously makes them robust to sampling problems, but worse at detecting the presence of variation among rare alleles, which might be disadvantageous, because loci where the minor allele is rare are typically very common [18]. Some *q* = 2 measures also have some undesirable mathematical properties which may confuse interpretation of studies (e.g. beta diversity having a dependence on alpha diversity [22]).

While most genetic diversity measures are captured directly in the *q*-profile, some are not, but are still seen to have desirable properties, especially when using biallelic data. Specifically, **Bray-Curtis (BC)** dissimilarity, also known as allele frequency difference (AFD [23]), is a beta-diversity based measure that is simply the average absolute allele proportion difference between two sites [23]. It has been tangentially linked to the members of the *q*-profile [24], but has only recently been explored as a genetic diversity measure in direct comparison to other metrics in the *q*-profile. We test it here because it has been proposed as a straightforward measure of genetic dissimilarity when using biallelic data [23].

In this paper, we first ask which measures are sensitive to steps under standard conditions, here defined as a steep step with a maximal allele proportion difference, and a large genome sample size (*n*), number of loci (*L*), and localities sampled (*K*). Even under these conditions, we expect some measures to have low step sensitivity, through being unable to detect steps (false negatives) or having high rates of false positives. So, we next examine the measures with reasonable properties under standard conditions and ask which are more sensitive under suboptimal conditions. This allows us to differentiate step detection sensitivities between measures. Specifically, we ask how step sensitivity is affected by step steepness, the magnitude of allele proportion difference, proximity to fixation, genome sample size, number of loci, and number of localities. Lastly, we ask if there is a single measure (or set of measures) that has the best step detection over all conditions (i.e., has the lowest rates of both false negatives and false positives). All analyses are confined to biallelic loci, such as the currently popular single nucleotide polymorphisms (SNPs).

## Materials and methods

### Calculation of diversity measures

We calculated alpha and beta diversity measures including three levels of the *q*-profile (0, 1 and 2) using formulae adapted from Sherwin et. al. [18] for biallelic loci, as well as the beta diversity measure Bray-Curtis [23] (see Table 1 at the end of the methods for a full list of all terms and variables with reference to all equations in the text). To calculate the entropy measures (*H* measures) of alpha diversity we took the allele proportion of the minor allele at a locus (*p*) and/or the number of different alleles at a locus (*S*) and entered them into Eqs 1–3. The superscripts before *H* or *D* denote the order of *q*, while the subscripts denote the diversity measure type (alpha, beta or gamma).


$${}^{0}H_{\alpha }=S-1$$ (1)

$${}^{1}H_{\alpha }=-p*log_{e}\left(p\right)-\left(1-p\right)*log_{e}\left(1-p\right)$$ (2)

$${}^{2}H_{\alpha }=1-(p^{2}+\left(1-p{)}^{2}\right)$$ (3)

**Table 1:** Summary of variables and symbols used in text.

Variable	Definition
*AvFirst*, *AvLast*	Variants of beta diversity measures based on whether values are averaged across loci before or after calculation of measure.
*BC*	Bray-Curtis. The average absolute value of differences in allele proportions across loci. Eq 27
*d*	Distance along landscape, where *d* = 0 denotes the start of the range and *d* = 1 the end.
^0^ *D*_*α*_	“*D*” measure (effective number) of alpha diversity of order 0. Eq 4.
^1^ *D*_*α*_	“*D*” measure (effective number) of alpha diversity of order 1. Eq 5.
^2^ *D*_*α*_	“*D*” measure (effective number) of alpha diversity of order 2. Eq 6.
$\bar{{}^{q}D_{\alpha }}$	Average *“D*” measure (effective number) of alpha diversities between two localities of order *q*. Eq 21. Used to calculate “D” measures of beta diversity.
^*q*^ *D*_*γ*_	*“D*” measure (effective number) of gamma diversity of the pooled localities of order *q*. Eq 22. Used to calculate “D” measures of beta diversity.
^*q*^ *D*_*β*.*A*.*AvLast*_	*AvLast* variant *“D*” measure (effective number) of beta diversity of order *q*, using method A. Eq 23.
^*q*^ *D*_*β*.*A*.*AvFirst*_	*AvFirst* variant *“D*” measure (effective number) of beta diversity of order *q*, using method A. Eq 24.
^*q*^ *D*_*β*.*B*.*AvLast*_	*AvLast* variant *“D*” measure (effective number) of beta diversity of order *q*, using method B. Eq 25.
^*q*^ *D*_*β*.*B*.*AvFirst*_	*AvFirst* variant *“D*” measure (effective number) of beta diversity of order *q*, using method B. Eq 26.
*H*. *to*. *D*	One of the three Eqs (4–6) that converts “H” measures to “D” measures.
^0^ *H*_*α*_	“*H*” measure (entropy) of alpha diversity of order 0. Eq 1.
^1^ *H*_*α*_	“*H*” measure (entropy) of alpha diversity of order 1. Eq 2.
^2^ *H*_*α*_	“*H*” measure (entropy) of alpha diversity of order 2. Eq 3.
$\bar{{}^{q}H_{\alpha }}$	Average *“H*” measure (effective number) of alpha diversities between two localities of order *q*. Eq 7. Used to calculate “*H*” measures of beta diversity.
^*q*^ *H*_*γ*_	*“H*” measure (effective number) of gamma diversity of both localities of order *q*. Eq 8. Used to calculate “*H*” measures of beta diversity.
^0^ *H*_*β*.*Jac*.*AvLast*_	Jaccard dissimilarity, *AvLast* variant. “*H*” measure (entropy) of beta diversity of order 0. Eq 9.
^0^ *H*_*β*.*Jac*.*AvFirst*_	Jaccard dissimilarity, *AvFirst* variant. “*H*” measure (entropy) of beta diversity of order 0. Eq 10.
^0^ *H*_*β*.*Sor*.*AvLast*_	Sorenson dissimilarity, *AvLast* variant. “*H*” measure (entropy) of beta diversity of order 0. Eq 11.
^0^ *H*_*β*.*Sor*.*AvFirst*_	Sorenson dissimilarity, *AvFirst* variant. “*H*” measure (entropy) of beta diversity of order 0. Eq 12.
^1^ *H*_*β*.*MI*.*AvLast*_	Mutual Information, *AvLast* variant. “*H*” measure (entropy) of beta diversity of order 1. Eq 13.
^1^ *H*_*β*.*MI*.*AvFirst*_	Mutual Information, *AvFirst* variant. “*H*” measure (entropy) of beta diversity of order 1. Eq 14.
^1^ *H*_*β*.*ShD*.*AvLast*_	Shannon Differentiation, *AvLast* variant. “*H*” measure (entropy) of beta diversity of order 1. Eq 15.
^1^ *H*_*β*.*ShD*.*AvFirst*_	Shannon Differentiation, *AvFirst* variant. “*H*” measure (entropy) of beta diversity of order 1. Eq 16.
^2^ *H*_*β*.*GST*.*AvLast*_	*G*_*ST*_, *AvLast* variant. “*H*” measure (entropy) of beta diversity of order 2. Eq 17.
^2^ *H*_*β*.*GST*.*AvFirst*_	*G*_*ST*_, *AvFirst* variant. “*H*” measure (entropy) of beta diversity of order 2. Eq 18.
^2^ *H*_*β*.*JOST*.*AvLast*_	*Jost-D*, *AvLast* variant. “*H*” measure (entropy) of beta diversity of order 2. Eq 19.
^2^ *H*_*β*.*JOST*.*AvFirst*_	*Jost-D*, *AvFirst* variant. “*H*” measure (entropy) of beta diversity of order 2. Eq 20.
*K*	Number of localities sampled along landscape including *d* = 0 to *d* = 1
*L*	Total number of loci
*n*	Number of individual genomes sampled from each locality used to calculate measured allele proportion
*p*_1_, *p*_2_, *p*_*av*_	The minor allele proportion at a biallelic locus (alternate allele is: 1 –p_*_) in locality 1, locality 2, and in the pooled localities 1 and 2 *p*_*av*_ = (*p*_1_ + *p*_2_)/2pav
*p*_*start*_, *p*_*end*_	Allele proportion at *d* = 0 and *d* = 1 respectively
*q*	“Order” of the *q*-profile. Can be 0, 1, or 2 in this work
*R*	The number of shared alleles between localities 1 and 2
*RBC*	Relative Bray-Curtis. Diversity measure–Eq 28
*S*_1_, *S*_2_, *S*_*tot*_	Number of alleles at a locus in locality 1, locality 2, and in the metapopulation containing both localities (for biallelic loci, this can only be 1 or 2).
*step*	Intensity of step (0 –linear, 1 –gentle step, 5 –moderate step, 50 –steep step). Used as input to simulate gradient. Eq 29.

These *H* measures could then be used in Eqs 4–6 to obtain the ‘effective-number’ diversity measures (*D* measures). This is the number of equally frequent alleles that would be needed to give the same *H* value as the alleles identified in the genetic dataset (which are usually not equally frequent).

$${}^{0}D_{\alpha }={}^{0}H_{\alpha }+1$$ (4)

$${}^{1}D_{\alpha }=e^{{}^{1}H_{\alpha }}$$ (5)

$${}^{2}D_{\alpha }=1/\left(1-{}^{2}H_{\alpha }\right)$$ (6)

To calculate pairwise beta diversities for *q* = 0, 1 and 2, we used minor allele proportions of each locality (*p*_1_ and *p*_2_), and the average minor allele proportion across localities (*p*_*av*_). We then used the alpha diversity equation for each value of *q* (1–3) to calculate the mean alpha diversity of each locus across the two localities (Eq 7) and the gamma diversity of the locus when considering the two localities as a single homogeneous population (Eq 8).

$$\bar{{}^{q}H_{\alpha }}=\frac{{}^{q}H_{\alpha .p1}+{}^{q}H_{\alpha .p2}}{2}$$ (7)

$${}^{q}H_{\gamma }={}^{q}H_{\alpha .pav}$$ (8)

Each beta diversity metric was calculated in two ways: (1) by averaging final beta values across loci (‘*AvLast*’ variant) and (2) by averaging mean alpha diversities and gamma diversities over loci (*L*) before calculating the final beta value (‘*AvFirst*’ variant). These variants give slightly different means and variances (see Supplementary Information S1 File for variance calculations).

For *q* = 0 beta measures, both Jaccard (Eqs 9 and 10) and Sorenson (Eqs 11 and 12) dissimilarity measures were calculated using ‘*R*’, the number of shared alleles between localities 1 and 2.

Jaccard:

$${}^{0}H_{\beta .Jaccard.AvLast}=\frac{1}{L}\overset{L}{\underset{i=1}{\sum }}\left(1-\frac{R}{{}^{0}H_{\gamma }+1}\right)$$ (9)

$${}^{0}H_{\beta .Jaccard.AvFirst}=1-\frac{\left\{\frac{1}{L}\sum_{i=1}^{L}R\right\}}{\left\{\frac{1}{L}\sum_{i=1}^{L}{}^{0}H_{\gamma }\right\}+1}$$ (10)

Sorenson:

$${}^{0}H_{\beta .Sorenson.AvLast}=\frac{1}{L}\overset{L}{\underset{i=1}{\sum }}\left(1-\frac{R}{\bar{{}^{0}H_{\alpha }+1}}\right)$$ (11)

$${}^{0}H_{\beta .Sorenson.AvFirst}=1-\frac{\left\{\frac{1}{L}\sum_{i=1}^{L}R\right\}}{\left\{\frac{1}{L}\sum_{i=1}^{L}\bar{{}^{0}H_{\alpha }+1}\right\}}$$ (12)

For *q* = 1 beta measures, both Mutual Information (Eqs 13 and 14) and Shannon differentiation (Eqs 15 and 16) were calculated.

Mutual information:

$${}^{1}H_{\beta .MI.AvLast}=\frac{1}{L}\overset{L}{\underset{i=1}{\sum }}{(}^{1}H_{\gamma }-\bar{{}^{1}H_{\alpha }})$$ (13)

$${}^{1}H_{\beta .MI.AvFirst}=\left\{\frac{1}{L}\overset{L}{\underset{i=1}{\sum }}{}^{1}H_{\gamma }\right\}-\left\{\frac{1}{L}\overset{L}{\underset{i=1}{\sum }}\bar{{}^{1}H_{\alpha }}\right\}$$ (14)

Shannon differentiation:

$${}^{1}H_{\beta .ShD.AvLast}=\frac{1}{L}\overset{L}{\underset{i=1}{\sum }}\left({}^{1}H_{\gamma }-\bar{{}^{1}H_{\alpha }}\right)/log\left(2\right))$$ (15)

$${}^{1}H_{\beta .ShD.AvFirst}=\left(\left\{\frac{1}{L}\overset{L}{\underset{i=1}{\sum }}{}^{1}H_{\gamma }\right\}-\left\{\frac{1}{L}\overset{L}{\underset{i=1}{\sum }}\bar{{}^{1}H_{\alpha }}\right\}\right)/\text{log}\left(2\right)$$ (16)

For *q* = 2 beta measures, both *G*_*ST*_ (Eqs 17 and 18) and *Jost-D* (Eqs 19 and 20) were calculated. The *Jost-D* calculation is shown for a pair of localities (*K* = 2), which is the only case used here.

*G*_*ST*_:

$${}^{2}H_{\beta .GST.AvLast}=\frac{1}{L}\overset{L}{\underset{i=1}{\sum }}(\left({}^{2}H_{\gamma }-\bar{{}^{2}H_{\alpha }}\right)/{}^{2}H_{\gamma })$$ (17)

$${}^{2}H_{\beta .GST.AvFirst}=\left(\left\{\frac{1}{L}\overset{L}{\underset{i=1}{\sum }}{}^{2}H_{\gamma }\right\}-\left\{\frac{1}{L}\overset{L}{\underset{i=1}{\sum }}\bar{{}^{2}H_{\alpha }}\right\}\right)/\left\{\frac{1}{L}\overset{L}{\underset{i=1}{\sum }}{}^{2}H_{\gamma }\right\}$$ (18)

*Jost-D*:

$${}^{2}H_{\beta .JOST.AvLast}=\frac{1}{L}\overset{L}{\underset{i=1}{\sum }}\left(\frac{{}^{2}H_{\gamma }-\bar{{}^{2}H_{\alpha }}}{1-\bar{{}^{2}H_{\alpha }}}*\frac{2}{2-1}\right)$$ (19)

$${}^{2}H_{\beta .JOST.AvFirst}=\frac{\left\{\frac{1}{L}\sum_{i=1}^{L}{}^{1}H_{\gamma }\right\}-\left\{\frac{1}{L}\sum_{i=1}^{L}\bar{{}^{1}H_{\alpha }}\right\}}{1-\left\{\frac{1}{L}\sum_{i=1}^{L}\bar{{}^{1}H_{\alpha }}\right\}}\;*\;\frac{2}{2-1}$$ (20)

When calculating the beta diversities on a *D* scale, the conversion of *H* measures to *D* measures can be performed at different stages:

A—The gamma and average alpha values are converted to *D* values (Eqs 21 and 22; *H*. *to*. *D* represents the relevant Eqs 4–6 with the same order of *q*) and then the results are substituted into Eqs 23 and 24.

$$\bar{{}^{q}D_{\alpha }}=H.to.D\left(\frac{{}^{q}H_{\alpha 1}+{}^{q}H_{\alpha 2}}{2}\right)$$ (21)

$${}^{q}D_{\gamma }=H.to.D\left({}^{q}H_{\alpha .av}\right)$$ (22)

$${}^{q}D_{\beta .A.AvLast}=\frac{1}{L}\overset{L}{\underset{i=1}{\sum }}{\{}^{q}D_{\gamma }/\bar{{}^{q}D_{\alpha }}\}$$ (23)

$${}^{q}D_{\beta .A.AvFirst}=\left\{\frac{1}{L}\overset{L}{\underset{i=1}{\sum }}{}^{q}D_{\gamma }\right\}/\left\{\frac{1}{L}\overset{L}{\underset{i=1}{\sum }}\bar{{}^{q}D_{\alpha }}\right\}$$ (24)

B—We calculate the relevant *H* beta diversity (Jaccard, Mutual Information, and Jost-D for to *q* = 0, *q* = 1 and *q* = 2 respectively; Eqs 9, 13 and 19) then convert to *D* using Eqs 4–6 (Eqs 25 and 26).

$${}^{q}D_{\beta .B.AvLast}=\frac{1}{L}\overset{L}{\underset{i=1}{\sum }}H.to.D\;\left({}^{q}H_{\beta .AvLast}\right)$$ (25)

$${}^{q}D_{\beta .B.AvFirst}=H.to.D(\frac{1}{L}\overset{L}{\underset{i=1}{\sum }}{}^{q}H_{\beta .AvLast})$$ (26)

As another beta measure of differentiation, we also use Bray-Curtis (Eq 27), which is the average absolute difference in allele proportion between localities (this is also sometimes called AFD [23]). Bray-Curtis is not directly related to the *q* measures but has been proposed as a straightforward measure of genetic differentiation for biallelic data, and takes the same form as the well-known Bray-Curtis measure in ecology [25,26].

$$BC=\frac{1}{L}\overset{L}{\underset{i=1}{\sum }}|p_{1}-p_{2}|$$ (27)

In addition to the above measures, previous analyses have compared beta diversity to alpha diversity, usually when searching for loci under selection (for example [27]). We have called these ‘relative beta measures’, and they are obtained by taking each *AvLast* beta diversity measure and dividing it by the average alpha diversity of the two localities. To calculate relative beta for Bray-Curtis, we divided the absolute minor allele proportion difference by the average minor allele proportion (Eq 28).


$$RBC=\frac{1}{L}\overset{L}{\underset{i=1}{\sum }}\begin{array}{c} \frac{\left|\left(p_{1}-p_{2}\right)\right|}{p_{av}} \end{array}$$ (28)

### Simulated data

**Fig 1:**
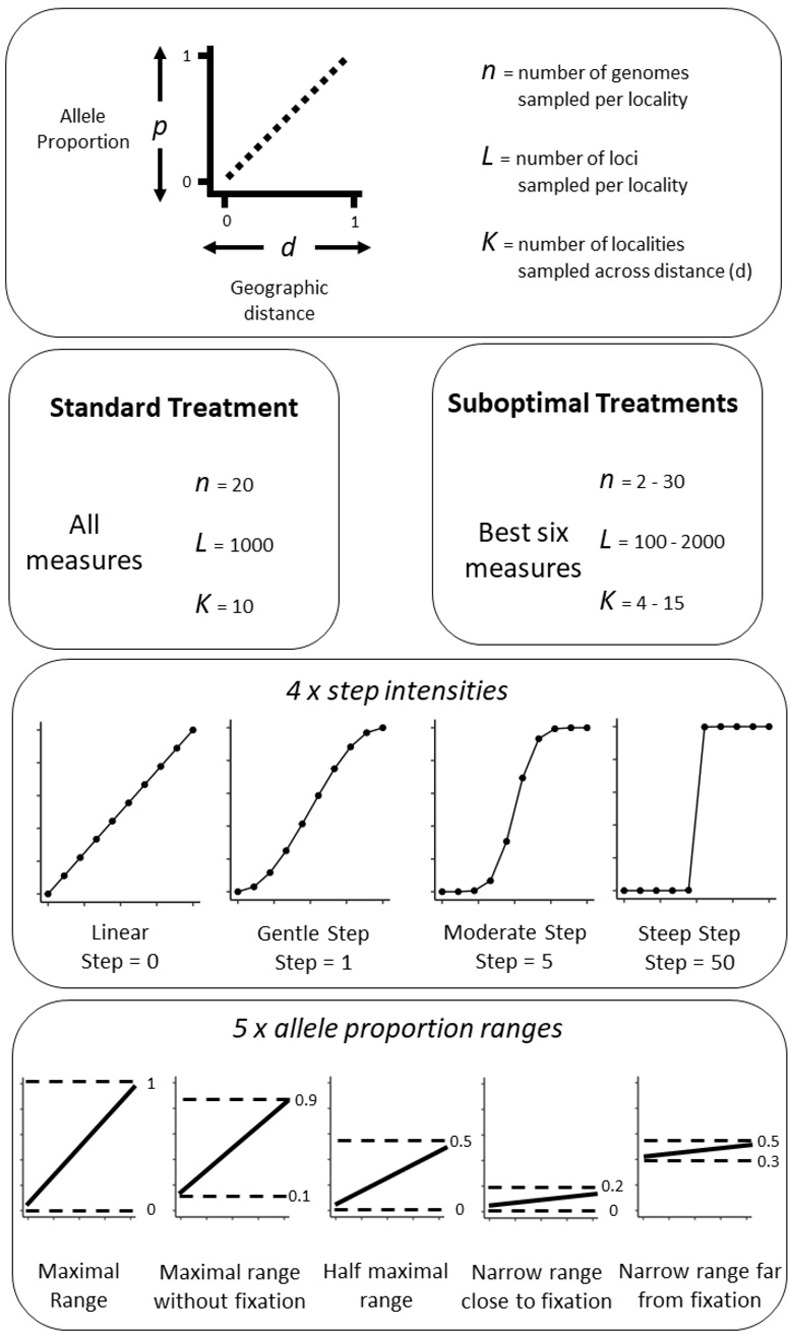
The experimental design used for this study.

Our simulated data assumed a continuous population across a linear landscape (from distance *d* = 0 to *d* = 1), with equal allele proportions (*p*) for all loci at the same location along the landscape (*d*), see Fig 1. We specified a starting allele proportion (*p*_*start*_) at distance *d* = 0 and an end allele proportion (*p*_*end*_) at distance *d* = 1. To simulate different intensities of step, we used the *qbeta* function in “stats” package in R [28], with the shape parameters $a=b=\frac{1}{1+\text{step}}$ (note that the *qbeta* function is unrelated to either the *q*-profile or beta diversity, instead it refers to the quantile function of a beta distribution). The function can be summarised as being the allele proportion at a certain distance *p*_*d*_, which is the value of *x* which satisfies *d* = *I*_*x*_(*a*,*b*) (Eq 29), where a = b = $\frac{1}{1+\text{step}}$ and 0 ≥ *d* ≥ 1, and Γ(*a*) is a gamma function. The function was multiplied by the total range of allele proportions (*p*_*end*_—*p*_*start*_) and added to the starting allele proportion (*p*_*start*_).

$$d=I_{x}\left(a,\;b\right)=\int_{0}^{x}t^{a-1}{\left(1-t\right)}^{b-1}dt/\begin{array}{c} \frac{\Gamma \left(a\right)\Gamma \left(b\right)}{\Gamma \left(a,\;b\right)} \end{array}$$ (29)

where *x* = *p*_*d*_

This created a function of allele proportion (*p*_*d*_) from a specified starting allele proportion (*p*_*start*_) at distance = 0 to a specified end allele proportion (*p*_*end*_) at distance = 1. When the step parameter was 0, the gradient was linear, and as the step parameter increased, the gradient was increasingly sigmoidal (while the step value could be infinite, a value of 50 was enough to create an abrupt step, see Fig 1).

Each *sampled* allele proportion per locus per locality was taken from a binomial distribution around the true simulated allele proportion from Eq 29. We used the *rbinom* function in R to obtain the sampled allele proportion of each locus at each locality, using the true allele proportion (*p*_*d*_, Eq 29) as the probability of success and averaging over the number samples taken (*n*). This resulted in a variable sampled allele proportion for each locus, despite being drawn from identical allele proportions.

Each simulated model took the following variables: number of localities (*K*), starting allele proportion (*p*_*start*_), end allele proportion (*p*_*end*_), number of loci (*L*), number of genome samples (*n*), and intensity of step (*step*). For each set of simulations, we tested four intensities of *step*: 0 (linear gradient), 1 (gentle step), 5 (moderate step), 50 (steep step). We also tested five different *p*_*start*_ to *p*_*end*_ ranges, allowing us to understand the beta measures’ properties over different magnitudes and positions of allele proportions:

- Maximal range: *p*_*start*_ = 0, *p*_*end*_ = 1
- Maximal range without fixation: *p*_*start*_ = 0.1, *p*_*end*_ = 0.9
- Half-maximal range: *p*_*start*_ = 0, *p*_*end*_ = 0.5
- Narrow range near fixation:*p*_*start*_ = 0, *p*_*end*_ = 0.2
- Narrow range far from fixation: *p*_*start*_ = 0.3, *p*_*end*_ = 0.5

The **standard treatment** was run 100 times with each combination of step intensity and allele proportion ranges with the default variables: *n* = 20, *L* = 1000, *K* = 10.

Next, for the **suboptimal treatments**, we individually varied the following variables to determine the sensitivity of each diversity measure:

- Number of genomes sampled (*n*), from 2 to 30 (in increments of 2).
- Number of loci (*L*), from 100 to 2000 (in increments of 100).
- Number of evenly spaced localities (*K*), from 4 to 15.

Only those measures that exceed a minimum set of criteria (high true positive rate, low false-positive rate, see below) have their results of varying genome sample size/loci/localities reported.

### Step detection

For the beta diversity measures, there were two possible criteria for detection of a step: (1) if the beta diversity between two adjacent localities was significantly higher than both the beta diversity of the previous adjacent locality pair and the beta diversity of the next adjacent locality pair (Figure S2.1A in S2 File); (2) if the beta diversities between the two adjacent pairs of localities were not significantly different, but the beta diversity of that pair of localities was significantly higher than the beta diversity of the previous and next adjacent locality pair (Figure S2.1B in S2 File). We defined significance as having a p-value less than 0.05 in a t-test (code in Supplemental Information S4 File). When a step was detected, we recorded its location on the linear landscape as the range between the localities of the highest beta or the range between the furthest localities of the highest two betas (for case (1) and case (2) respectively).

To quantify the sensitivity of each beta diversity measure in detecting a step, we tallied out of 100 the number of times: a step was detected at the correct location (true positive); a step was detected at the incorrect location (false positive); no step was detected when no step was present (true negative) and; no step was detected when one was present (false negative). We presented true and false positives in our figures and tables, with the remainder of simulations being negatives (true for linear treatments, and false for step treatments).

## Results

### Step detection sensitives under standard conditions (fixed n, L and K)

We could not use alpha diversity measures to detect steps because measures of alpha diversity had a strong dependence on allele proportion. Diversity peaked at an allele proportion of *p* = 0.5, regardless of the intensity of the step (see Figure S2.2 in S2 File and Discussion). Therefore no step detection results are presented here. No relative beta measure (including relative Bray-Curtis) was able to reliably detect a step under any condition, even when the step was steep.

Overall ***q* = 0 beta measures** were not reliably able to detect steps, instead detecting peaks in beta diversity when allele proportions departed from fixation. In the *maximal range* (*p* = 0–1) and *half-maximal range* (*p* = 0–0.5) treatments, *q* = 0 measures correctly detected steep steps (Step = 50; 100% true positives), and showed no false positives on linear gradients (Step = 0; Table 2). However, when the step was moderate (Step = 5), *q* = 0 measures consistently detected a step at an incorrect location (false positives = 100%; Table 2). When individual simulations were examined, this incorrect step was seen to be two peaks of beta diversity in the *maximal range* treatment, and a single peak in the asymmetrical *half-maximal range* treatment (Figure S2.3 in S2 File). Further, *q* = 0 measures could not detect steps in the *narrow range far from fixation* (*p* = 0.3–0.5) treatment (Table 2), and only rarely detected steps in the *maximal range without fixation* (*p* = 0.1–0.9) treatment (specifically the *AvLast* variants; Table 2).

**Table 2:** Step detection sensitivity of all diversity measures for standard conditions across five allele proportion treatments (*p* = 0–1, *p* = 0.1–0.9, *p* = 0–0.5, *p* = 0–0.2, *p* = 0.3–0.5), each with four step treatments (linear– 0, gentle step– 1, moderate step– 5, steep step– 50). Both true positives (where a step was detected at the correct location, *d* = 0.5) and false positives (where a step is detected at the wrong location or any location for the linear treatments) are shown. The remaining values (out of 100) for each treatment were simulations where no step was detected (true negatives for the linear treatments, false negatives for the step treatments). Darker blue represent higher numbers of true positives out of 100 simulations, darker red represents higher numbers of false positives out of 100 simulations.

		Maximal range: *p* = 0–1				Maximal range without fixation: *p* = 0.1–0.9				Half maximal range: *p* = 0–0.5				Narrow range near fixation: *p* = 0–0.2				Narrow range far from fixation: *p* = 0.3–0.5			
Measure	Type of Step	0	1	5	50	0	1	5	50	0	1	5	50	0	1	5	50	0	1	5	50
	**q = 0 Measures**																				
^0^ *H*_*β*.*Jac*.*AvLast*_	True Positives	-	0	0	100	-	0	0	3	-	0	0	100	-	0	0	100	-	0	0	1
	False Positives	0	99	100	0	0	0	1	1	0	100	100	0	100	100	100	0	0	0	0	0
^0^ *H*_*β*.*Sor*.*AvLast*_	True Positives	-	0	0	100	-	0	0	22	-	0	0	100	-	0	0	100	-	0	0	1
	False Positives	0	99	100	0	0	0	1	1	0	100	100	0	100	100	100	0	0	0	0	0
^0^ *D*_*β*.*A*.*AvLast*_	True Positives	-	0	0	100	-	0	0	22	-	0	0	100	-	0	0	100	-	0	0	1
	False Positives	0	99	100	0	0	0	1	1	0	100	100	0	100	100	100	0	0	0	0	0
^0^ *D*_*β*.*B*.*AvLast*_	True Positives	-	0	0	100	-	0	0	3	-	0	0	100	-	0	0	100	-	0	0	1
	False Positives	0	99	100	0	0	0	1	1	0	100	100	0	100	100	100	0	0	0	0	0
^0^ *H*_*β*.*Jac*.*AvFirst*_	True Positives	-	0	0	100	-	0	0	2	-	0	0	100	-	0	0	100	-	0	0	1
	False Positives	0	0	100	0	0	0	1	1	0	100	100	0	80	100	100	0	0	0	0	0
^0^ *H*_*β*.*Sor*.*AvFirst*_	True Positives	-	0	0	100	-	0	0	0	-	0	0	100	-	0	0	100	-	0	0	0
	False Positives	0	0	100	0	0	0	0	0	0	100	100	0	0	24	100	0	0	0	0	0
^0^ *D*_*β*.*a*.*AvFirst*_	True Positives	-	0	0	100	-	0	0	0	-	0	0	100	-	0	0	100	-	0	0	1
	False Positives	0	0	100	0	0	0	0	0	0	100	100	0	7	100	100	0	0	0	0	0
^0^ *D*_*β*.*B*.*AvFirst*_	True Positives	-	0	0	100	-	0	0	2	-	0	0	100	-	0	0	100	-	0	0	1
	False Positives	0	0	100	0	0	0	1	1	0	100	100	0	80	100	100	0	0	0	0	0
	**q = 1 Measures**																				
^1^ *H*_*β*.*MI*.*AvLast*_	True Positives	-	0	100	100	-	6	100	100	-	0	100	100	-	14	100	100	-	1	51	100
	False Positives	0	30	0	0	2	3	0	0	10	66	0	0	3	7	0	0	0	2	0	0
^1^ *H*_*β*.*ShD*.*AvLast*_	True Positives	-	0	100	100	-	6	100	100	-	0	100	100	-	14	100	100	-	1	51	100
	False Positives	0	30	0	0	2	3	0	0	10	66	0	0	3	7	0	0	0	2	0	0
^1^ *D*_*β*.*A*.*AvLast*_	True Positives	-	0	100	100	-	7	100	100	-	0	100	100	-	14	100	100	-	1	51	100
	False Positives	0	28	0	0	2	3	0	0	13	66	0	0	3	6	0	0	0	2	0	0
^1^ *D*_*β*.*B*.*AvLast*_	True Positives	-	0	100	100	-	7	100	100	-	0	100	100	-	14	100	100	-	1	51	100
	False Positives	0	28	0	0	2	3	0	0	13	66	0	0	3	6	0	0	0	2	0	0
^1^ *H*_*β*.*MI*.*AvFirst*_	True Positives	-	0	100	100	-	0	100	100	-	0	100	100	-	0	0	100	-	0	0	100
	False Positives	0	0	0	0	0	0	0	0	0	0	0	0	0	0	0	0	0	0	0	0
^1^ *H*_*β*.*ShD*.*AvFirst*_	True Positives	-	0	100	100	-	0	100	100	-	0	100	100	-	0	0	100	-	0	0	100
	False Positives	0	0	0	0	0	0	0	0	0	0	0	0	0	0	0	0	0	0	0	0
^1^ *D*_*β*.*a*.*AvFirst*_	True Positives	-	0	100	100	-	0	100	100	-	0	100	100	-	0	0	100	-	0	0	100
	False Positives	0	0	0	0	0	0	0	0	0	0	0	0	0	0	0	0	0	0	0	0
^1^ *D*_*β*.*B*.*AvFirst*_	True Positives	-	0	100	100	-	0	100	100	-	0	100	100	-	0	0	100	-	0	0	100
	False Positives	0	0	0	0	0	0	0	0	0	0	0	0	0	0	0	0	0	0	0	0
	**q = 2 Measures**																				
^2^ *H*_*β*.*GST*.*AvLast*_	True Positives	-	4	100	100	-	8	100	100	-	2	100	100	-	5	100	100	-	1	52	100
	False Positives	0	12	0	0	3	3	0	0	19	11	0	0	1	0	0	0	0	2	0	0
^2^ *H*_*β*.*JOST*.*AvLast*_	True Positives	-	98	100	100	-	84	100	99	-	0	100	100	-	0	0	100	-	1	37	100
	False Positives	36	0	0	0	17	0	0	1	1	10	0	0	0	0	1	0	0	0	1	0
^2^ *D*_*β*.*A*.*AvLast*_	True Positives	-	98	100	100	-	84	100	99	-	0	100	100	-	0	0	100	-	1	37	100
	False Positives	38	0	0	0	14	0	0	1	0	10	0	0	0	0	1	0	0	0	1	0
^2^ *D*_*β*.*B*.*AvLast*_	True Positives	-	98	100	100	-	83	100	99	-	0	100	100	-	0	0	100	-	1	36	100
	False Positives	40	0	0	0	13	0	0	1	0	8	0	0	0	0	1	0	0	0	1	0
^2^ *H*_*β*.*GST*.*AvFirst*_	True Positives	-	0	100	100	-	0	100	100	-	0	0	100	-	0	0	100	-	0	0	100
	False Positives	0	0	0	0	0	0	0	0	0	0	0	0	0	0	0	0	0	0	0	0
^2^ *H*_*β*.*JOST*.*AvFirst*_	True Positives	-	100	100	100	-	98	100	100	-	0	100	100	-	0	0	100	-	6	83	100
	False Positives	75	0	0	0	71	0	0	0	2	37	0	0	0	0	0	0	8	16	1	0
^2^ *D*_*β*.*A*.*AvFirst*_	True Positives	-	85	100	100	-	44	100	100	-	0	100	100	-	0	0	100	-	0	0	100
	False Positives	1	0	0	0	0	0	0	0	0	0	0	0	0	0	0	0	0	0	0	0
^2^ *D*_*β*.*B*.*AvFirst*_	True Positives	-	100	100	100	-	98	100	100	-	0	100	100	-	0	0	100	-	6	85	100
	False Positives	80	0	0	0	78	0	0	0	3	49	0	0	0	0	0	0	14	22	2	0
	**Bray-Curtis**																				
Bray-Curtis	True Positives	-	75	100	100	-	54	100	99	-	1	100	100	-	0	1	100	-	2	44	100
	False Positives	13	0	0	0	4	2	0	1	0	5	0	0	0	0	1	0	0	0	0	0

The ***q* = 1 beta measures** were all reliably able to detect steps across most allele proportion treatments (Table 2), although there was a clear difference between *AvFirst* and *AvLast* variants. *AvLast* variants were able to detect gentler steps (step = 1) but detected false positives in some treatments (Table 2). In contrast, *AvFirst* variants did not detect any false positives but were not as sensitive to weaker steps (Table 2).

The *AvLast* variants of *q* = 1 beta measures all had similar step detection properties to each other, with ^1^*H*_*β*.*MI*.*AvLast*_ and ^1^*H*_*β*.*ShD*.*AvLast*_ having identical properties, as did ^1^*D*_*β*.*A*.*AvLast*_ and ^1^*D*_*β*.*B*.*AvLast*_. A step was detected 100% of the time in all steep and moderate step treatments (step = 5, 50) except the *narrow range far from fixation* treatment, where a step was detected 51% of the time (Table 2). For the gentle step treatments (step = 1), the *AvLast* variants of *q* = 1 beta measures sometimes detected a step at the correct location but more often detected false positives (Table 2); this false-positive rate was as high as 66 out of 100 simulations in the *half-maximal range* treatment (Table 2). For the linear treatments (step = 0), false positives were detected 10–13% of the time by the *AvLast* variants of *q* = 1 beta measures in the *half-maximal range* treatment and 0–3% of the time for other allele proportion treatments (Table 2).

Each of the *AvFirst* variants of *q* = 1 beta measures had identical step detection sensitivities across allele proportion treatments. A step was detected 100% of the time in all steep step treatments, and 0% of the time in the linear gradient treatments (Table 2). A moderate step was detected 100% of time for both *maximal range* treatments and the *half maximal range* treatment, but 0% of the time for both *narrow range* treatments (Table 2). None of the *AvFirst* variants of *q* = 1 beta measures detected a gentle step under any of the treatments (Table 2). Further, none of the *AvFirst* variants of *q* = 1 beta measures detected any false positives under any of the treatments under standard conditions (Table 2).

The ability of ***q* = 2 beta measures** to reliably detect steps was mixed, with some measures able to detect steps over most conditions, and others not reliably able to detect steps. The *G*_*ST*_ based measures had desirable step detection properties similar to *q* = 1 measures, and ^2^*D*_*β*.*A*.*AvFirst*_ had the best step detection properties amongst measures we tested under standard conditions (Table 2). The remaining measures (^2^*H*_*β*.*JOST*.*AvLast*_, ^2^*D*_*β*.*A*.*AvLast*,_^2^*D*_*β*.*B*.*AvLast*_, ^2^*H*_*β*.*JOST*.*AvFirst*_ and ^2^*D*_*β*.*B*.*AvFirst*_) were not suitable for step detection due to their high rates of false positives in linear treatments (Table 2).

The two *G*_*ST*_ measures had similar step detection properties to *q* = 1 measures, with similar differences between *AvFirst* and *AvLast* variants. ^2^*H*_*β*.*GST*.*AvFirst*_ differed from the *AvFirst* variants of *q* = 1 measures by not detecting moderate steps in the *half-maximal range* treatment (Table 2). As with the *q* = 1 measures, ^2^*H*_*β*.*GST*.*AvLast*_ was more sensitive to gentler steps than the *AvFirst* variant (^2^*H*_*β*.*GST*.*AvFirst*_), correctly detecting a moderate step 100% of the time in the *half-maximal range* and *narrow range near fixation* treatments and 52% of the time for the *narrow range far from fixation* treatment (Table 2). However, ^2^*H*_*β*.*GST*.*AvLast*_ occasionally detected false positives when the gradient was linear including 19% of the time in the *half-maximal range* treatment. ^2^*H*_*β*.*GST*.*AvLast*_ also detected false positives in the gentle step treatments for the larger allele proportion range treatments (Table 2).

The step detection properties of ^2^*H*_*β*.*JOST*.*AvLast*_, ^2^*D*_*β*.*A*.*AvLast*_ and ^2^*D*_*β*.*B*.*AvLast*_ were near-identical, but poor overall. These measures were prone to high rates of false positives in the two maximal range treatments (Table 2). In the *half-maximal range* treatment, there were no false positives in linear treatment (0–1%), but false positives were detected in the gentle step treatment 8–10% of the time (Table 2). ^2^*H*_*β*.*JOST*.*AvFirst*_ and ^2^*D*_*β*.*B*.*AvFirst*_ behaved similarly to their *AvLast* variants, but with even higher rates of false positives (Table 2). They detected false positives 75–80% of the time for the *maximal range* treatment and 71–78% of the time for the *maximal range without fixation* treatment. These two measures also detected false positives in the gentle step treatment 37–49% of the time for the *half-maximal range* treatments, and 8–22% of the time for the linear and gentle step treatments in the *narrow range far from fixation* treatment (Table 2).

Interestingly, the ^2^*D*_*β*.*A*.*AvFirst*_ measure does not have the same poor properties as the other *AvFirst* variants of *q* = 2 beta measures under standard conditions. It detected a large step 100% of the time and had a false positive rate of 0–1% all allele proportion treatments (Table 2). It detected a moderate step 100% of the time in the *maximal range* treatments and the *half-maximal range* treatments (Table 2). Further, ^2^*D*_*β*.*A*.*AvFirst*_ correctly detected the gentle step 44% of the time in the *maximal range without fixation* treatment and 85% of the time in the *maximal range* treatment. Notably, this result occurred when the allele proportion treatment was symmetrical over *p* = 0.5. When looking at the simulations under standard conditions alone, ^2^*D*_*β*.*A*.*AvFirst*_ appears to have the best step detection properties of all the measures we tested (Table 2).

The **Bray-Curtis beta diversity measure** was more sensitive to gentler steps than most other measures, including all *q* = 0 and *q* = 1 measures, but was also prone to false positives when the allele proportion range was large (Table 2). In that respect, it was similar to other the *q* = 2 beta measures, but with a lower false-positive rate (Table 2). Bray-Curtis detected a gentle step 75% of the time in the maximal range treatment and 54% of the time in the maximal range without fixation treatment (Table 2). As with *q* = 2 measures, this sensitivity may also be because of a symmetry of adjacent beta values around *p* = 0.5. Despite the occasional detection of false steps, we do not rule out Bray-Curtis as an effective measure for detecting steps.

### Comparison of the six best candidate measures under suboptimal conditions

Based on the highest true positive rates and lowest false positive and negative rates under standard conditions, we selected six of the 25 metrics: ^1^*H*_*β*.*MI*.*AvLast*_, ^1^*H*_*β*.*MI*.*AvFirst*_, ^2^*H*_*β*.*GST*.*AvLast*_, ^2^*H*_*β*.*GST*.*AvFirst*_, ^2^*D*_*β*.*A*.*AvFirst*_, and Bray-Curtis. We chose ^2^*H*_*β*.*GST*.*AvFirst*_ instead of ^2^*H*_*β*.*JOST*.*AvFirst*_, as the false positive rates of the latter were too high. We used ^1^*H*_*β*.*MI*.*AvLast*_ and ^1^*H*_*β*.*MI*.*AvFirst*_ to represent the *AvLast* and *AvFirst* variants of *q* = 1, though our observations should apply to any of the *q* = 1 measures we tested because results were near identical for all the *AvLast q* = 1 measures and for all the *AvFirst q* = 1 measures (Table 2).

**Fig 2:**
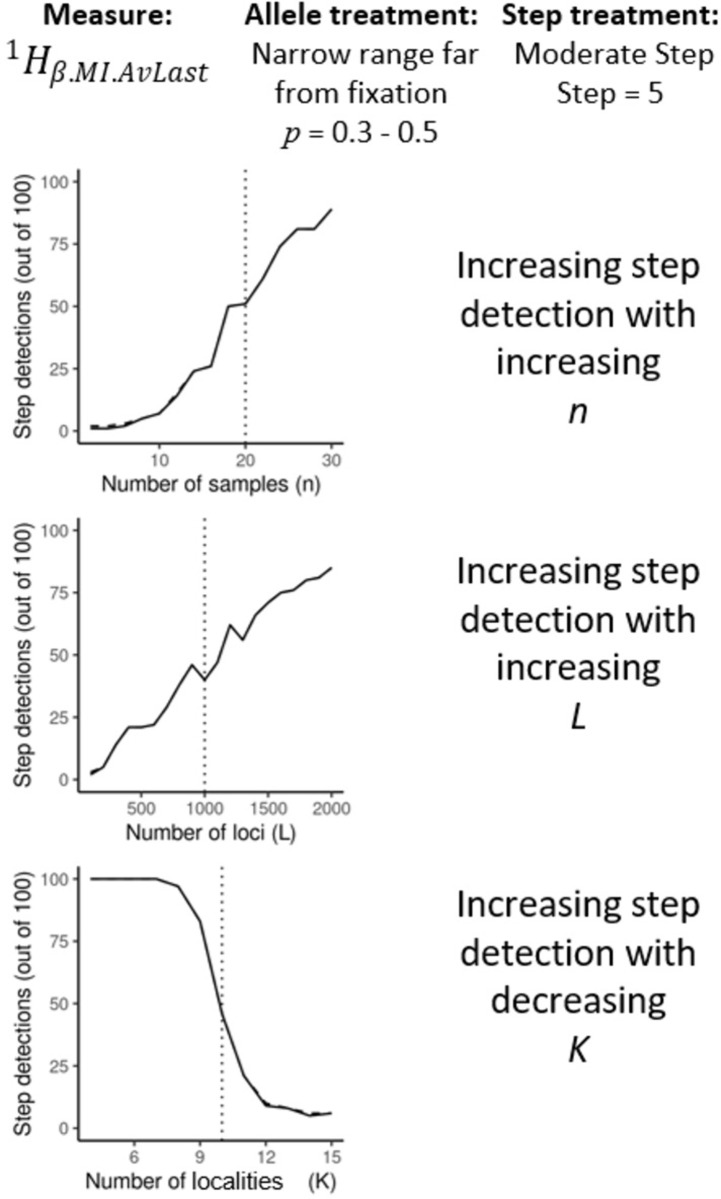
Example of how true positives generally respond to the varied number of genomes (*n*), number of loci (*L*) and number of localities (*K*). The dashed line indicates the values used in the standard treatments. These results were taken from an *AvLast* variant of a *q* = 1 measure under the moderate step treatment (*step* = 5) and the *narrow range far from fixation* allele proportion treatment (*p* = 0.3–0.5). For full results for the six chosen measures and treatments, see Supplement S3 File.

Broadly, step detection sensitivity (as measured by number of true positives) increased with increasing number of genomes sampled (*n*), increasing number of loci sampled (*L*) and decreasing number of localities sampled (*K;* Fig 2). This reflected, respectively, in: the increased precision of individual allele proportion measurement, decreased variance between loci, and increased absolute allele proportion difference between localities. While these general trends were expected, these properties were not consistent amongst candidate measures nor consistent across allele proportion treatments. Table 3 summarises the trends for the six chosen measures. For a more detailed comparison of these measures detailing the individual effects of genome sample size, number of loci and number of localities see Supplemental Information S3 File.

**Table 3:** Step detection sensitivity, averaged over all optimal and suboptimal conditions, for the best six diversity measures (Mutual Information *AvLast* variant, Mutual Information *AvFirst* variant, *G*_*ST*_ *AvLast* variant, *G*_*ST*_ *AvFirst* variant, ^2^*D*_*β*.*A*.*AvFirst*_, and Bray-Curtis) across five allele proportion treatments (*p* = 0–1, *p* = 0.1–0.9, *p* = 0–0.5, *p* = 0–0.2, *p* = 0.3–0.5), each with four step treatments (linear– 0, gentle step– 1, moderate step– 5, steep step– 50). Both true positives (where a step was detected at the correct location, d = 0.5) and false positives (where a step is detected at the wrong location or any location for the linear treatments) are shown. Values are in percentages, averaged across all tested simulations (where genome sample size, number of loci and number of localities was varied. Remaining percentages (out of 100) for each treatment were simulations where no step was detected (true negatives for the linear treatments, false negatives for the step treatments). Darker green represents higher percentages of true positives, darker red represents higher percentages of false positives. Comprehensive data are shown in Supplement S3 File.

		Maximal range: *p* = 0–1				Maximal range without fixation: *p* = 0.1–0.9				Half maximal range: *p* = 0–0.5				Narrow range near fixation: *p* = 0–0.2				Narrow range far from fixation: *p* = 0.3–0.5			
Measure	Type of Step	0	1	5	50	0	1	5	50	0	1	5	50	0	1	5	50	0	1	5	50
^1^ *H*_*β*.*MI*.*AvLast*_	True Positives	-	7.1	99.8	100	-	11.9	99.8	99.8	-	7.1	95.8	100	-	19.9	88.9	96.6	-	3.9	46.5	98.3
	False Positives	3.8	25.2	0	0	1.5	3.5	0	0.2	12.7	44.8	1.2	0	6.5	5.6	0.2	0	0.8	0.7	0.2	0.2
^1^ *H*_*β*.*MI*.*AvFirst*_	True Positives	-	5.2	95.7	100	-	5.3	95.9	100	-	5.6	84.2	100	-	1.2	6.8	92	-	0	4.3	82.6
	False Positives	0	0	0	0	0	0	0	0	0	0	0	0	0	0	0	0	0	0	0	0
^2^ *H*_*β*.*GST*.*AvLast*_	True Positives	-	12.7	100	100	-	16.3	100	99.8	-	11.2	96.3	100	-	10.3	84.2	96.1	-	4.2	47.9	98.3
	False Positives	2.6	5.6	0	0	1.9	2.5	0	0.2	10.6	9.2	0.1	0	2	0.9	0.2	0	0.8	0.7	0.2	0.2
^2^ *H*_*β*.*GST*.*AvFirst*_	True Positives	-	4.5	89.6	89.6	-	5.3	92.4	100	-	0	4.4	100	-	0	0	82.6	-	0	4.3	82.5
	False Positives	0	0	0	0	0	0	0	0	0	0	0	0	0	0	0	0	0	0	0	0
^2^ *D*_*β*.*A*.*AvFirst*_	True Positives	-	73	100	100	-	42.3	100	100	-	6.6	73.4	100	-	0	1.1	64	-	0.4	6.4	92
	False Positives	9	0	0	0	5.6	0	0	0	0	0	0	0	0	0	0	0	0	0	0	0
Bray-Curtis	True Positives	-	68.4	100	100	-	53.4	100	99.7	-	11	94	100	-	1.9	15.8	95.4	-	4.1	45.4	98
	False Positives	10.9	0.2	0	0	7.6	0.3	0	0.3	1.1	6.9	0.2	0	0	0.4	0.9	0.1	1	0.8	0.2	0.3

When comparing the six candidate beta measures over the *maximal range* allele distribution treatment, Bray-Curtis and ^2^*D*_*β*.*A*.*AvFirst*_ were most sensitive to smaller steps but are prone to detecting false steps with fewer localities sampled (S3.1 in S3 File). This is likely due to their convex relationship with allele proportion even when no step was present. In other words, the adjacent beta diversity of these measures was dependent on allele proportion and peaked at *p* = 0.5. Additionally, ^2^*H*_*β*.*GST*.*AvFirst*_ appeared to have a much higher standard error than the other measures (Supplement S3.1 in S3 File), had lower rates of true positive detection (Table 3 and S3.1 in S3 File), and had inconsistent step detection rates depending on whether the number of localities was even (Supplement S3.1 in S3 File). In the linear and gentle step treatments ^1^*H*_*β*.*MI*.*AvLast*_ and, to a lesser extent, ^2^*H*_*β*.*GST*.*AvLast*_ were prone to detecting false positives (Table 3 and S3.1 in S3 File).

The patterns in the *maximal range without fixation* treatment largely aligned with the *maximal range* treatment, except that in the former, ^2^*H*_*β*.*GST*.*AvFirst*_ showed marginally better performance and the false positive detections of ^1^*H*_*β*.*MI*.*AvLast*_ were greatly reduced (Table 3 and S3.2 in S3 File).

The *half-maximal range* treatment, which is not symmetrical over *p* = 0.5, did not show the same high step detection sensitivities of Bray-Curtis and ^2^*D*_*β*.*A*.*AvFirst*_ as the *maximal range* treatments (Table 3 and S3.3 in S3 File). However, unlike ^2^*D*_*β*.*A*.*AvFirst*_, which was the least sensitive to detecting steps (i.e. had the lowest rate of true positive detection; Table 3), Bray-Curtis remained as sensitive to detecting steps as the other measures (Table 3 and S3.3 in S3 File). This allele proportion treatment also highlighted the problematic false positive detection properties of ^1^*H*_*β*.*MI*.*AvLast*_ and to lesser extent ^2^*H*_*β*.*GST*.*AvLast*_ (Table 3 and S3.3 in S3 File).

In the *narrow range close to fixation* treatment ^2^*H*_*β*.*GST*.*AvLast*_ and ^1^*H*_*β*.*MI*.*AvLast*_ performed notably better than the other measures (having higher rates of true positive detection; Table 3) but had problems with rates of false-positive detection (Table 3 and S3.4 in S3 File). ^2^*H*_*β*.*GST*.*AvFirst*_ and ^1^*H*_*β*.*MI*.*AvFirst*_ performed considerably worse (in terms of true positive detection) but without detecting false positives (Table 3 and S3.4 in S3 File). ^2^*D*_*β*.*A*.*AvFirst*_ performed poorly in this allele treatment, detecting less true positives (Table 3), and Bray-Curtis had intermediate properties, detecting true positives at a rate close to the other, more sensitive, measures (Table 3 and S3.4 in S3 File).

Lastly, in the *narrow range far from fixation* allele proportion treatment, ^2^*H*_*β*.*GST*.*AvLast*_, ^1^*H*_*β*.*MI*.*AvLast*_, and Bray-Curtis all performed consistently well (in terms of true positive detection rate; Table 3) and had roughly identical step detection properties when genome sample size, number of loci and locality number was varied (S3.5 in S3 File). Comparatively ^2^*H*_*β*.*GST*.*AvFirst*_, ^1^*H*_*β*.*MI*.*AvFirst*,_ and ^2^*D*_*β*.*A*.*AvFirst*_, which also had roughly identical step detection properties to each other, were notably less sensitive to detecting steps than the other measures tested (Table 3 and S3.5 in S3 File). Further, these *AvFirst* measures had the undesirable property of being sensitive to the number of localities being even (S3.5 in S3 File).

Finally, Table 4 gives a condensed summary of the data shown in Table 3 across all simulations, plus an overview of the properties of the chosen six measures.

**Table 4:** Summary of properties of each of the six best candidate measures—Mutual Information (*AvLast* and *AvFirst* variants), G_ST_ (*AvLast* and *AvFirst* variants), ^2^D_β.A_ (*AvLast* variant) and Bray-Curtis. Note that we detail the properties of Mutual Information here, but the properties are the same for each of the other *q* = 1 measures. ‘True Positive Detections’ was calculated as the percentage of true positives across all step simulations. ‘False Positive Detections’ was calculated as the percentage of false positives across all simulations. ‘True Negative Detections’ was calculated as the percentage of true negatives across all linear simulations. Properties are shaded based on their usefulness as a step detection measure: most desirable properties (blue), undesirable properties (red).

Diversity Measure	True Positive Detection	False Positive Detection	True Negative Detection	Effect of Allele Proportion Position	Rate of true positives with narrow steps
^1^ *H*_*β*.*MI*.*AvLast*_	65.0%	5.3%	94.9%	Largely unaffected	High
^1^ *H*_*β*.*MI*.*AvFirst*_	51.9%	0.0%	100%	Largely unaffected	Moderate
^2^ *H*_*β*.*GST*.*AvLast*_	65.1%	1.8%	96.5%	Largely unaffected	High
^2^ *H*_*β*.*GST*.*AvFirst*_	43.6%	0%	100%	Largely unaffected	Low
^2^ *D*_*β*.*A*.*AvFirst*_	57.2%	0.8%	97.0%	Strong effect	Low
Bray-Curtis	65.8%	1.6%	95.8%	Affected.	High

## Discussion

### Measures most effective at detecting steps

No diversity measure was consistently best across all tested scenarios (Table 4), but some measures stood out as effective for detecting steps in genetic data. The measures most reliably able to detect genetic steps were *q* = 1 based beta measures, *G*_*ST*_ based beta measures, ^2^*D*_*β*.*A*.*AvFirst*_ and Bray-Curtis dissimilarity. Out of these, Bray-Curtis and the *AvLast* variants of *G*_*ST*_ and *q* = 1 beta measures were the most sensitive to steps overall (Table 4), whereas the *AvFirst* variant of the *q* = 1 beta measures did not detect false positives (Tables 2–4). We therefore recommend that to minimise the limitations of each measure, researchers should use a combination of these measures: one of the *AvLast* variants of *G*_*ST*_, *q* = 1 or Bray-Curtis beta measures, and one of the *AvFirst* variants of *G*_*ST*_ or *q* = 1 beta measures.

The best step detection properties were found in the ***q* = 1 beta measures** (including Shannon Differentiation and Mutual Information). While many have touted the benefit of *q* = 1 measures more broadly [29–31], our study is the first to highlight the beneficial properties of *q* = 1 for the detection of geographic genetic steps. Of these measures, there was a clear trade-off between high true positive detection and low false positive detection between the *AvFirst* and *AvLast* variants (the order of averaging beta diversity values across loci; Tables 2–4). This difference appeared to be driven by the standard error in the measures (S3 File), with the higher standard error of the *AvFirst* variants leading to the desirable property of not detecting any false positives in any of our simulations (Tables 3 and 4). However, this higher standard error also obscured smaller and gentler steps, reducing the overall rate of step detection of the *AvFirst* variants. In contrast, the *AvLast* variants had a stronger sensitivity to smaller and gentler steps (a lower false-negative rate) but were prone to detecting false positives under certain conditions (Tables 3 and 4 and S3 File). This undesirable property of the *AvLast* variants seemed to occur close to fixation. When looking at individual simulations (S3.6 in S3 File), we found that these false positives, detected when the step was gentle, had two peaks near *p* = 0 and *p* = 1 when in the *maximal range* treatment. This might indicate a similarly to *q* = 0 measures in detecting a departure from fixation (see Discussion of *q* = 0 measures below) and be a possible downside to the known property of *q* = 1 measures being more sensitive to rare alleles than *q* = 2 measures [18]. However, if the allele frequencies were more uneven between loci, as would be the case with empirical data, these minor peaks would likely not influence the overall results.

Despite the various, and valid, criticisms of *G*_*ST*_ as a diversity measure [5,21], we show that ***AvLast* variant of *G***_***ST***_ has one of the best step detection properties of the measures we have tested here (Table 4). However, because *G*_*ST*_ is most likely to be used by molecular ecologists currently, we must highlight its weaknesses more clearly–that, like the *q* = 1 beta measures, *G*_*ST*_ is prone to false step detection near fixation, especially when the number of genomes (*n*) or number of localities (*K*) sampled is low (S3 File). These properties align with studies on *G*_*ST*_ */F*_*ST*_, which found that these measures have a strong dependence on *K* and allele frequency [32–34]. Interestingly, the step detection sensitivities of *G*_*ST*_-based measures and their weaknesses are more closely aligned with *q* = 1 measures than with other *q* = 2 based measures. Specifically, the *AvLast* and *AvFirst* variants of *G*_*ST*_ have a similar trade-off in properties to the corresponding variants of *q* = 1 measures. The *AvLast G*_*ST*_ variant was more sensitive to smaller and gentler steps but was prone to sometimes detecting false positives in certain scenarios (Table 3). The rate of false positives showed a similar pattern, but was lower than the *AvLast* variant of *q* = 1, making it a better measure in that respect. The tendency for *AvLast* variant of *G*_*ST*_ to detect false positives that we found here appears to mirror the tendency for *G*_*ST*_ */F*_*ST*_ to detect false positives in outlier tests [35]. In comparison, the *AvFirst* variant of *G*_*ST*_ did not detect any false positives, but, because of its much larger standard error, was not nearly as sensitive to steps as other measures (Tables 3 and 4). This measure also had inconsistent true positive detection behaviour with the number of localities (S3 File). In empirical data one would often not know the real location of the step, so the lack of consistency of the *AvFirst* variant of *G*_*ST*_ would make it a poor measure We therefore recommend not using the *AvFirst* variant of *G*_*ST*_ for detecting steps, instead using the *AvFirst* variants of *q* = 1 measures which do not have these problems.

**Bray-Curtis dissimilarity** (also known as allele frequency difference–AFD [23]) had good step detection properties overall. Bray-Curtis aligned most to ^2^*D*_*β*.*A*.*AvFirst*_ compared to other *q* = 2 measures, and under certain conditions it aligned more with the *AvLast* variants of *G*_*ST*_ and *q* = 1 measures. While Bray-Curtis has been cited as a straightforward way to measure differences in allele proportion [23], with differences in allele proportion being equivalent regardless of proximity to fixation, we found that the measure had a dependence on allele proportion (peaking at *p* = 0.5). This undesirable property was shared with ^2^*D*_*β*.*A*.*AvFirst*_ and other *q* = 2 measures, and was an unexpected property of Bray-Curtis. This property can be explained because as the allele proportion of one or both of sites approaches 0 or 1, the range of possible Bray-Curtis values decreases, decreasing the average Bray-Curtis value. Therefore, these biases mean that *sampled* differentiation as measured by Bray-Curtis is not always equal *actual* allele proportion difference. Because we found that Bray-Curtis performed about as well as the *AvLast* variants of *q* = 1 and *G*_*ST*_ measures (Table 4), and that very few studies have compared it to other measures [24,36], this measure warrants further mathematical analysis. Further, because Bray-Curtis had a higher true positive rate and lower false positive rate than the *AvLast* variants of *q* = 1 and *G*_*ST*_ measures, we recommend its inclusion in step detection studies in general.

Lastly, the step detection properties of ^**2**^***D***_***β*.*A*.*AvFirst***_ were surprisingly good under standard conditions (Table 2), especially when contrasted with similar *q* = 2 measures. However, when we tested under suboptimal conditions, we could only identify one scenario where this measure would perform better than any of the other five candidate measures we tested, and in many scenarios, it performed worse (Tables 3 and 4 and S3 File). This one scenario was under the *maximum range* treatment, where this measure had a slightly higher true positive detection rate of gentle steps (Table 3), but only when K > ~8 (Figure S3.6.21 in S2 File). However, this is likely an artefact of a strong dependence on allele proportion position leading to high rates of step detection when the allelic range was large and over *p* = 0.5. This property plus a lower sensitivity to moderate and gentle steps overall contributed to the relative poor performance of this measure. Even though ^2^*D*_*β*.*A*.*AvFirst*_ did not have such a high standard error as the *AvFirst* variant of *G*_*ST*_, it still had higher standard error than the *AvLast* variants of measures that we tested. Despite these undesirable properties, ^2^*D*_*β*.*A*.*AvFirst*_ was still a reasonably effective measure, having high rates of true positive and true negative detection and low rates of false-positive detection (Table 4).

### Measures ineffective at detecting steps

Our findings ruled out over 40 measures for detecting steps because they either have standard errors that are too high or are too dependent on allele proportion position. These are alpha diversity measures, relative beta measures, many of the *q* = 2 based measures (including *Jost-D*) and allelic richness (*q* = 0) measures. When alpha diversity was measured along a linear gradient, diversity peaked at *p* = 0.5 for all values of *q* (Figure S2.2 in S2 File), thus having too strong of a dependence of allele proportion. This property confounds the ability to differentiate sudden allele proportion changes which characterise a step. A correction could be applied to alpha measures to counteract this problem (such as with [20]), possibly allowing for the detection of steps with alpha diversity, but this is beyond the scope this study. Also, all relative beta measures (where beta diversity was divided by alpha diversity) were not effective at detective steps largely due to poor standard error properties of ratios [37], and we recommend that they should not be used in further step detection studies. Interestingly, the excluded *q* = 2 measures had very poor step detection properties despite appearing similar to other *q* = 2 based measures that performed quite well and therefore chosen for further analysis. These poor *q* = 2 measures had a high dependence on alpha measures such as allele proportions (see [18]), leading to high rates of false positive detection. As with alpha measures, corrections could be devised to alleviate these problems.

Allelic richness related beta measures (*q* = 0) had poor step detection properties in many of our simulated scenarios, so we do not recommend their use for the detection of steps in genetic data. However, we stress that these measures should not be discounted as completely uninformative, because our results confirmed that *q* = 0 measures are useful for detecting other aspects of genetic diversity such as rates of fixation and changes in small allele proportions. In scenarios where steps were characterised by changes in allele proportion away from fixation (e.g. *p* = 0.3–0.5), *q* = 0 measures could not detect even the steepest steps (Table 2). These results are an indication of the *q* = 0 measures’ inability to differentiate between differences in allele proportion when *p*_*1*_ and *p*_*2*_ are not at fixation (0 > *p* > 1). However, when the steps were closer to fixation (*p* = 0 or 1), *q* = 0 measures could detect steep steps and detected false positives (peaks of beta diversity not at the step location) when the step was moderate (Figure S2.3 in S2 File). These peaks of beta diversity for *q* = 0 measures are likely detecting a departure from fixation rather than a step in allele proportion because we can see a single peak in the *p* = 0–0.5 treatment and two peaks in the *p* = 0–1 treatment (Figure S2.3 in S2 File). While this property is not useful for changes in allele proportions that are not fixed, with enough loci of varying proportions *q* = 0 should still be an effective step detection measure. This would especially be the case in systems where alleles are expected to be lost on one or both sides of a step (e.g. small population size, or high selection). When a biallelic locus becomes fixed, the number of the alleles will go from 2 to 1. Therefore, even the smallest change allele proportion to fixation, from *p* = 0.01 to *p* = 0 for example, would be detected most strongly with *q* = 0 measures, followed by *q* = 1 measures to a lesser extent. However, due to the inevitable incompleteness of sampling, there would rarely be any sampling that would be sufficiently accurate, and there would be many cases of false fixation.

### Guide for detecting steps

Our advice for molecular ecologists is split into two areas: how to design a study to maximise the chances of detecting a step when one is present (true positive rate); and how to choose diversity measures to maximise true positive rate while managing the false positive rate. Firstly, we emphasise the importance of a good sampling strategy in terms of maximising number of genomes sampled, number of loci and the strategic choice of localities. Our advice agrees with current guides for formulating landscape genetic studies [38,39]. Specifically, we show that there is a clear effect of number of loci sampled, but the number of loci that must be sampled to avoid this effect is much lower than values easily attainable with modern molecular studies. Therefore, it is advisable for researchers to instead maximise the number of individuals sampled from each site. A reduced number of localities is less of a constraint because a step can still be detected with a larger distance between localities on either side. However, this obviously comes at the cost of geographical precision, and the scale of possible inference [39].

Secondly, when choosing a diversity measure, we recommend the use of multiple measures in concert. Many studies already use multiple diversity measures, and others have recommended this as best practice [18,40]. Specifically, we recommend using at least one of the measures with high true positive rates (*AvLast* version of *G*_*ST*_, *AvLast* version of a *q* = 1 measure, or Bray-Curtis, Tables 3 and 4) in combination with the *AvFirst* variant of a *q* = 1 measure (such as Mutual Information), which are not prone to false-positive detection (Tables 3 and 4). Large steps will likely be detected by all measures, but when detecting smaller steps any conflicts between results should be carefully considered. For example, if a step was detected by the more sensitive *AvLast* variant of MI, but not the *AvFirst* version of MI one could infer that if there was a step present it would be small or that this is a false positive. Making this decision will be determined by how conservative one wishes to be with step detection rate and knowledge of the study system. Additionally, we advise that molecular ecologists make a prior assessment of the variance in allele frequencies in their genetic datasets (across the sampled localities) to better choose the most appropriate beta diversity measure. If possible, prior knowledge of how often certain allele frequency clines (e.g. steep or slight) are in the natural population being studied would also assist practitioners in selecting the most suitable measure.

As mentioned previously, most current methods and software exclusively use, or at least default to *q* = 2 measures (i.e., *F*_*ST*_ or *G*_*ST*_). Our study has shown that current software would benefit by integrating a wider variety of genetic diversity measures. Specifically, an *AvFirst* variant of a *q* = 1 measure could be used to decrease detections of false positives. These tools could also add the sensitive measures of Bray-Curtis and an *AvLast* variant of *q* = 1 to maximise the chances of detecting steps over a wide variety of conditions.

In conclusion, when it comes to detecting steps, we show that not all measures are equal, each having its own sensitivities and weaknesses. By using a combination of measures in concert, molecular ecologists will be able to more confidently detect and classify steps in their systems. Understanding these properties is key to reaching the correct conclusions in landscape genetic studies and improving conservation and management outcomes.

## Supporting information

S1 File Formulae for calculation of variance. (DOCX)

S2 File Extra figures. **Figure S2.1**—Graphical representation of step detection protocol. **Figure S2.2**—Dependence of alpha diversity on allele proportion. **Figure S2.3**—Effect of allele proportion on *q* = 0 measures.
(DOCX)

S3 File Expanded results: Detailed comparison of the six best candidate measures under suboptimal conditions. (DOCX)

S4 File R code used for this study (this will also be publicly available on github). (DOCX)
