# Supplementary material for: Detecting steps in spatial genetic data: Which diversity measures are best?
Source: PLoS One. 2022 Mar 14;17(3):e0265110. doi: 10.1371/journal.pone.0265110 (PMC8920294; doi:10.1371/journal.pone.0265110)
Supplement: S1 File — (DOCX) [file pone.0265110.s001.docx]

Supplemental information S1 - Formulae for calculation of variance

Calculation of variance for all “AvLast” measures was done using standard variance calculations. Below are the formulae for calculating the variance of the “AvFirst” variants of measures (Crow, Kimura, 1970). Note that these calculations are usually over-conservative, i.e. gives a max value for the variance. The following assumptions were made: the unknown covariance of R’ and S’ is zero; the variance of S’ due to variation between localities is small relative to the variance of S’ between loci and is also poorly estimated because there are only two loci.

**References**

Crow JF and Kimura M. 1970 An Introduction to Population Genetics Theory. Harper & Row New York. (Equation A.9.14, p 512).Jaccard:

$$Var \left[ {}^{0}H_{\beta.Jac.\mathrm{AvFirst}} \right]=\frac{Var\left( R^{'} \right)}{\left( G^{'}+1 \right)^{2}}+{[R'}^{2}*Var(G^{'})]/[\left( G’+1 \right)^{4}]$$

where: R’= $\left\{ \frac{1}{L}\sum_{i=1}^{L} R \right\} and G^{'}=\left\{ \frac{1}{L}\sum_{i=1}^{L} {}^{0}H_{\gamma} \right\}$

Sorenson:

$${Var(}^{0}H_{\beta.Sor.\mathrm{AvFirst}})=\frac{4\left[ Var\left( R^{'} \right) \right]}{{S^{'}}^{2}}+\frac{4\left[ {R'}^{2}Var\left( R^{'} \right) \right]}{{S^{'}}^{4}}$$

|  | Where: $R^{'}=\left\{ \frac{1}{L}\sum_{i=1}^{L} R \right\} S^{'}=\left\{ \frac{1}{L}\sum_{i=1}^{L} \bar{{}^{0}H_{\alpha}+1} \right\}$ |
| --- | --- |

Mutual information:

$$Var\left[ {}^{1}H_{\beta.MI.AvFirst} \right]=Var\left( G \right)+Var(A)$$

Where*:* $G=\left\{ \frac{1}{L}\sum_{i=1}^{L} {}^{1}H_{\gamma} \right\}and A=\left\{ \frac{1}{L}\sum_{i=1}^{L} \overline{{}^{1}H_{\alpha}} \right\}$

Shannon differentiation:

$$Var\left[ {}^{1}H_{\beta.MI.\mathrm{AvFirst}} \right]=\left[ Var\left\{ \frac{1}{L}\sum_{i=1}^{L} {}^{1}H_{\gamma} \right\}+Var\left\{ \frac{1}{L}\sum_{i=1}^{L} \overline{{}^{1}H_{\alpha}} \right\} \right]/[{(log2)}^{2}]$$

$Jost-D$:

$${Var (}^{2}H_{\beta.JOST.AvFirst})=4*\frac{\left[ Var\left( G \right) \right]}{\left[ \left( A-1 \right)^{2} \right]}+\frac{\left\{ 4*\left[ \left( G-1 \right)^{2}*var\left( A \right) \right] \right\}}{\left[ \left( 1-A \right)^{4} \right]}$$

Where: $G=\left\{ \frac{1}{L}\sum_{i=1}^{L} {}^{2}H_{\gamma} \right\}and A=\left\{ \frac{1}{L}\sum_{i=1}^{L} \overline{{}^{2}H_{\alpha}} \right\}$

$Gst$:

$${Var (Gst ie}^{2}H_{\beta.GST.AvFirst})=\frac{\left[ A^{2}*Var\left( G \right) \right]}{G^{4}}+\frac{\left[ Var\left( G \right) \right]}{G^{2}}$$

Where: $G=\left\{ \frac{1}{L}\sum_{i=1}^{L} {}^{2}H_{\gamma} \right\}and A=\left\{ \frac{1}{L}\sum_{i=1}^{L} \overline{{}^{2}H_{\alpha}} \right\}$

D-measures

$${Var(}^{q}D_{\beta.A.AvFirst})=\frac{\left[ Var\left( G \right) \right]}{A^{2}}+\frac{\left[ G^{2}*Var\left( A \right) \right]}{A^{4}}$$

Where: $G= \left\{ \frac{1}{L}\sum_{i=1}^{L} {}^{q}D_{\gamma} \right\} and A= \left\{ \frac{1}{L}\sum_{i=1}^{L} \overline{{}^{q}D_{\alpha}} \right\}$
