# Supplementary material for: Detecting steps in spatial genetic data: Which diversity measures are best?
Source: PLoS One. 2022 Mar 14;17(3):e0265110. doi: 10.1371/journal.pone.0265110 (PMC8920294; doi:10.1371/journal.pone.0265110)
Supplement: S2 File — Figure S2.1—Graphical representation of step detection protocol. Figure S2.2—Dependence of alpha diversity on allele proportion. Figure S2.3—Effect of allele proportion on q = 0 measures. (DOCX) [file pone.0265110.s002.docx]

**Supplemental information S3**. **Extra figures**


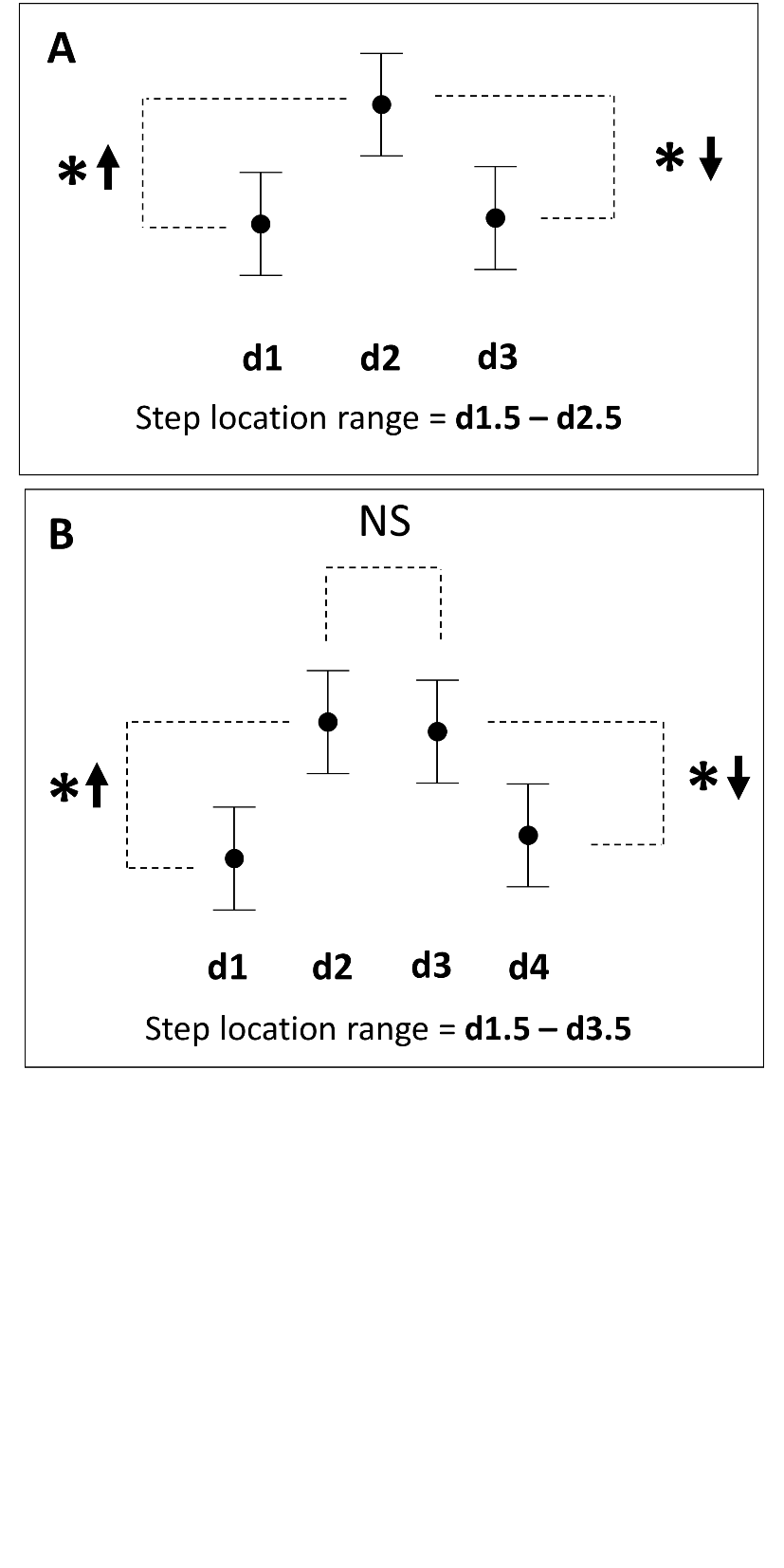


**Figure S2.1**. **Graphical representation of step detection protocol.** Both criteria (A and B) for detecting a step in beta diversity. Each point represents each consecutive average beta diversity along a distance (d1, d2 etc), calculated between localities either side (d0.5, d1.5, etc.). Error bars represent the standard error of the beta diversity measurement. A step is counted as present if: A - the beta diversity between two adjacent localities was significantly higher (less than p = 0.05 in a t-test) than both the beta diversity of the previous adjacent locality pair and the beta diversity of the next adjacent locality pair; or B - the beta diversities between the two adjacent pairs of localities were not significantly different, but the beta diversity of that pair of localities was significantly higher than the beta diversity of the previous and next adjacent locality pair. Stars denote a significant difference, the arrows denote the direction of difference and “NS” represents no significant difference. When a step was detected its location was recorded as: A - the range between the localities of the highest beta (d1.5-2.5); or B - the range between the furthest localities of the highest two betas (d1.5-3.5).


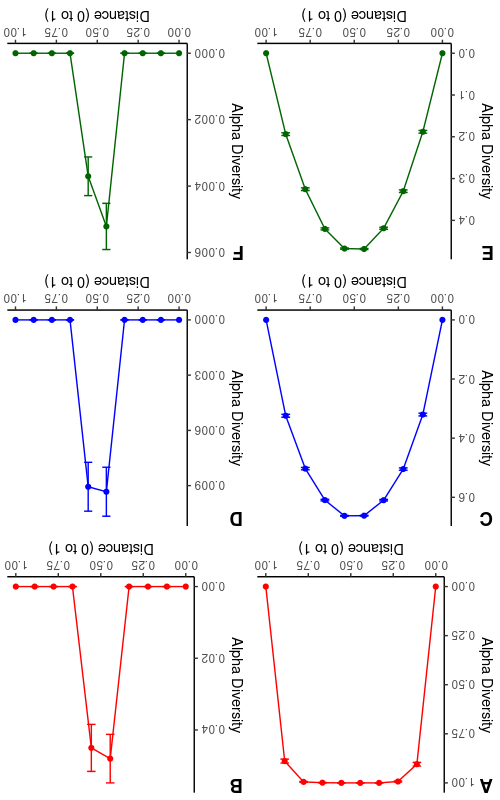


**Figure S2.2**. **Dependence of alpha diversity on allele proportion.** Alpha diversity measures (q = 0 – red, A + B; q = 1 – blue, C + D; q = 2 – green, E + F) were ineffective at detecting steps due to their high dependence of allele proportion regardless of the intensity of the step (linear gradient – A, C and E; large step – B, D and F). All plots use simulations with p = 0 at d = 0 to p = 1 at d = 1 (where p is allele proportion and d is distance).


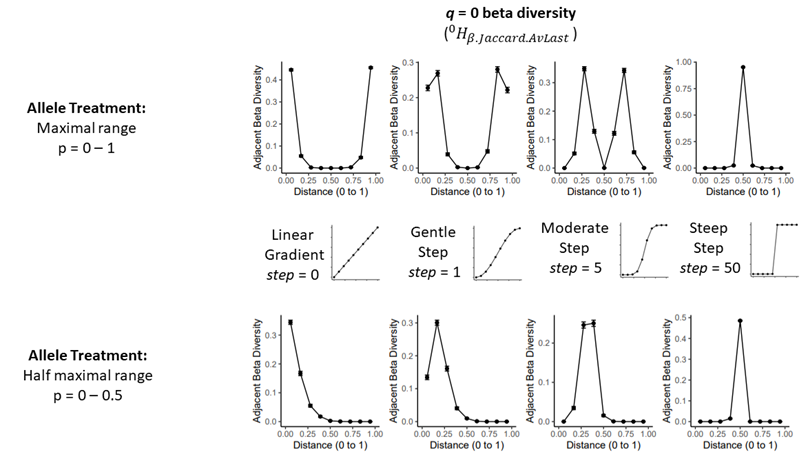


**Figure S2.3**. **Effect of allele proportion on *q* = 0 measures.** Adjacent beta diversities of *q* = 0 measures across the four step intensities (middle row). For the maximal range treatment (top row) there were often two peaks of beta diversity at intermediate step intensities, while in the half maximal range treatment (bottom row) there was only one. These peaks in *q* = 0 beta appear indicate a departure from fixation (*p* = 0 or 1) rather than the presence of a step, making these measures not suitable for step detection.
