# Supplementary material for: Detecting steps in spatial genetic data: Which diversity measures are best?
Source: PLoS One. 2022 Mar 14;17(3):e0265110. doi: 10.1371/journal.pone.0265110 (PMC8920294; doi:10.1371/journal.pone.0265110)
Supplement: S3 File — (DOCX) [file pone.0265110.s003.docx]

Supplemental Information S3 - Expanded results: Detailed comparison of the six best candidate measures under suboptimal conditions

Here we more closely examine the properties of the six candidate measures that had the best properties under standard conditions (${}^{1}H_{\beta.MI.AvLast}$, ${}^{1}H_{\beta.MI.AvFirst}$, ${}^{2}H_{\beta.GST.AvLast}, {}^{2}H_{\beta.GST.AvFirst}$, ${}^{2}D_{\beta.A.AvFirst}$, and Bray-Curtis). We examine how the measures' step sensitivities vary for each of the five allele proportion treatments. In each treatment, we examine the properties of each measure in a single simulation under standard conditions to describe its relationship with allele position as well as its standard error properties. Also in each treatment, we describe how varying sample size of genomes, number of loci sampled and number of localities change the step sensitivities of each measure. We include a figure for each of the six candidate measures for each allele treatment highlighting these properties at the end of this supplement (Figures S3.6.1 to S3.6.30). An index for all figures is included on page 199.

The format for each figure in this supplement is the same, but with different measures and different allele treatments (marked at the top of each figure). Top row: Step intensity treatments used in simulations (Linear - step = 0, gentle step – step = 1, moderate step – step = 5, steep step – step = 50). Each graph in the rows below corresponds to the step treatment in that column. E.g. the first column represents simulations for the linear treatments. Second row: A single example simulation with the number of samples/loci/localities indicated by the dotted vertical line the relevant row below (*n* = 20, *L* = 1000, *K* = 10). Error bars represent the standard error of each measure. Third, Fourth and Bottom Rows: Step detection sensitivity of diversity measures with increasing number of samples (*n,* third row), number of loci (*L,* fourth row). and number of localities (*K,* bottom row). Solid lines represent number of true positives detected (out of 100), dashed lines represent *total* number of steps detected therefore show the number of false positives and true positives detected (the difference between the dashed and solid lines is the number of false positives).

### S3.1 Maximal Range - p = 0 to p = 1

The sample simulations show that the beta measures Bray-Curtis and ${}^{2}D_{\beta.A.AvFirst}$have a convex (with an intermediate maximum) relationship to allele proportion even when a step is not present, with the highest beta diversity at *p* = 0.5 (Figures S3.6.21, S3.6.26, Row 2, Column 1). This property explains why both measures are prone to detecting false steps, which occurs more frequently when the number of localities is smaller (Figures S3.6.21, S3.6.26, Row 5, Column 1). In comparison, the other candidate measures have a slightly concave relationship with the linear gradient of allele proportion, only becoming convex when a step is present (Figures S3,6.1, S3.6.6, S3.6.11, S3.6.16). This property is better for detecting steps over large allele proportion gradients. Each of the candidate measures have strong peaks of adjacent beta diversity when the step is steep, and smaller peaks of diversity when the step is gentle. Noticeably, ${}^{2}H_{\beta.GST.AvFirst}$ had a much higher standard error than all other measures (Figure S3.6.16).

Number of genomes sampled (*n*) did not have much effect in the maximal range allele treatment. In the steep and moderate step treatments, all measures could detect steps 100% of the time, regardless of sample size. The exception to this is ${}^{2}H_{\beta.GST.AvFirst}$ which can only detect a step above sample size *n* = 5-10 (Figure S3.6.16, Row 3, Columns 4-5). In the linear treatment, there is a range of sample sizes where ${}^{1}H_{\beta.MI.AvLast}$ often detects a false step (Figure S3.6.1, Row 3, Column 1, dashed line). There exists a similar, but smaller, peak of false steps for ${}^{2}H_{\beta.GST.AvLast}$ in the linear gradient treatment (Figure S3.6.11). Further, for the gentle step treatment, when the genome sample size is greater than 5, ${}^{1}H_{\beta.MI.AvLast}$ detects a step at the incorrect location about 25% of the time (Figure S3.6.1). By examining the example simulation in the second row of the plot, these false steps appear to be located near fixation (not unlike the peaks detected by *q* = 0 measures).

The effect of increasing the number of loci sampled (*L*) was similar to increasing the number of genomes sampled. However, in the gentle step treatment Bray-Curtis and ${}^{2}D_{\beta.A.AvFirst}$ had increasing step sensitivity with an increasing number of loci (Figures S3.6.26, Figure S3.6.21). Also in the gentle step treatment, the number of false steps for ${}^{1}H_{\beta.MI.AvLast}$ and ${}^{2}H_{\beta.GST.AvLast}$ were largely constant (Figures S3.6.1, Figure S3.6.11), indicating that the number of loci does not have as much an effect compared to the number of genomes sampled. As with number of genomes, ${}^{2}H_{\beta.GST.AvFirst}$ had a threshold of number of loci needed before it could detect a steep or moderate step 100% of the time (~400 loci; Figure S3.6.16). For the moderate step, ${}^{1}H_{\beta.MI.AvFirst}$ and ${}^{1}H_{\beta.MI.AvLast}$ also had thresholds before detecting a step (~300 and ~200 loci respectively; Figures S3.6.6, S3.6.1). These thresholds distinguished these measures from the other three which detected a step 100% of the time regardless of the number of loci sampled.

When the number of localities sampled (*K*) was low, the chance of detecting a step increased. As with the number of loci sampled, the moderate step treatment distinguished ${}^{2}H_{\beta.GST.AvFirst}$, followed by ${}^{1}H_{\beta.MI.AvFirst}$ then ${}^{1}H_{\beta.MI.AvLast}$ as having slightly lower step detection sensitivities than the other candidate measures. In the steep step treatment ${}^{2}H_{\beta.GST.AvFirst}$ had wildly inconsistent step detection properties with increasing numbers of localities (Figure S3.6.16, Row 5, Column 5). This may be due to the measure's high standard error and indicates that it might not be a suitable step detection measure. There were other effects of changing the number of localities sampled. In the linear gradient, when the number of localities sampled decreased, ${}^{2}D_{\beta.A.AvFirst}$was increasing likely to detect a false step (Figure S3.6.21, Row 5, column 1). Bray-Curtis also was more likely to detect a false step as localities decreased to six but then was less likely to detect false steps when localities were less than six (Figure S3.6.26), and the false detection rate was never more than 25%. In the gentle step treatment, the relative sensitivities of the measures can be separated with ${}^{1}H_{\beta.MI.AvFirst}$, ${}^{1}H_{\beta.MI.AvLast}$ and ${}^{2}H_{\beta.GST.AvFirst}$ being least sensitive, followed by ${}^{2}H_{\beta.GST.AvLast}$, then ${}^{2}D_{\beta.A.AvFirst}$ and Bray-Curtis with the highest sensitivity. When number of localities increases from six in the slight step treatment, ${}^{1}H_{\beta.MI.AvLast}$ detects false step at an increasing frequency (from about 30% to 45% at 15 localities; Figure S3.6.1).

### S3.2 Maximal range without fixation - p = 0.1 to p = 0.9

The sample simulations (Row 2, Figures S3.6.22, S3.6.27) show the same convex relationship of Bray-Curtis and ${}^{2}D_{\beta.A.AvFirst}$ beta measures and allele proportion, even when there is no step. Both the pairs of *G_ST_* measures and *q* = 1 measures have similar beta values across simulations, with the *AvFirst* variants of each having higher standard errors and standard errors lowest near *p* = 0.5 (Figures S3.6.2, S3.6.7, S3.6.12, S3.6.17). The standard errors of ${}^{2}H_{\beta.GST.AvFirst}$are still highest, but not tosuch an extent as in the maximal range treatment.

The number of genomes sampled (*n*) had similar relationships with beta diversity as in the *maximal range* treatments, but with lower false detection rates and lower step detection sensitivity for Bray-Curtis and ${}^{2}D_{\beta.A.AvFirst}$ (Figures S3.6.27, S3.6.22). The peak of false detections for ${}^{1}H_{\beta.MI.AvLast}$ and ${}^{2}H_{\beta.GST.AvLast}$ in the *maximal range* treatment was not present in the *maximal range without fixation* treatment, with minimal false detection when *n* < 10 (Figures S3.6.2, S3.6.12). Rates of incorrect step detection in the gentle step treatment were also greatly reduced. The step sensitivity of Bray-Curtis and ${}^{2}D_{\beta.A.AvFirst}$was reduced by about half. A steep step was detected 100% of the time by all measures, as was a moderate step (with the exception of ${}^{2}H_{\beta.GST.AvFirst}$, which could detect the moderate step above *n* = 10; Figure S3.6.17).

The effect of an increasing number of loci (*L*) was similar to that of the maximal range treatment, but with a lower rate of false positives for Bray-Curtis and fewer incorrect steps for ${}^{1}H_{\beta.MI.AvLast}$ and ${}^{2}H_{\beta.GST.AvLast}$. As with the maximal range treatment, Bray-Curtis and ${}^{2}D_{\beta.A.AvFirst}$ were more sensitive to the gentle step with increasing numbers of loci, but took more loci to reach the same step detection percentage (Figures S3.6.27, S3.6.22). As with the number of samples, all measures detected a steep step 100% of the time regardless of the number of loci sampled. Most measures could detect the moderate step regardless of the number of loci, except ${}^{1}H_{\beta.MI.AvFirst}$ and ${}^{2}H_{\beta.GST.AvFirst}$ which could only detect such a step consistently after the number of loci was about 400 (Figures S3.6.7, S3.6.17).

Varying the number of localities (*K*) showed similar patterns to the maximal range treatment, but without the inconsistent behaviour of ${}^{2}H_{\beta.GST.AvFirst}$ in the steep step treatment. When the step was steep all measures could detect a step 100% of the time regardless of the number of localities sampled. This was the same for a moderate step except for ${}^{2}H_{\beta.GST.AvFirst}$ and ${}^{1}H_{\beta.MI.AvFirst}$ which had reduced step detection sensitivity when the number of localities was higher than thirteen (Figures S3.6.7, S3.6.17). When the step was gentle, the measures could be more distinguished, with ${}^{1}H_{\beta.MI.AvFirst}$ and ${}^{2}H_{\beta.GST.AvFirst}$ being least sensitive, followed by ${}^{1}H_{\beta.MI.AvLast}$, then ${}^{2}H_{\beta.GST.AvLast}$, then ${}^{2}D_{\beta.A.AvFirst}$, and lastly Bray-Curtis with the highest sensitivity. For lower number of localities, as with the maximal range treatment, ${}^{2}D_{\beta.A.AvFirst}$ detected large number of false positives and Bray-Cutis detected an intermediate rate of false positives (Figures S3.6.22, S3.6.27).

### S3.3 Half-maximal range - p = 0 to p = 0.5

The sample simulations with the linear treatment revealed that Bray-Curtis and ${}^{2}D_{\beta.A.AvFirst}$ increased with increasing values of *p* (Column 1, Row 2, Figures S3.6.28, S3.6.23). In contrast, the *q* =1 and *G_ST_* measures were largely flat (Column 1, Row 2, Figures S3.6.3, S3.6.8, S3.6.13, S3.6.18). In the gentle step treatment, ${}^{1}H_{\beta.MI.AvLast}$, ${}^{2}H_{\beta.GST.AvLast}$ and Bray-Curtis showed peaks of beta diversity not at the location of step. The Bray-Curtis measure had a peak of diversity towards *p* = 0.5 (Column 2, Row 2, S3.6.28), whereas ${}^{1}H_{\beta.MI.AvLast}$ and ${}^{2}H_{\beta.GST.AvLast}$ had peaks closer to *p* = 0 (fixation; Column 2, Row 2, S3.6.3, S3.6.13).

By varying the number of genomes sampled (*n*) we could more clearly see the false step and incorrect step issues of ${}^{1}H_{\beta.MI.AvLast}$ and ${}^{2}H_{\beta.GST.AvLast}$ (Row 3, S3.6.3, S3.6.13). For the linear step treatment, between sample sizes of 5 and 22 ${}^{1}H_{\beta.MI.AvLast}$ has a peak of false step detections (up to 50% of simulations). Similarly, between sample sizes of 10 and 25, ${}^{2}H_{\beta.GST.AvLast}$ has a smaller peak of false step detections (up to 20% of simulations). In the gentle step treatment ${}^{1}H_{\beta.MI.AvLast}$ detected steps at the incorrect location with increasing frequency until the sample size was 20, where it decreased slightly. ${}^{2}H_{\beta.GST.AvLast}$ and Bray-Curtis also detected a step at the incorrect location with increasing *n*, but at a lower frequency. While all measures detected a steep step 100% of the time regardless of the number of genomes sampled, varying *n* in the moderate step treatment separated the relative sensitivities of the measures. The most sensitive measure was ${}^{1}H_{\beta.MI.AvLast}$, closely followed by ${}^{2}H_{\beta.GST.AvLast}$, ${}^{1}H_{\beta.MI.AvLast}$ and Bray-Curtis, then ${}^{2}D_{\beta.A.AvFirst}$. ${}^{2}H_{\beta.GST.AvFirst}$ could not detect a moderate step regardless of the number of genomes sampled (Figure S3.6.18).

With an increasing number of loci (*L*), the rate of false positives was largely consistent for ${}^{1}H_{\beta.MI.AvLast}$ and ${}^{2}H_{\beta.GST.AvLast}$ (Figures S3.6.3, S3.6.13), as was the rate of incorrect steps in the gentle step treatment for ${}^{2}H_{\beta.GST.AvLast}$ and Bray-Curtis (Figures S3.6.13, S3.6.28). The rate of incorrect steps for ${}^{1}H_{\beta.MI.AvLast}$ increased with increasing number of loci (Row 4, Figure S3.6.3). The steep step was detected by all measures for all values of *L*. The moderate step was detected for most values of *L* for ${}^{1}H_{\beta.MI.AvLast}$, ${}^{2}H_{\beta.GST.AvLast}$ and Bray-Curtis. However, the moderate step could only be consistently detected by ${}^{1}H_{\beta.MI.AvFirst}$ above 300 loci, by ${}^{2}D_{\beta.A.AvFirst}$ above 700, and not at all by ${}^{2}H_{\beta.GST.AvFirst}$ (Figures S3.6.8, S3.6.23, S3.6.18).

The number of localities sampled (*K*) had different effects on each of the candidate measures. While all measures detected a steep step for all simulated vales of *K*, the moderate step treatment separates their sensitivities. For values of *K* less than 14, ${}^{2}H_{\beta.GST.AvLast}$ and Bray-Curtis can detect the moderate step consistently (Figures S3.6.13, S3.6.28). This is also the case for ${}^{1}H_{\beta.MI.AvLast}$, but when the localities were 11 or 13 this measure sometimes detected the step at the incorrect location (Figure S3.6.3). ${}^{1}H_{\beta.MI.AvFirst}$had a similar inconsistent behaviour but with a slightly lower true positive detection rate more similar to ${}^{2}D_{\beta.A.AvFirst}$ (Figures S3.6.8, S3.6.23). ${}^{2}H_{\beta.GST.AvFirst}$ was much less sensitive than other measures overall, detecting the moderate step 100% of the time when *K* was 4 or 6, but not all for all other values of *K* (Figure S3.6.18). Further, ${}^{2}H_{\beta.GST.AvFirst}$ could not detect the gentle step at all, while the other measures could detect it at low values of *K*. The gentle step treatment revealed Bray-Curtis and ${}^{2}H_{\beta.GST.AvLast}$ to be the most sensitive with small rates of incorrect step detection. ${}^{1}H_{\beta.MI.AvLast}$ had a higher rate of step detection in this treatment but most of this detection was incorrect, with correct step detection rate more similar to ${}^{1}H_{\beta.MI.AvFirst}$ and ${}^{2}D_{\beta.A.AvFirst}$. In the linear gradient, ${}^{1}H_{\beta.MI.AvLast}$ had a peak of false detection (up to 50%) and ${}^{2}H_{\beta.GST.AvLast}$ had a smaller peak of false step detection (15%).

### S3.4 Narrow range close to fixation - p = 0 to p = 0.2

The example simulations revealed how each of the measures behaved close to fixation both in terms of trends of means but also standard errors. This was especially noticeable in the linear treatment where no measure had a flat distribution of betas. As with the other treatments Bray-Curtis and ${}^{2}D_{\beta.A.AvFirst}$ adjacent beta diversities increased with increasing allele proportion, and their standard errors also increased (Figures S3.6.29, S3.6.24). Both variants of the *q* =1 measures were consistent across the range except for very close to fixation where beta diversities and standard error was slightly lower (${}^{1}H_{\beta.MI.AvFirst}$ had a consistently higher standard error than ${}^{1}H_{\beta.MI.AvLast}$, but the same means; Figures S3.6.4, S3.6.9). ${}^{2}H_{\beta.GST.AvLast}$ showed a similar pattern to the *q* = 1 measures (Figure S3.6.14). Whereas ${}^{2}H_{\beta.GST.AvFirst}$ instead increased close to fixation and also had considerably higher standard errors. These high standard errors were highest close to, but not at, fixation, as seen in moderate and steep step treatments (S3.6.19).

Varying the number of genomes (*n*) revealed the comparative sensitivities of the measures. While there was a lower rate of false positives in the linear step treatment for ${}^{2}H_{\beta.GST.AvLast}$and ${}^{1}H_{\beta.MI.AvLast}$ compared to other allele treatments, both measures had an increased rate of false positives at higher values of *n* (Figures S3.6.4 and S3.6.14). This was similar to the false positive rate in the gentle step treatment, which increased with higher values of *n*. The moderate step treatment showed that ${}^{1}H_{\beta.MI.AvLast}$ had the highest true positive rate closely followed by ${}^{2}H_{\beta.GST.AvLast}$. Also in the moderate step treatment, Bray-Curtis could only detect the moderate step after *n* = 20, and even then it seemed that *n* would have to be much higher for 100% detection (Figure S3.6.29). The other measures could not detect a moderate step under any tested value of *n* (Figures S3.6.9, S3.6.19, S3.6.24). Most measures could detect a steep step 100% after *n* = 5, except ${}^{2}D_{\beta.A.AvFirst}$ which could only consistently detect the steep step after *n* = 15. Interestingly, ${}^{2}H_{\beta.GST.AvFirst}$ has a slightly better step detection sensitivity than other measures despite its poor performance detecting steps in the moderate step treatment (figure S3.6.19).

The effect of the number of loci (*L*) was similar to that of *n*. In the moderate step treatment ${}^{2}H_{\beta.GST.AvLast}$ and ${}^{1}H_{\beta.MI.AvLast}$ performed best being able to detect a step 100% of the time with above 300-500 loci (Figures S3.6.4, S3.6.14). In comparison, the other measures could not consistently detect the moderate step with any of the tested loci treatments. In the steep step treatment, all measures could detect a step 100% of the time for all tested *L*, except for ${}^{2}H_{\beta.GST.AvFirst}$ which could detect he step above 200 loci (Figure S3.6.19) and ${}^{2}D_{\beta.A.AvFirst}$ detecting steps above 700 loci (Figure S3.6.24). In the gentle step treatment no measure could consistently detect a step, but ${}^{1}H_{\beta.MI.AvLast}$ detected a step at a higher rate as the number of loci increased (Figure S3.6.4).

Varying the number of localities (*K*) separated the step detection properties of the measures. In the linear gradient treatment there were peaks of false step detection for ${}^{1}H_{\beta.MI.AvLast}$ and ${}^{2}H_{\beta.GST.AvLast}$ for low values of *K* (Figures S3.6.4, S3.6.14), but no false step detection for any of the other measures. The gentle step treatment revealed that ${}^{1}H_{\beta.MI.AvLast}$ was the most sensitive followed by ${}^{2}H_{\beta.GST.AvLast}$, then Bray-Curtis then ${}^{1}H_{\beta.MI.AvFirst}$. ${}^{2}H_{\beta.GST.AvFirst}$ and ${}^{2}D_{\beta.A.AvFirst}$ could not detect a step in the gentle step treatment (Figures S3.6.14, S3.6.24). The moderate step treatment showed a similar pattern but with higher rates of step detection and ${}^{2}D_{\beta.A.AvFirst}$ sometimes detecting a step when *K* = 4 (Figure S3.6.24). ${}^{1}H_{\beta.MI.AvFirst}$ had inconsistent behaviour in the moderate and steep step treatments depending on the evenness of the number of localities (Figure S3.6.9). This inconsistent property was even more pronounced for ${}^{2}H_{\beta.GST.AvFirst}$ in steep step treatment, alternating between 0% and 100% step detection (Figure S3.6.9). All other measures could detect the steep step 100% of the time for the steep step treatment.

### S3.5 Narrow range far from fixation - p = 0.3 to p = 0.5

The example simulations revealed relatively flat beta diversity patterns, even with Bray-Curtis and ${}^{2}D_{\beta.A.AvFirst}$ which did not have such pronounced change with allele proportion (Row 2, Figures S3.6.5, S3.6.10, S3.6.15, S3.6.20, S3.6.25, S3.6.30). Bray-Curtis and ${}^{2}D_{\beta.A.AvFirst}$ also had relatively consistent standard errors with allele proportion, whereas the other measure had decreasing standard errors with higher allele proportions (towards *p* = 0.5).

Regardless of the number of genomes sampled (*n*), all measures could not detect a gentle step. In the moderate step treatment, ${}^{2}H_{\beta.GST.AvLast}$, ${}^{1}H_{\beta.MI.AvLast}$, and Bray-Curtis could detect the step better with increasing *n* and had true positive rates nearly identical to each other (Figures S3.6.5, S3.6.15, S3.6.30). The other measures could not detect a moderate step for any value of *n* (Figures S3.6.10, S3.6.20, S3.6.25). In the steep step treatment, all measures could detect a step after *n* = 5 with ${}^{2}H_{\beta.GST.AvLast}$, ${}^{1}H_{\beta.MI.AvLast}$, and Bray-Curtis having a slightly better sensitivity than the other measures.

The effect of the number of loci (*L*) was near identical to the effect of the number of genomes sampled. In the moderate step treatment, ${}^{2}H_{\beta.GST.AvLast}$, ${}^{1}H_{\beta.MI.AvLast}$, and Bray-Curtis could detect a step more often with increasing loci, and the other measures could not detect the step. In the steep step treatment, ${}^{2}H_{\beta.GST.AvLast}$, ${}^{1}H_{\beta.MI.AvLast}$, and Bray-Curtis could detect a step most of the time with other measures only slightly less sensitive for very low numbers of loci.

Varying the number of localities (*K*) mirrored the results of *n* and *L*, except that low number of localities rather than high number of loci or genomes, was the more favourable condition. ${}^{2}H_{\beta.GST.AvLast}$, ${}^{1}H_{\beta.MI.AvLast}$, and Bray-Curtis could detect a gentle step at near-identical frequencies when the number localities were low (Figures S3.6.5, S3.6.15, S3.6.30). In contrast, ${}^{2}D_{\beta.A.AvFirst}$ could only detect the slight step a small fraction of the time when the number of localities was six and the other measures not at all (Figures S3.6.10, S3.6.20, S3.6.25). Similarly, ${}^{2}H_{\beta.GST.AvLast}$, ${}^{1}H_{\beta.MI.AvLast}$, and Bray-Curtis had the highest step detection sensitivity for the moderate step treatment. The other measures performed worse, with ${}^{2}D_{\beta.A.AvFirst}$ only slightly better than ${}^{2}H_{\beta.GST.AvFirst}$ and ${}^{1}H_{\beta.MI.AvFirst}$. These three measures also had inconsistent behaviour in the steep step treatment with lower sensitivities for odd number treatments. This was likely due to their higher standard errors obscuring the step when the absolute allele proportion differences were smaller when the number of localities was odd.

### S3.6 Individual figures

Below is an index of the figures referenced above, with the format of each described at the start of this supplement.

| **Figure Number** | **Measure** | **Allele Treatment** | **Page Number** |
| --- | --- | --- | --- |
| S3.6.1 | ${}^{1}H_{\beta.MI.AvLast}$ | p = 0 to p = 1 | 13 |
| S3.6.2 | ${}^{1}H_{\beta.MI.AvLast}$ | p = 0.1 to p = 0.9 | 14 |
| S3.6.3 | ${}^{1}H_{\beta.MI.AvLast}$ | p = 0 to p = 0.5 | 15 |
| S3.6.4 | ${}^{1}H_{\beta.MI.AvLast}$ | p = 0 to p = 0.2 | 16 |
| S3.6.5 | ${}^{1}H_{\beta.MI.AvLast}$ | p = 0.3 to p = 0.5 | 17 |
| S3.6.6 | ${}^{1}H_{\beta.MI.AvFirst}$ | p = 0 to p = 1 | 18 |
| S3.6.7 | ${}^{1}H_{\beta.MI.AvFirst}$ | p = 0.1 to p = 0.9 | 19 |
| S3.6.8 | ${}^{1}H_{\beta.MI.AvFirst}$ | p = 0 to p = 0.5 | 20 |
| S3.6.9 | ${}^{1}H_{\beta.MI.AvFirst}$ | p = 0 to p = 0.2 | 21 |
| S3.6.10 | ${}^{1}H_{\beta.MI.AvFirst}$ | p = 0.3 to p = 0.5 | 22 |
| S3.6.11 | ${}^{2}H_{\beta.GST.AvLast}$ | p = 0 to p = 1 | 23 |
| S3.6.12 | ${}^{2}H_{\beta.GST.AvLast}$ | p = 0.1 to p = 0.9 | 24 |
| S3.6.13 | ${}^{2}H_{\beta.GST.AvLast}$ | p = 0 to p = 0.5 | 25 |
| S3.6.14 | ${}^{2}H_{\beta.GST.AvLast}$ | p = 0 to p = 0.2 | 26 |
| S3.6.15 | ${}^{2}H_{\beta.GST.AvLast}$ | p = 0.3 to p = 0.5 | 27 |
| S3.6.16 | ${}^{2}H_{\beta.GST.AvFirst}$ | p = 0 to p = 1 | 28 |
| S3.6.17 | ${}^{2}H_{\beta.GST.AvFirst}$ | p = 0.1 to p = 0.9 | 29 |
| S3.6.18 | ${}^{2}H_{\beta.GST.AvFirst}$ | p = 0 to p = 0.5 | 30 |
| S3.6.19 | ${}^{2}H_{\beta.GST.AvFirst}$ | p = 0 to p = 0.2 | 31 |
| S3.6.20 | ${}^{2}H_{\beta.GST.AvFirst}$ | p = 0.3 to p = 0.5 | 32 |
| S3.6.21 | ${}^{2}D_{\beta.A.AvFirst}$ | p = 0 to p = 1 | 33 |
| S3.6.22 | ${}^{2}D_{\beta.A.AvFirst}$ | p = 0.1 to p = 0.9 | 34 |
| S3.6.23 | ${}^{2}D_{\beta.A.AvFirst}$ | p = 0 to p = 0.5 | 35 |
| S3.6.24 | ${}^{2}D_{\beta.A.AvFirst}$ | p = 0 to p = 0.2 | 36 |
| S3.6.25 | ${}^{2}D_{\beta.A.AvFirst}$ | p = 0.3 to p = 0.5 | 37 |
| S3.6.26 | Bray-Curtis | p = 0 to p = 1 | 38 |
| S3.6.27 | Bray-Curtis | p = 0.1 to p = 0.9 | 39 |
| S3.6.28 | Bray-Curtis | p = 0 to p = 0.5 | 40 |
| S3.6.29 | Bray-Curtis | p = 0 to p = 0.2 | 41 |
| S3.6.30 | Bray-Curtis | p = 0.3 to p = 0.5 | 42 |

**S3.6.1
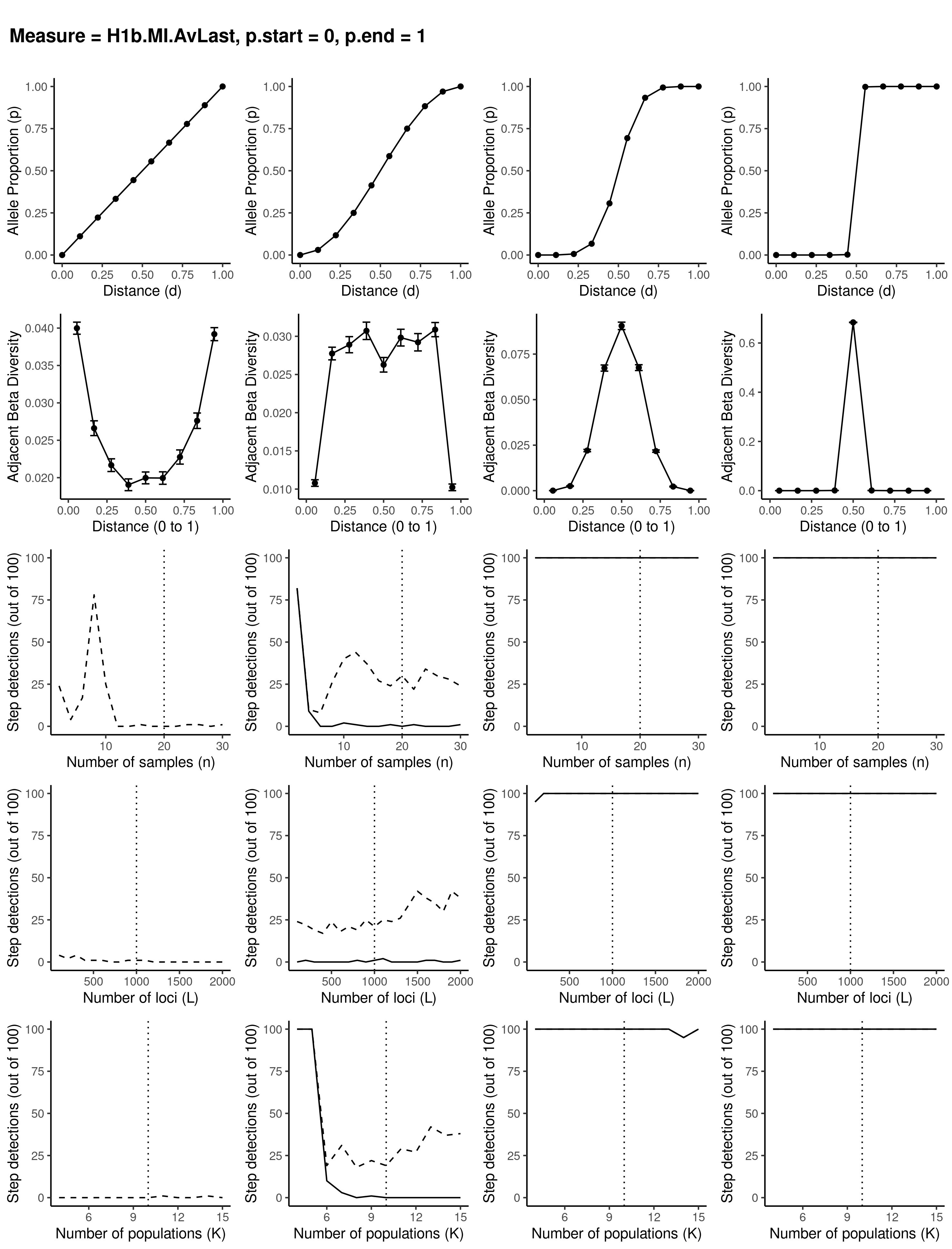
**

**S3.6.2
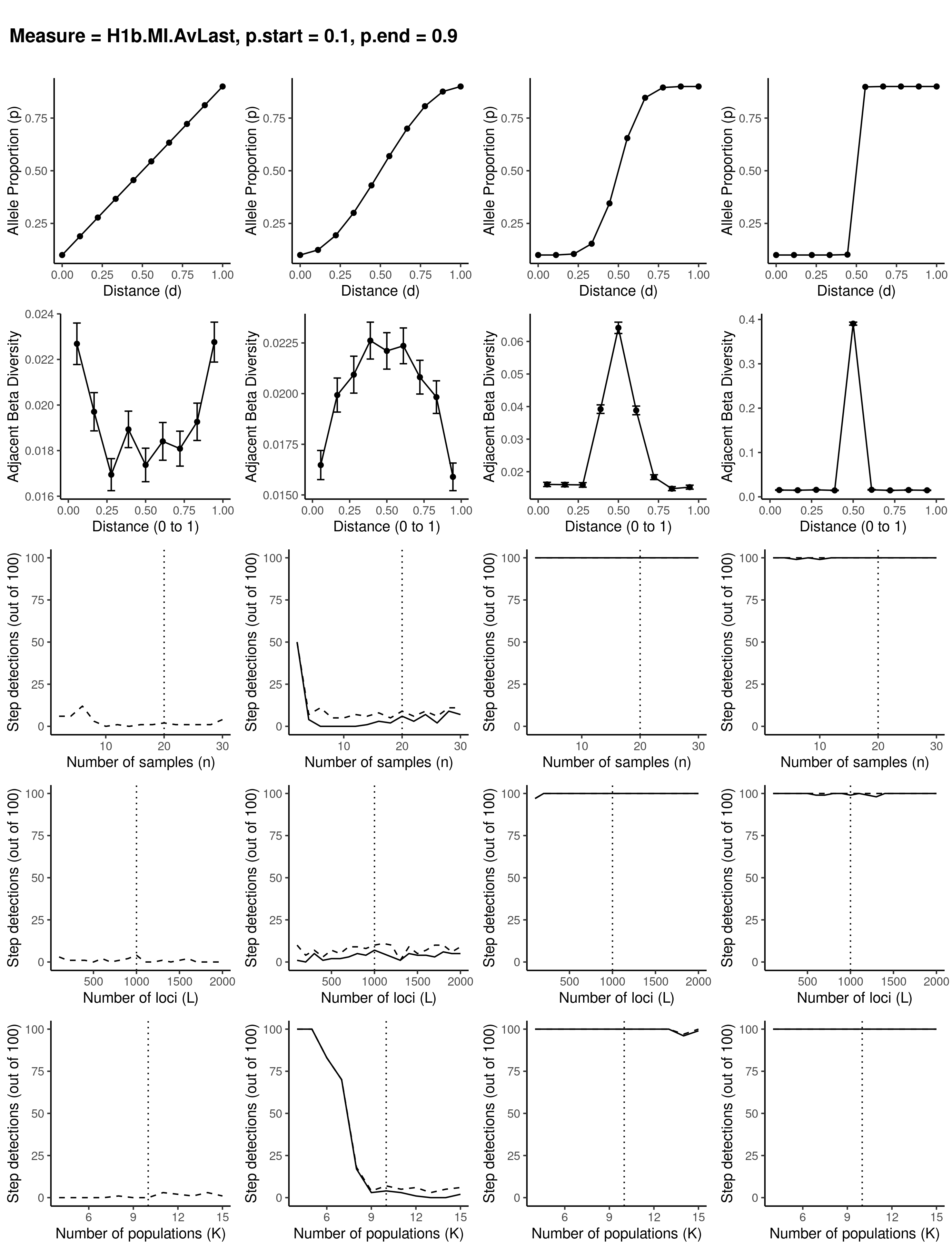
**

**S3.6.3
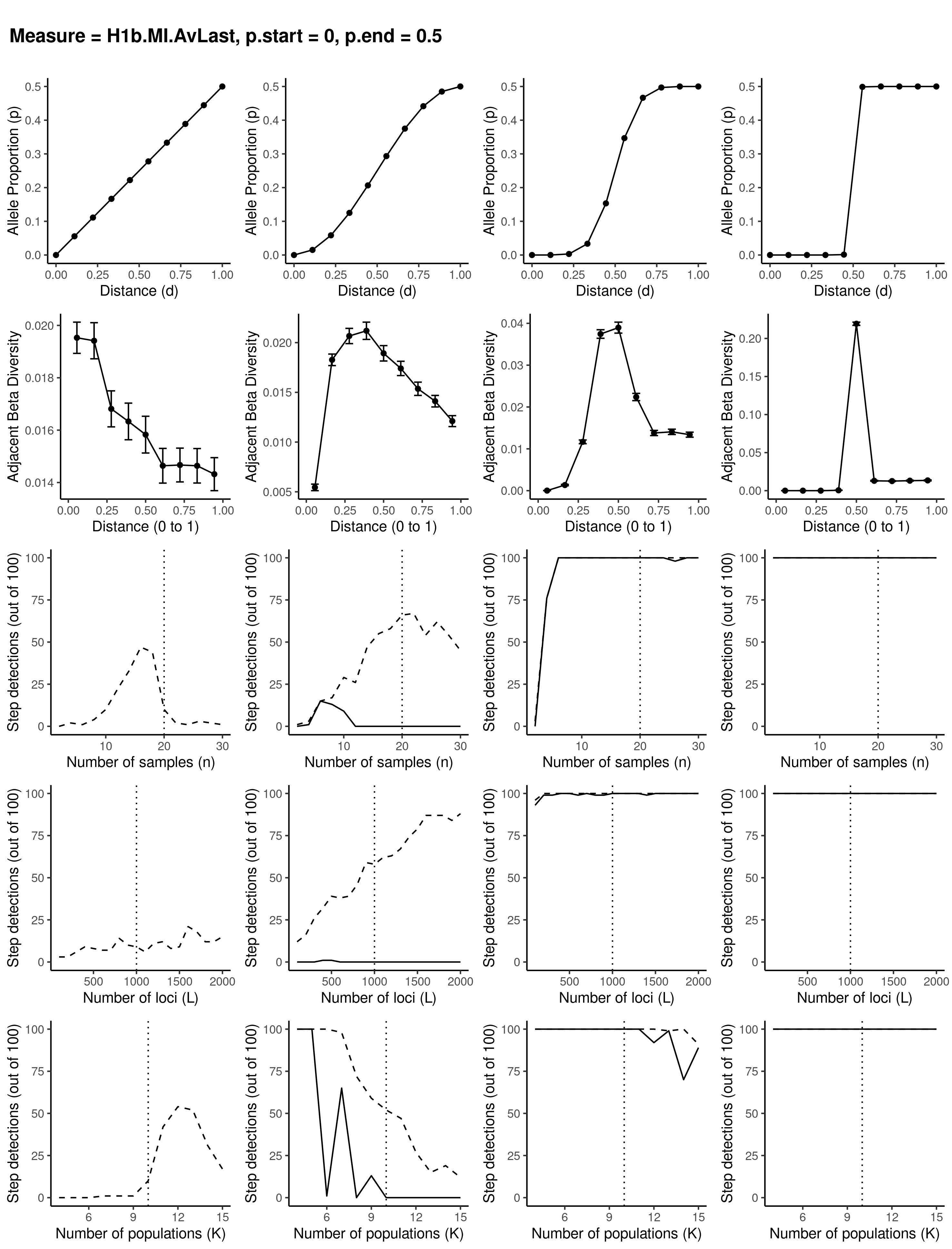
**

**S3.6.4
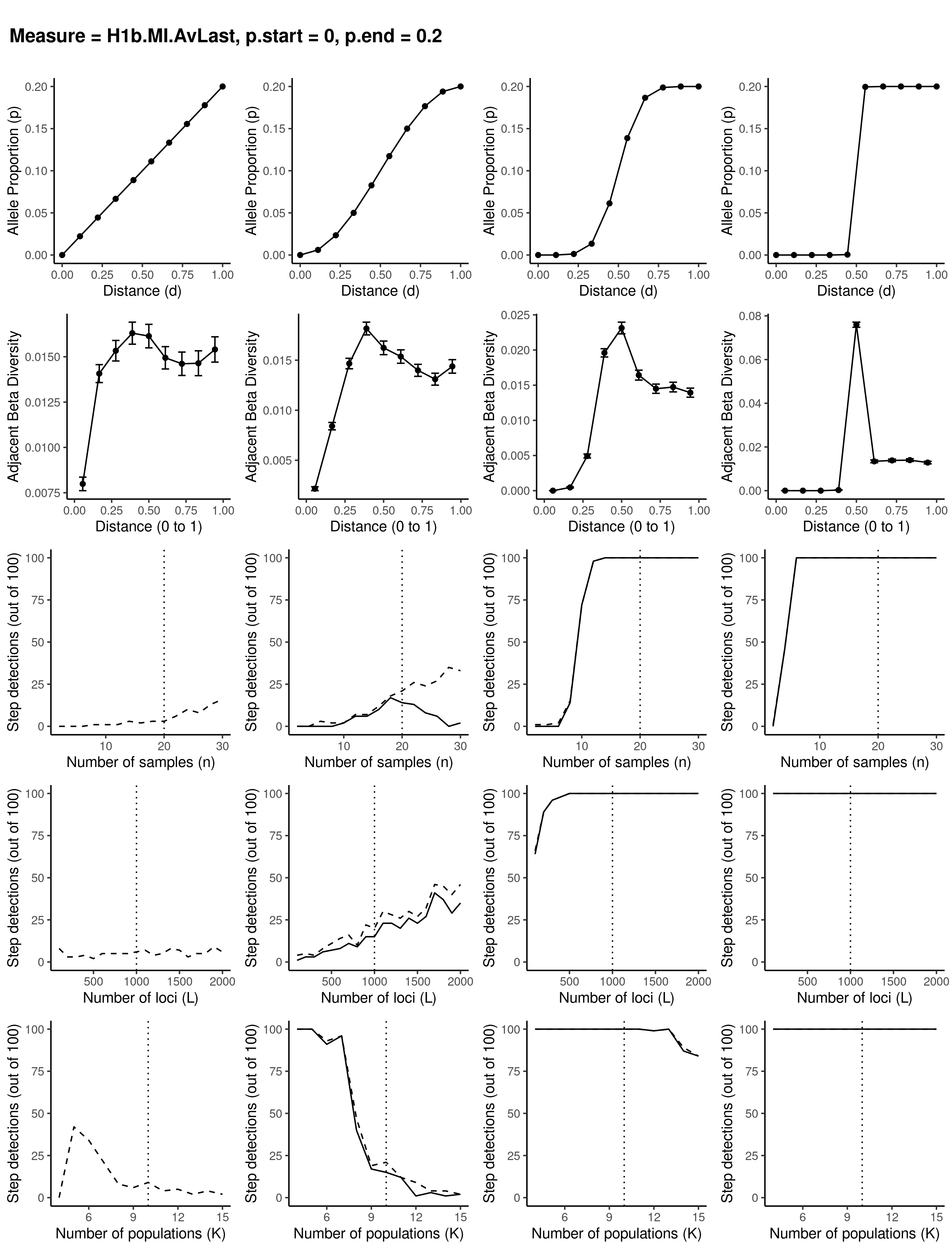
**

**S3.6.5
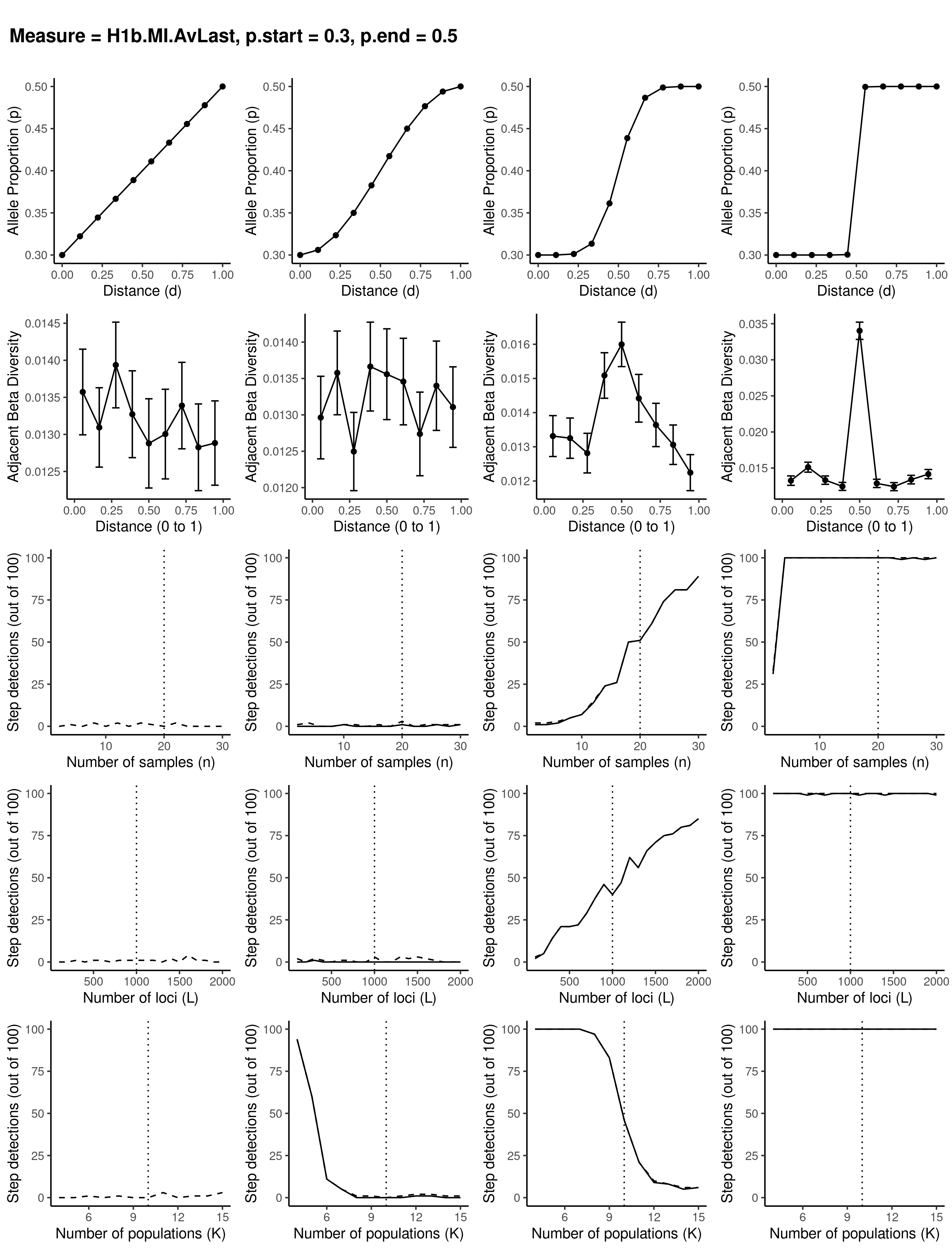
**

**S3.6.6
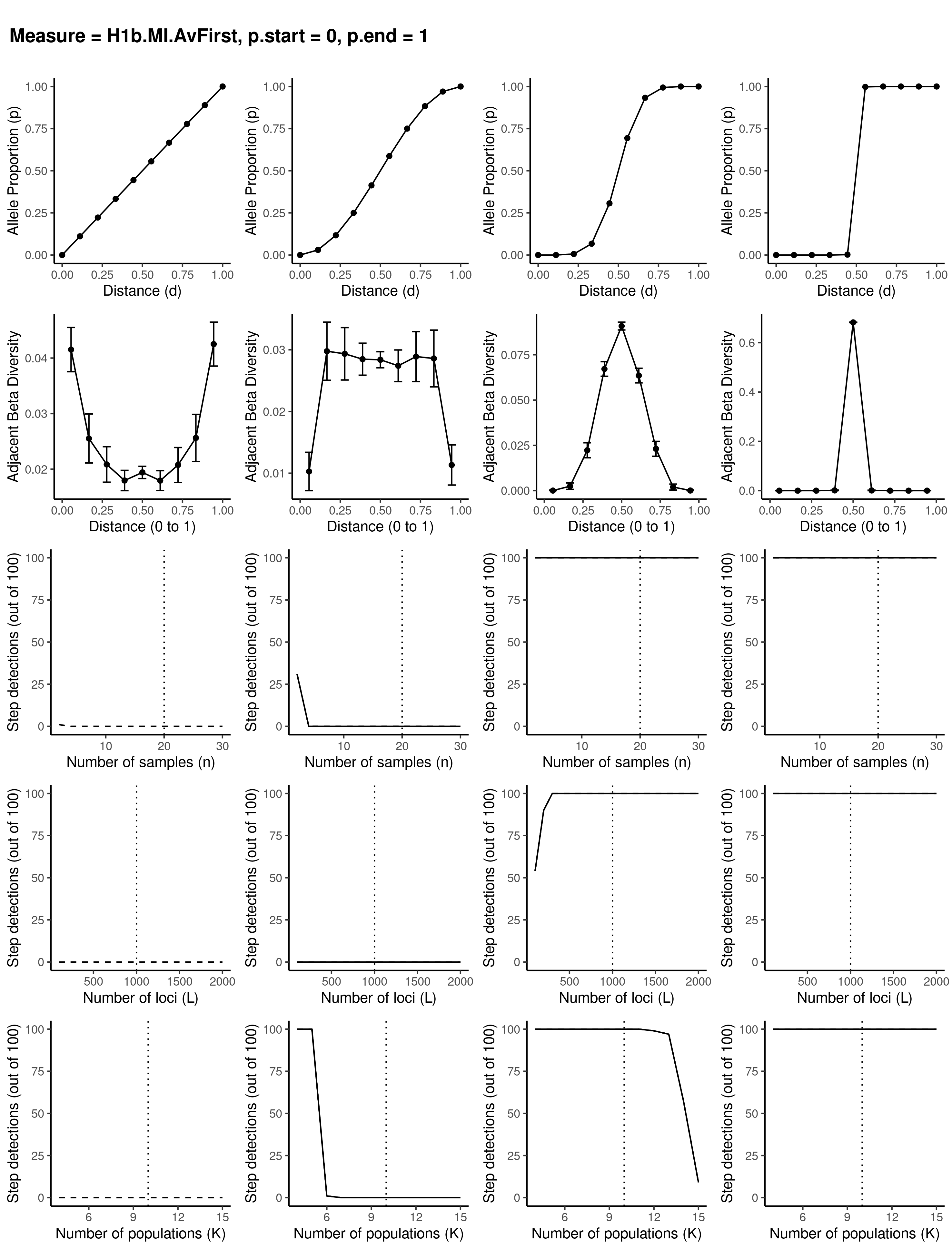
**

**S3.6.7
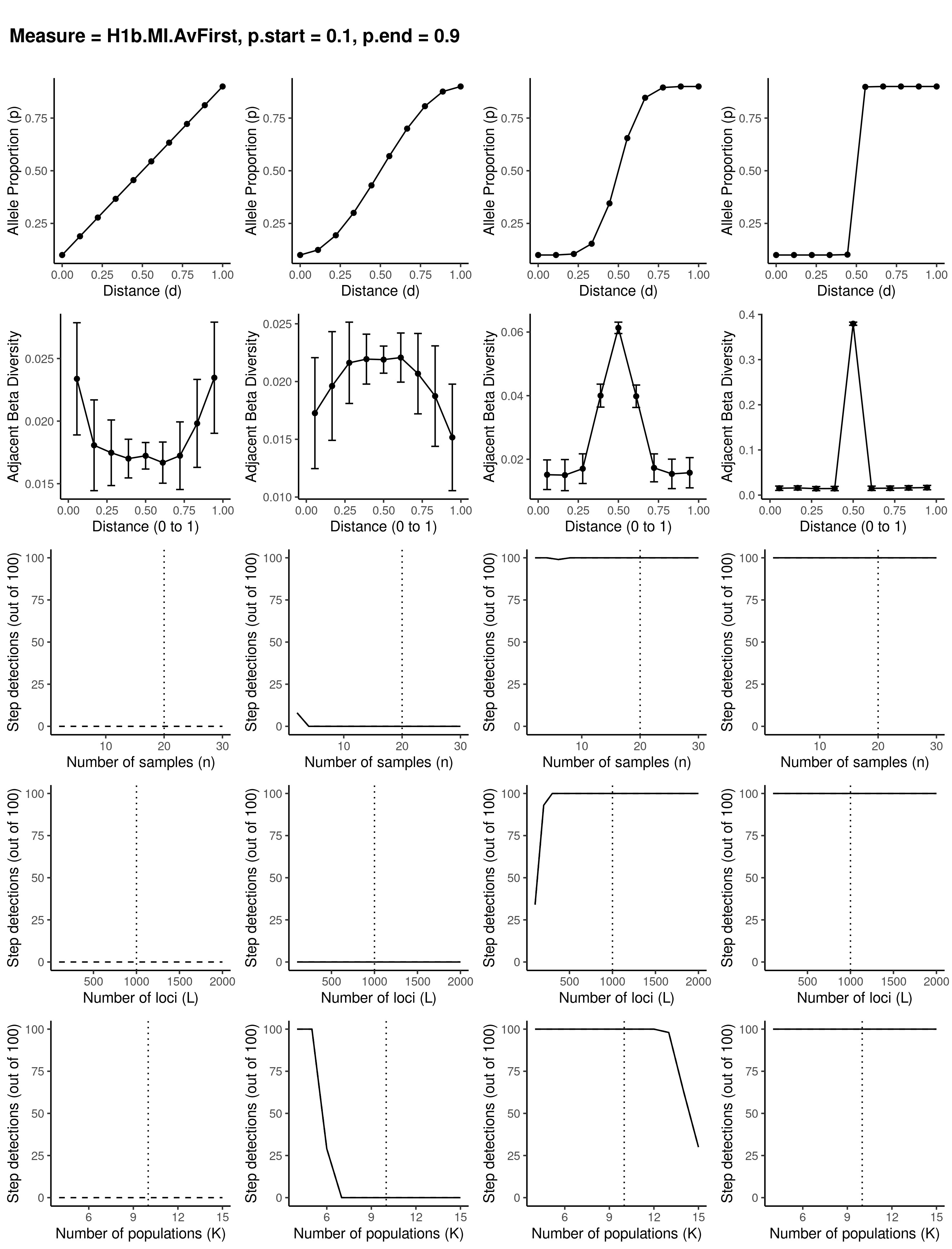
**

**S3.6.8
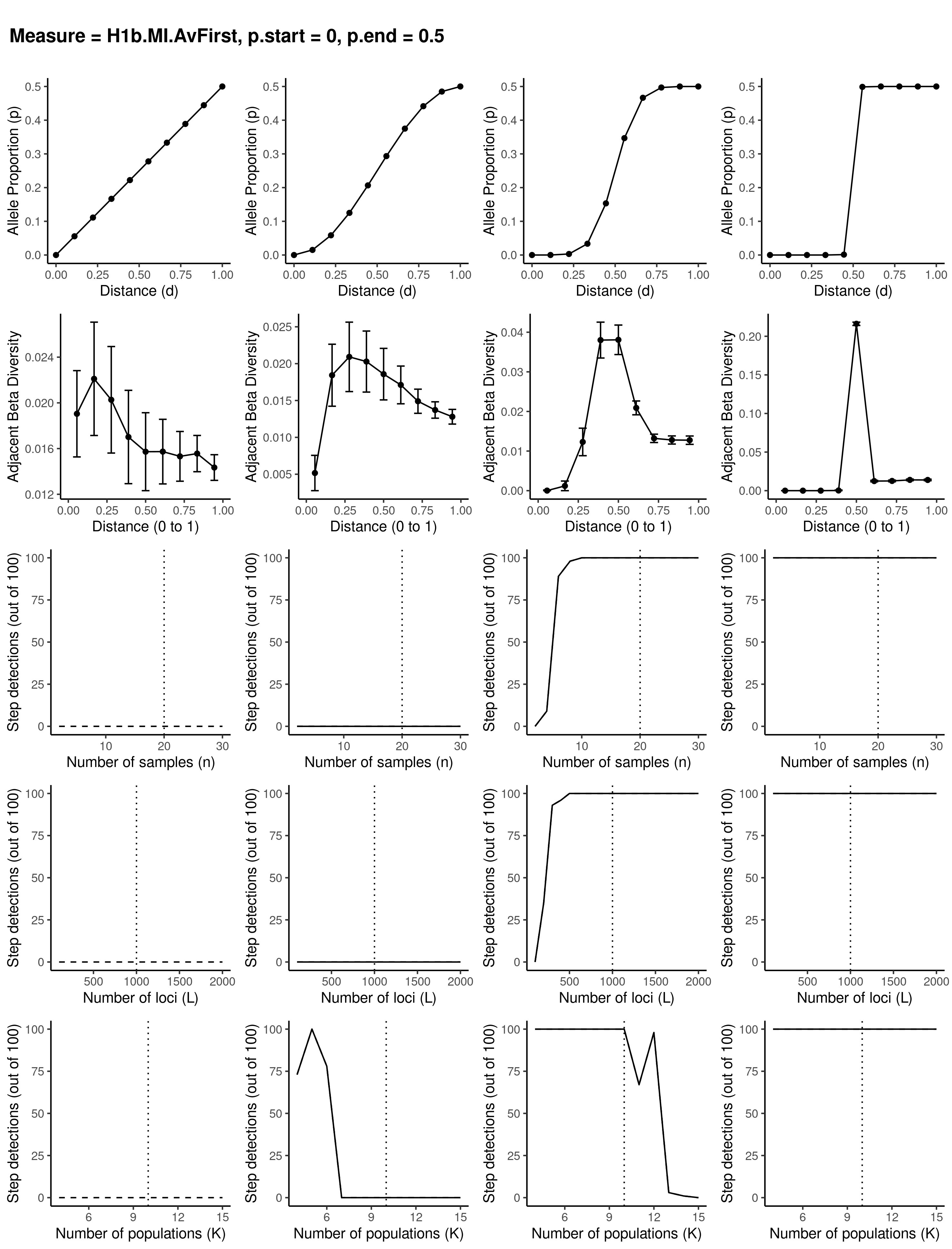
**

**S3.6.9
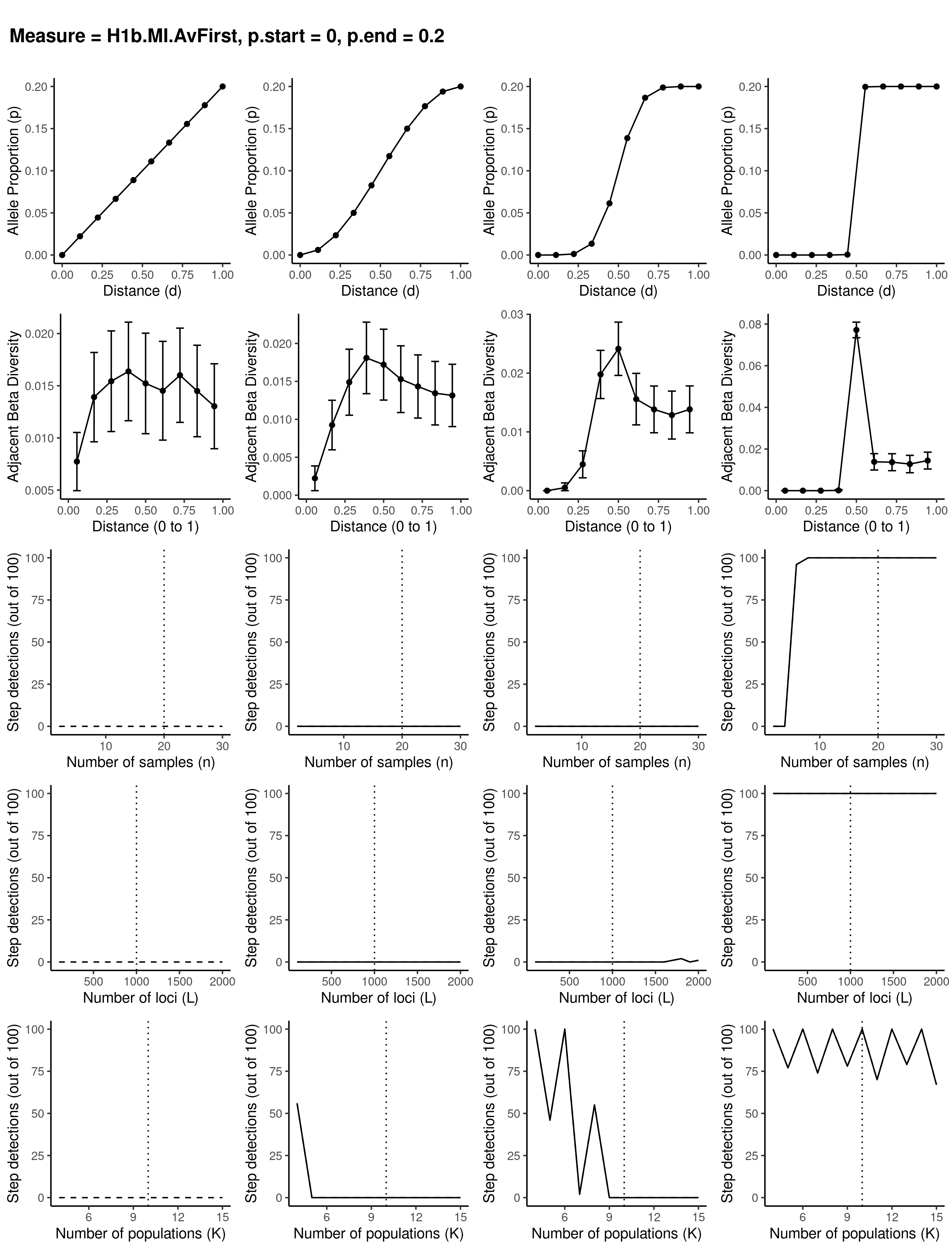
**

**S3.6.10
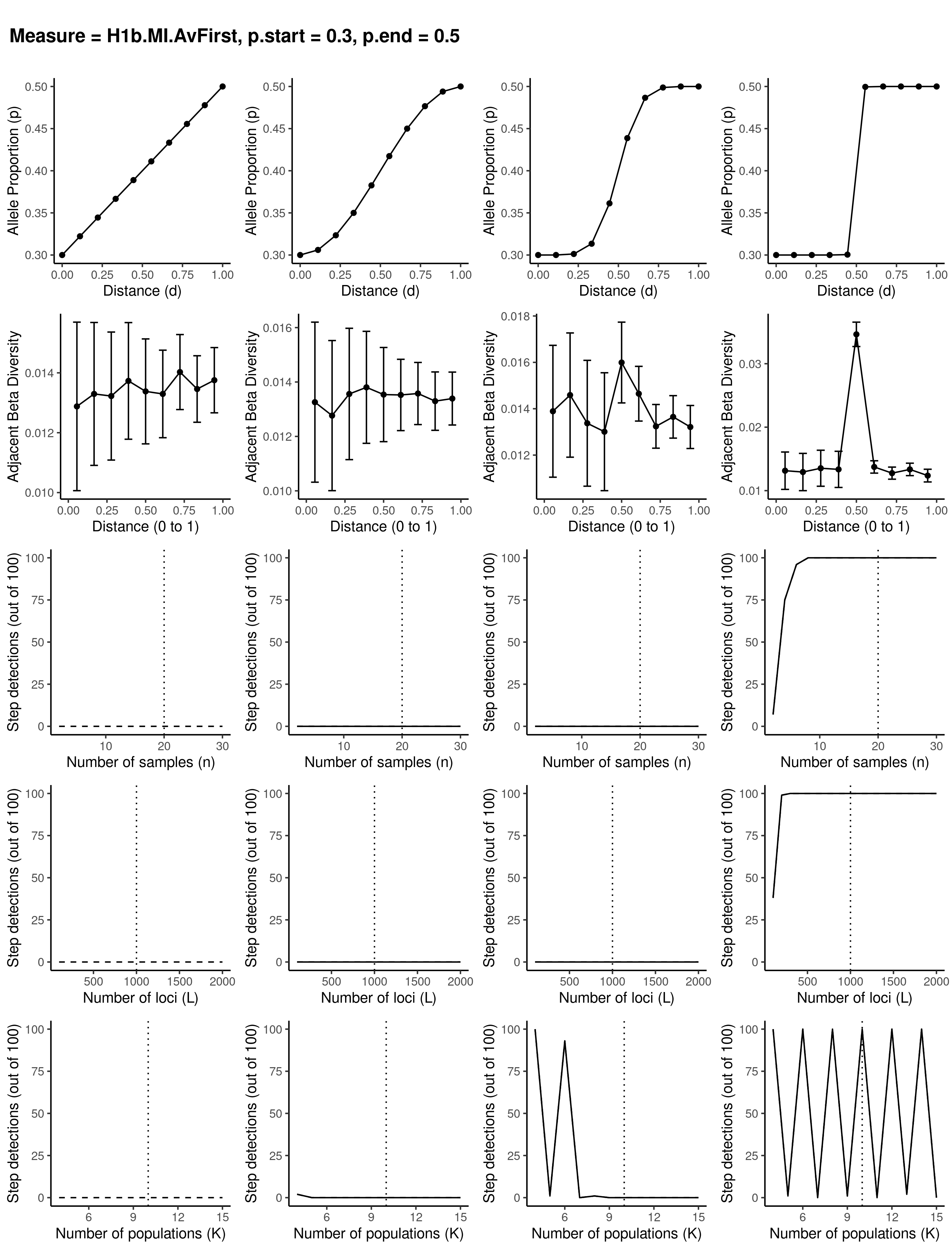
**

**S3.6.11
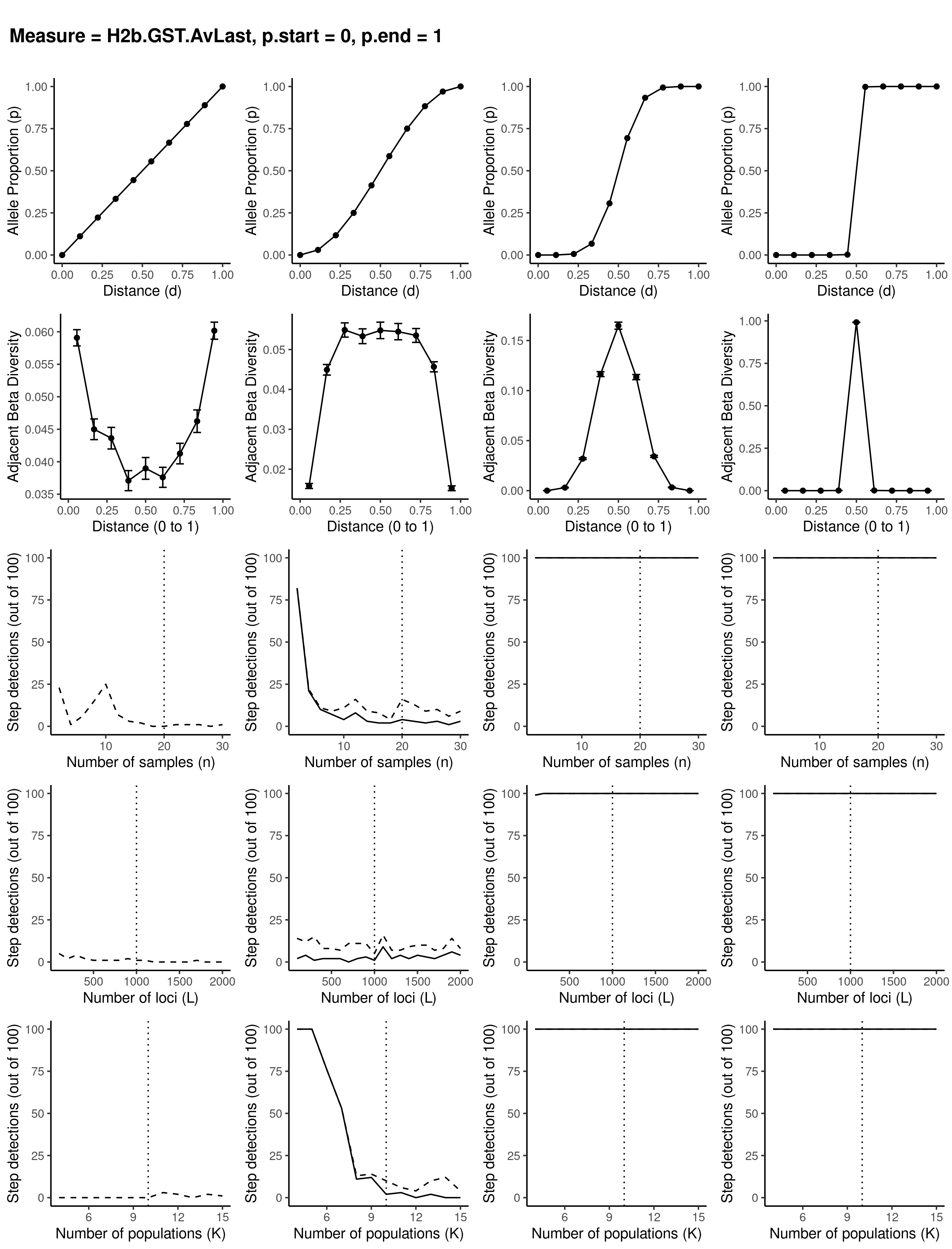
**

**S3.6.12
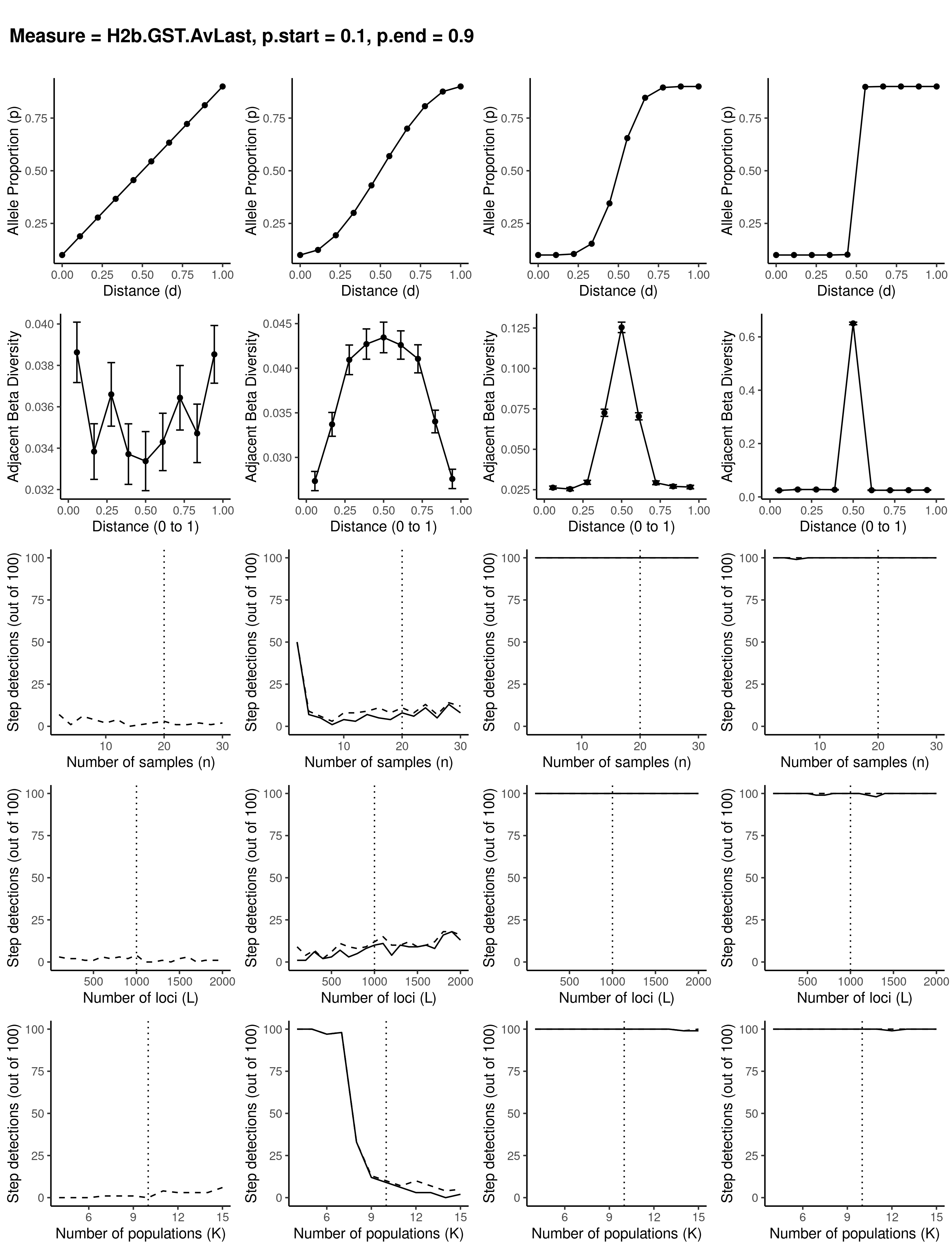
**

**S3.6.13
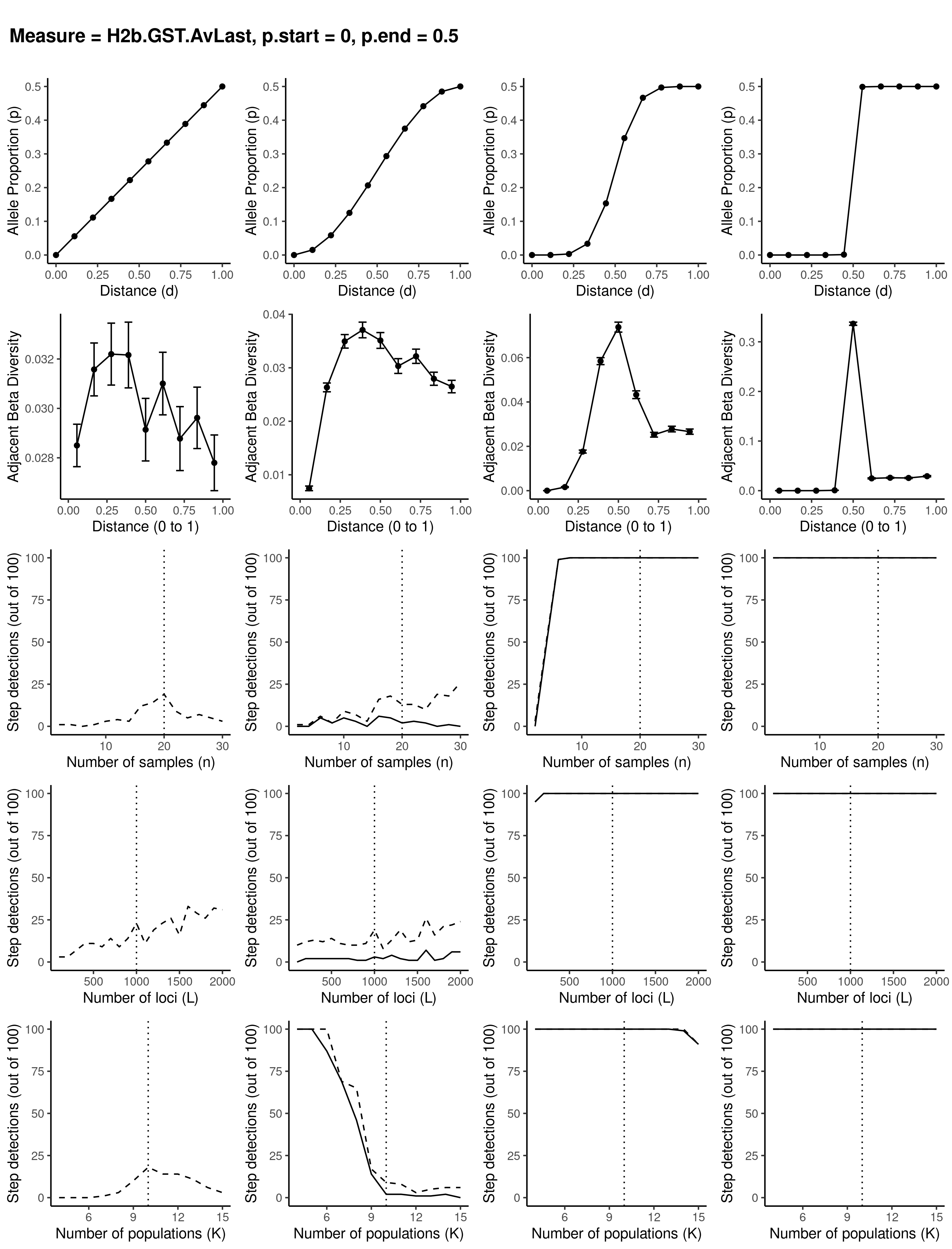
**

**S3.6.14
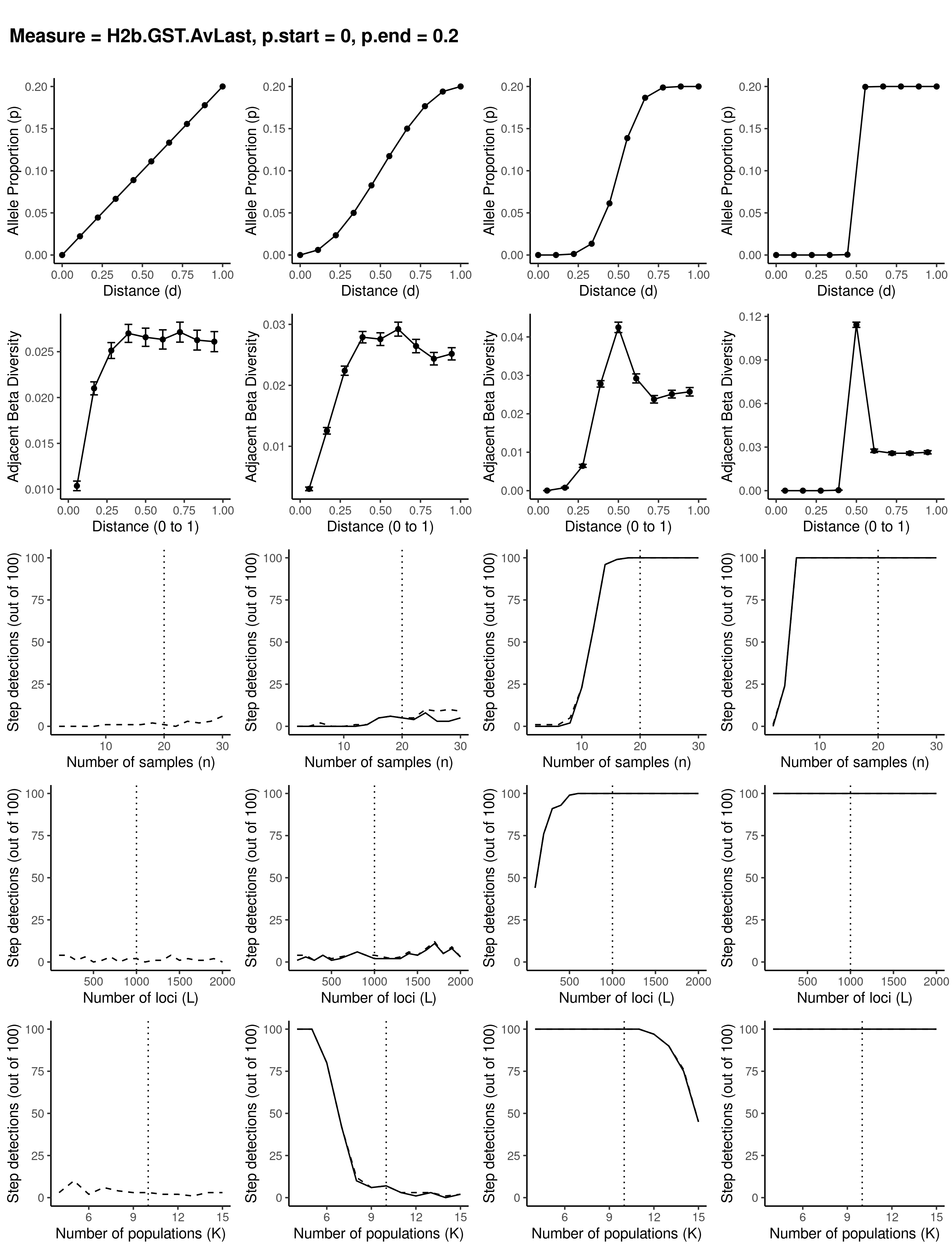
**

**S3.6.15
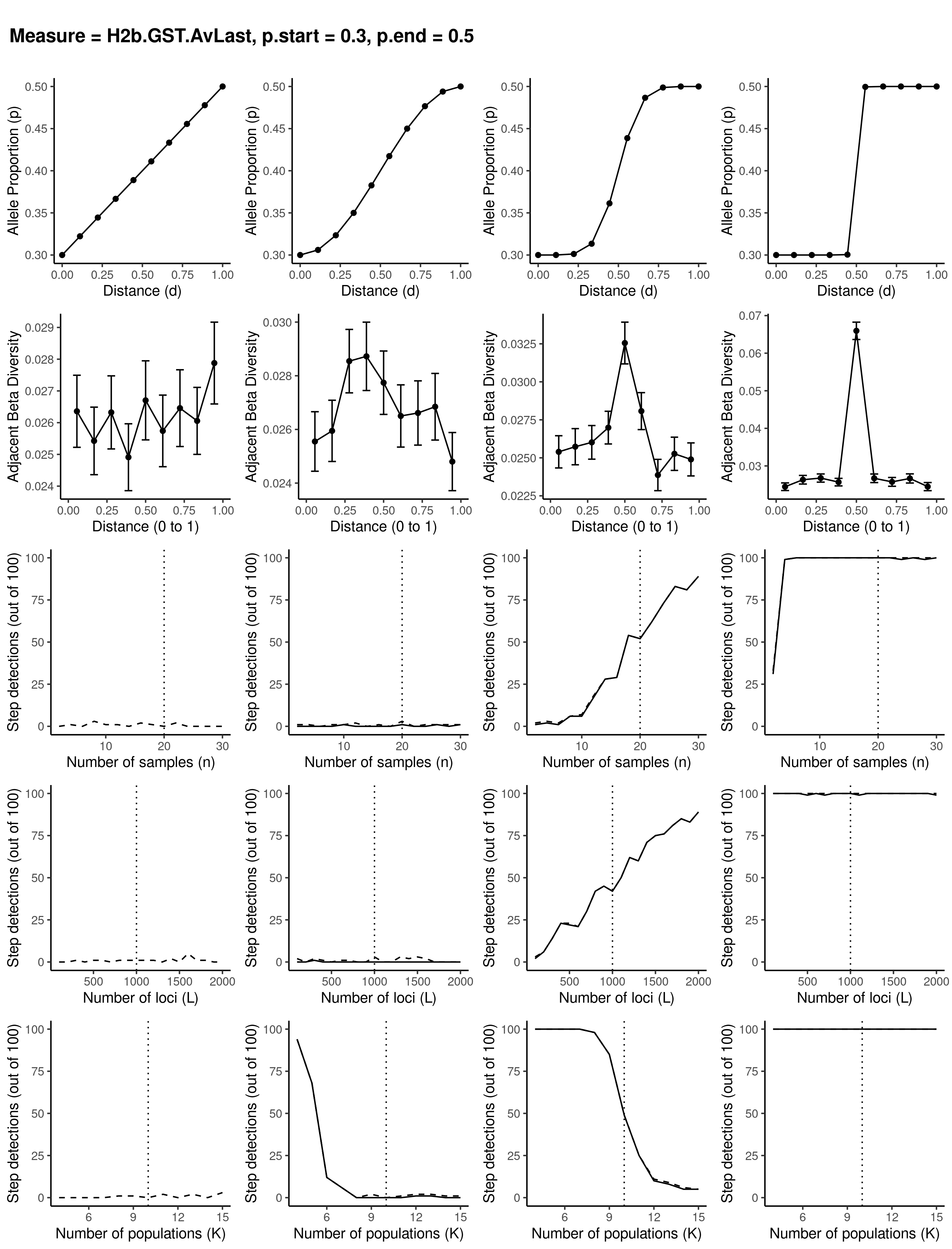
**

**S3.6.16
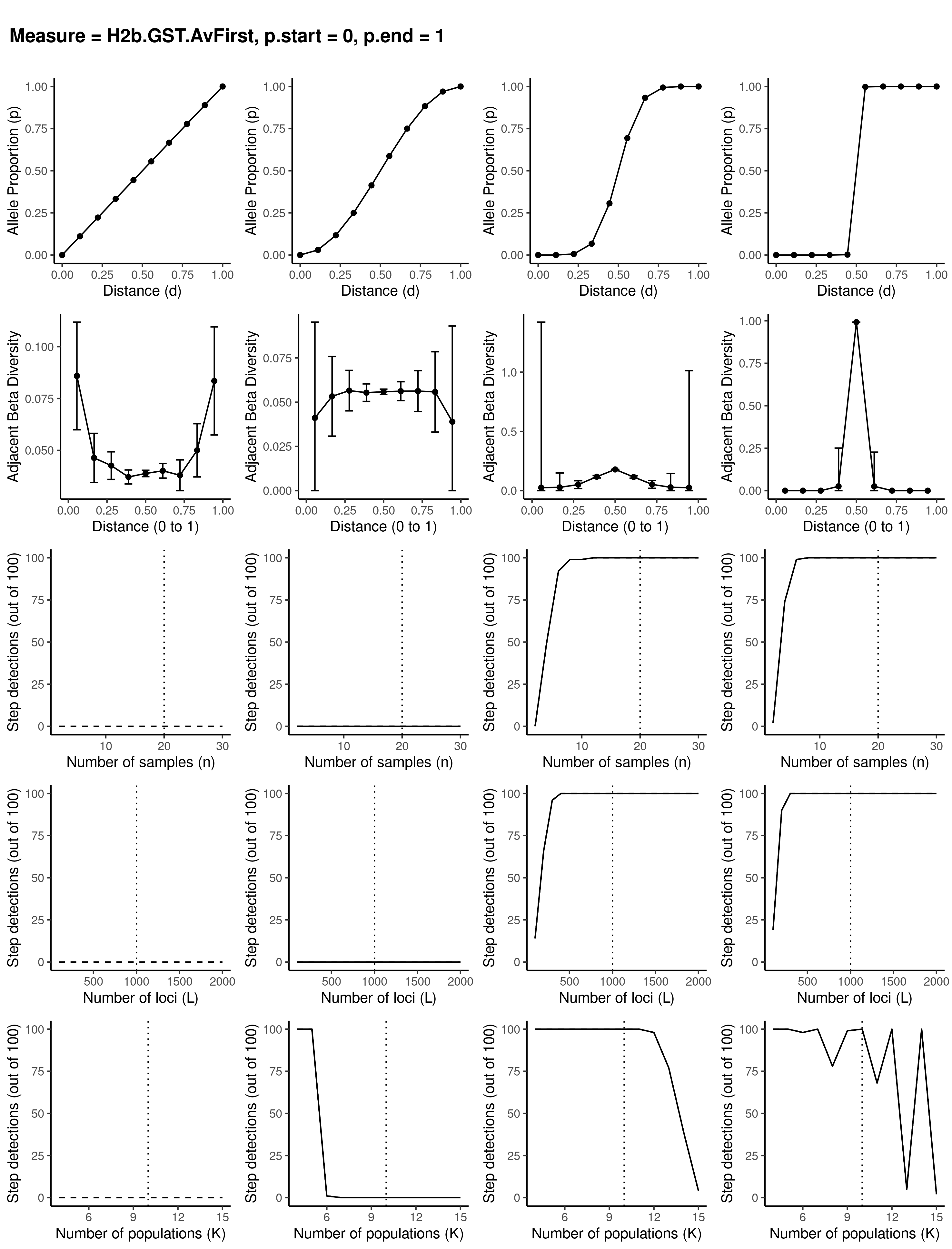
**

**S3.6.17
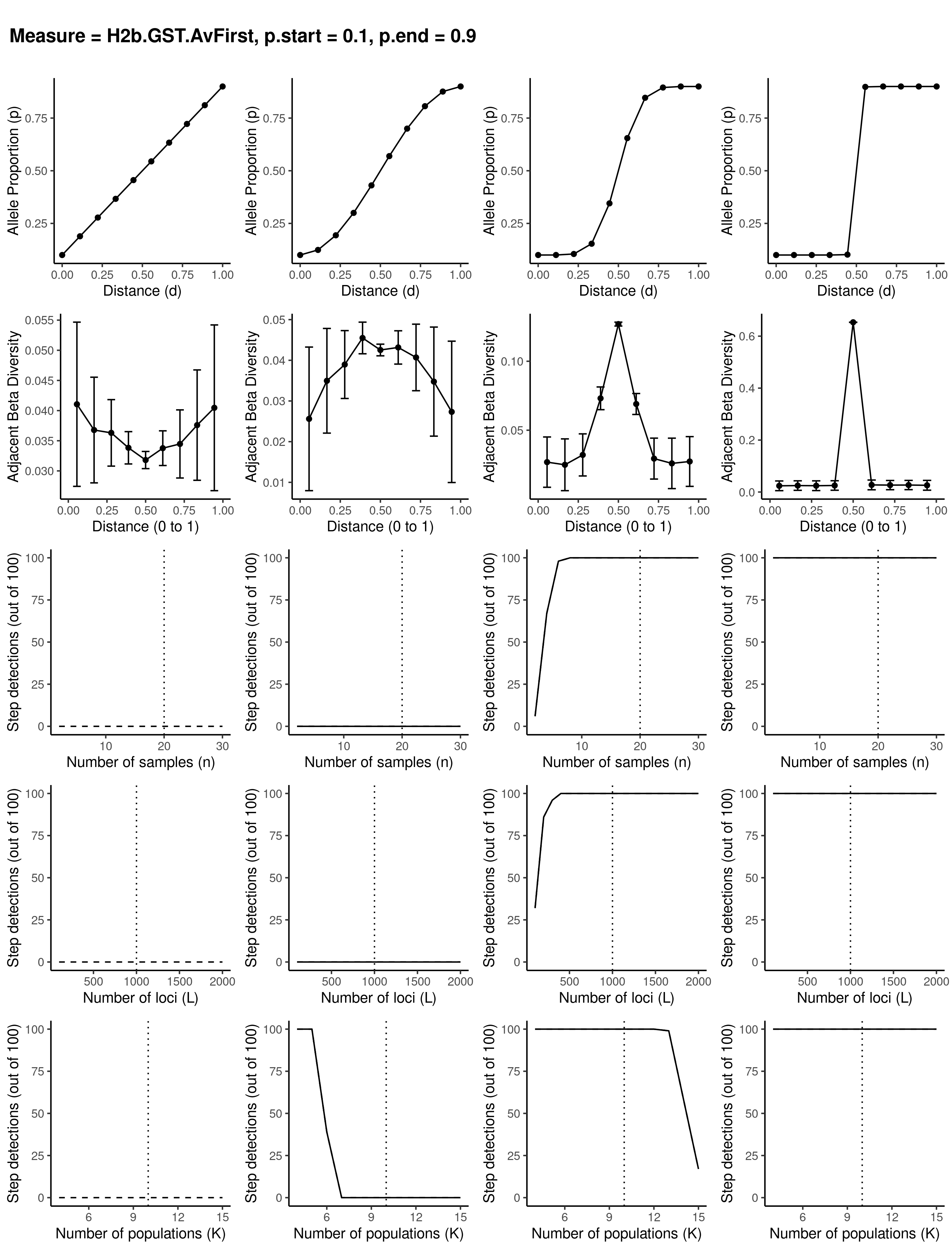
**

**S3.6.18
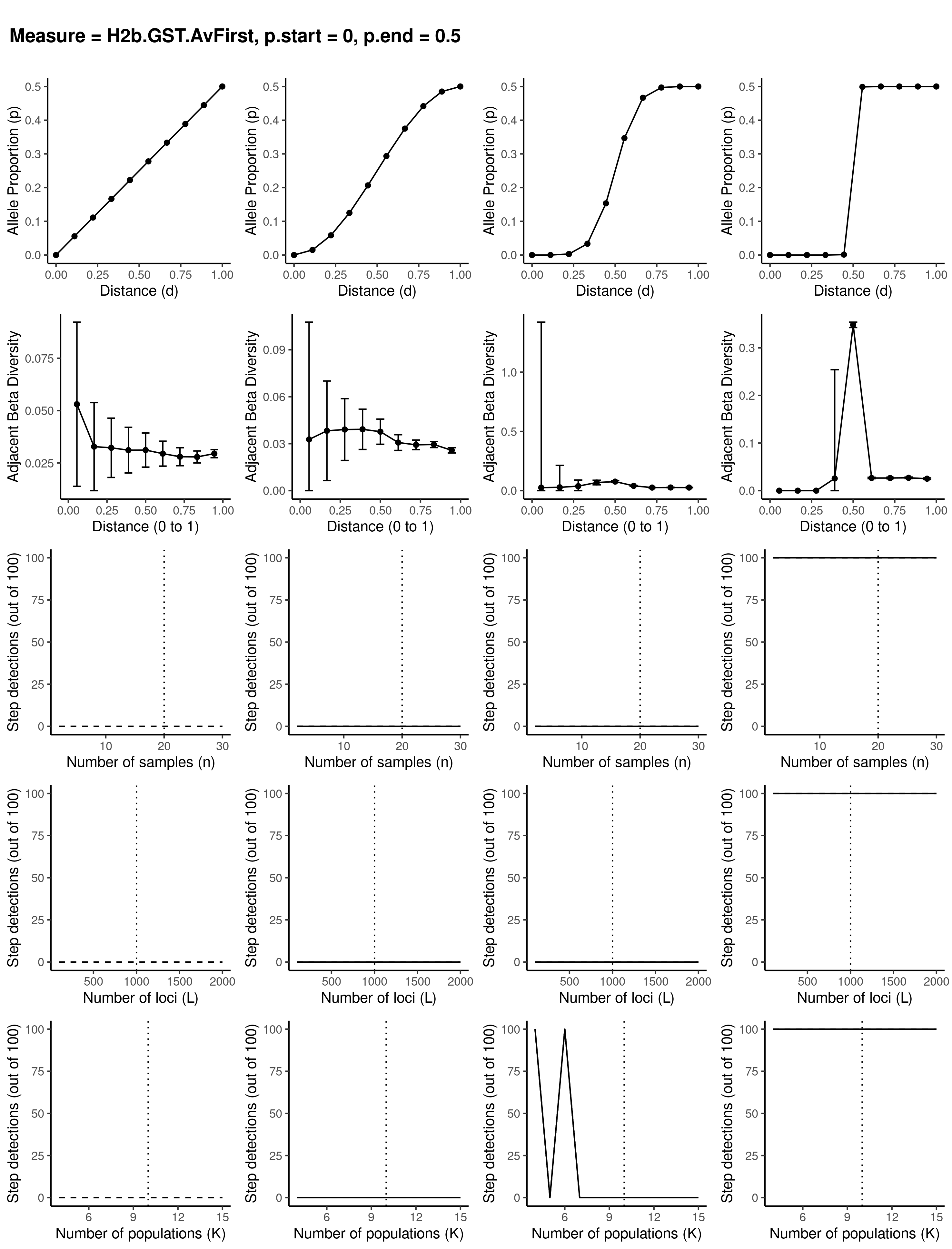
**

**S3.6.19
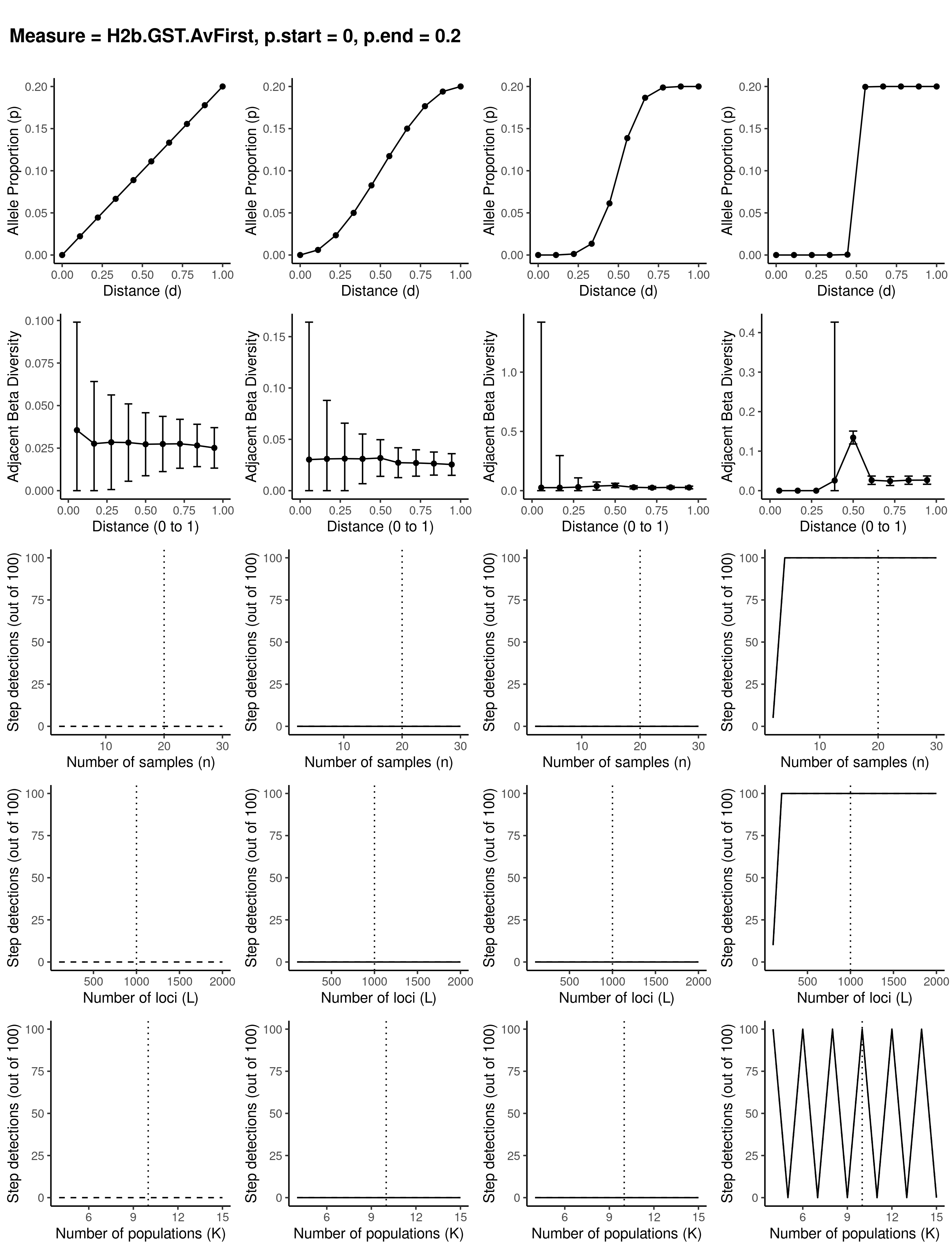
**

**S3.6.20
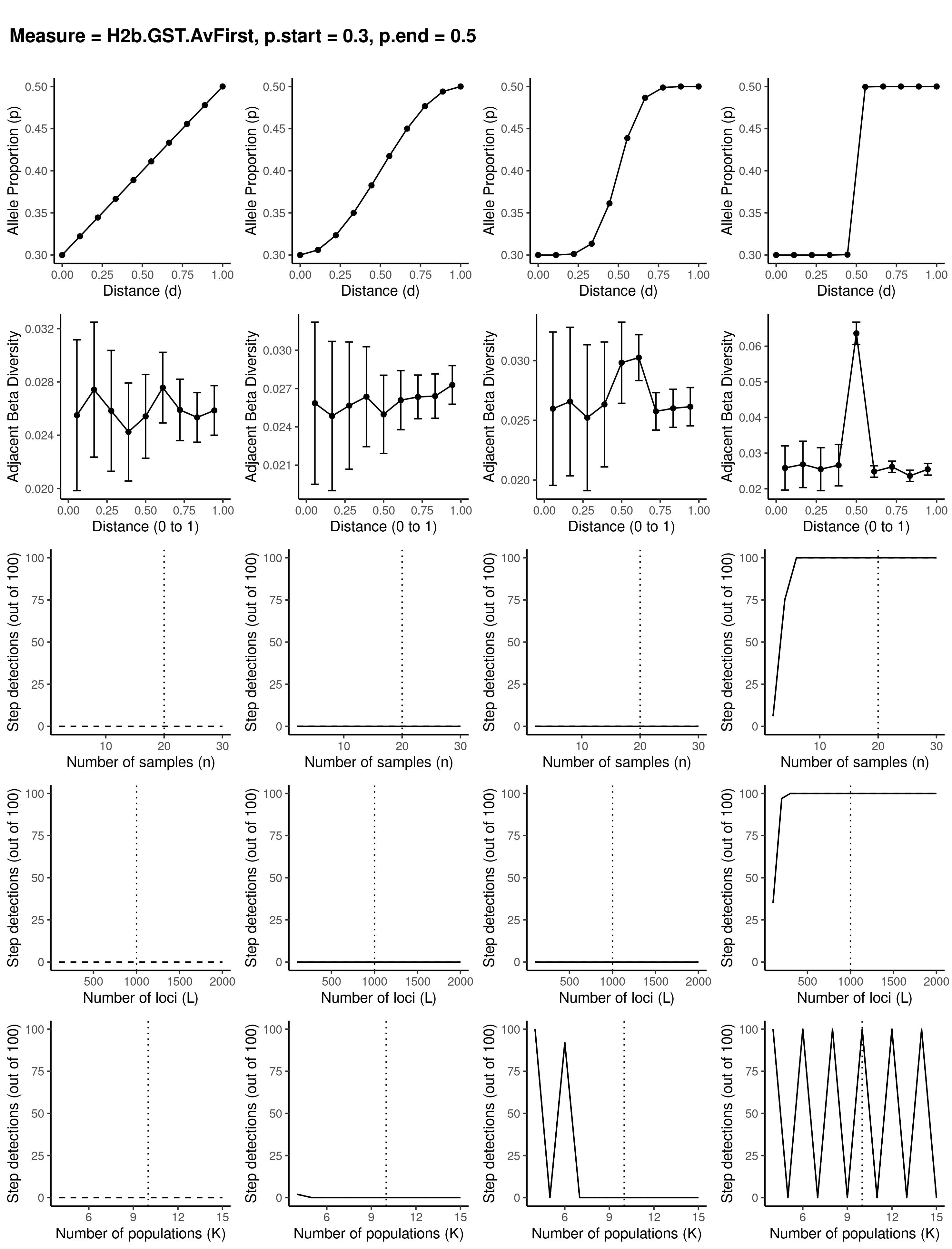
**

**S3.6.21
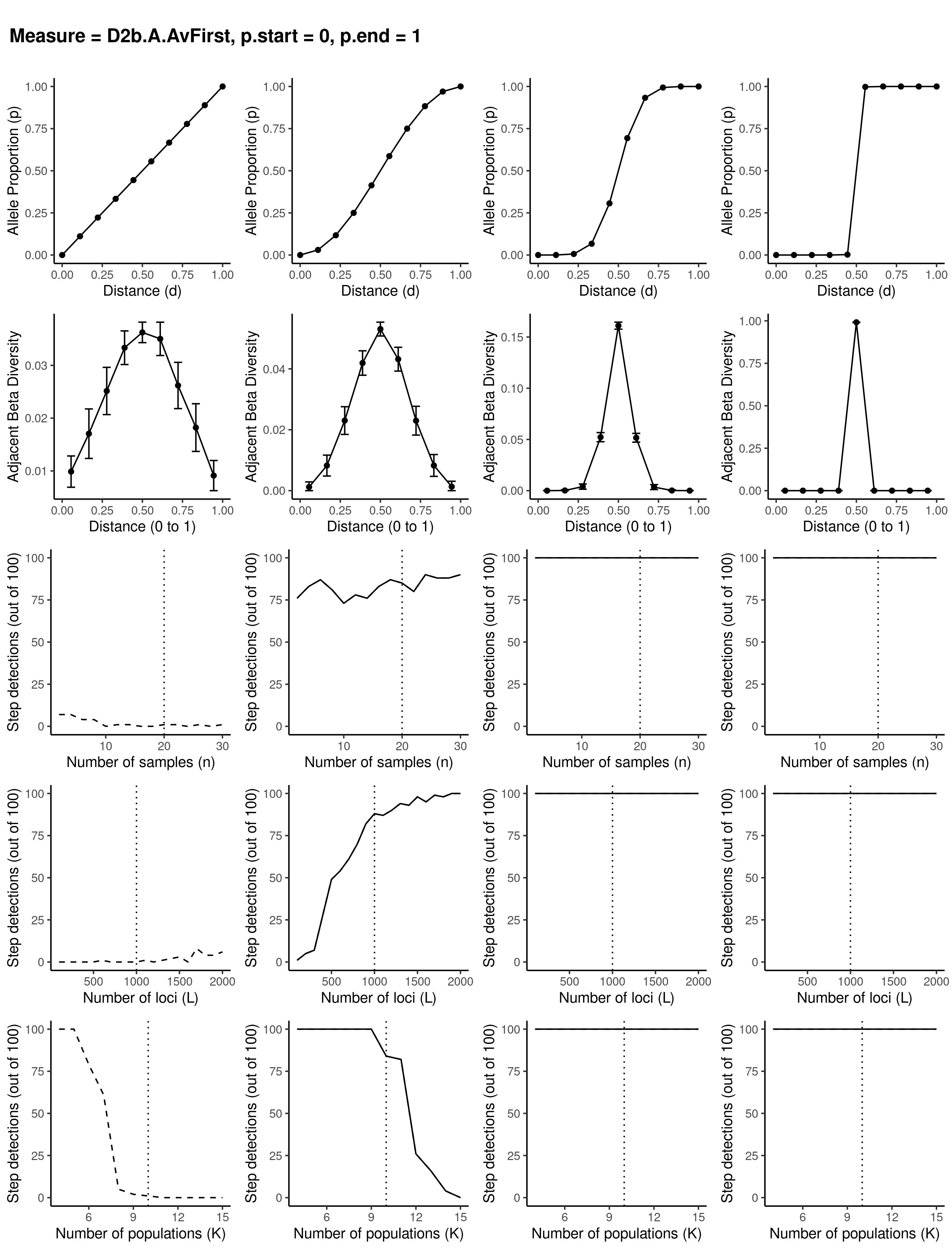
**

**S3.6.22
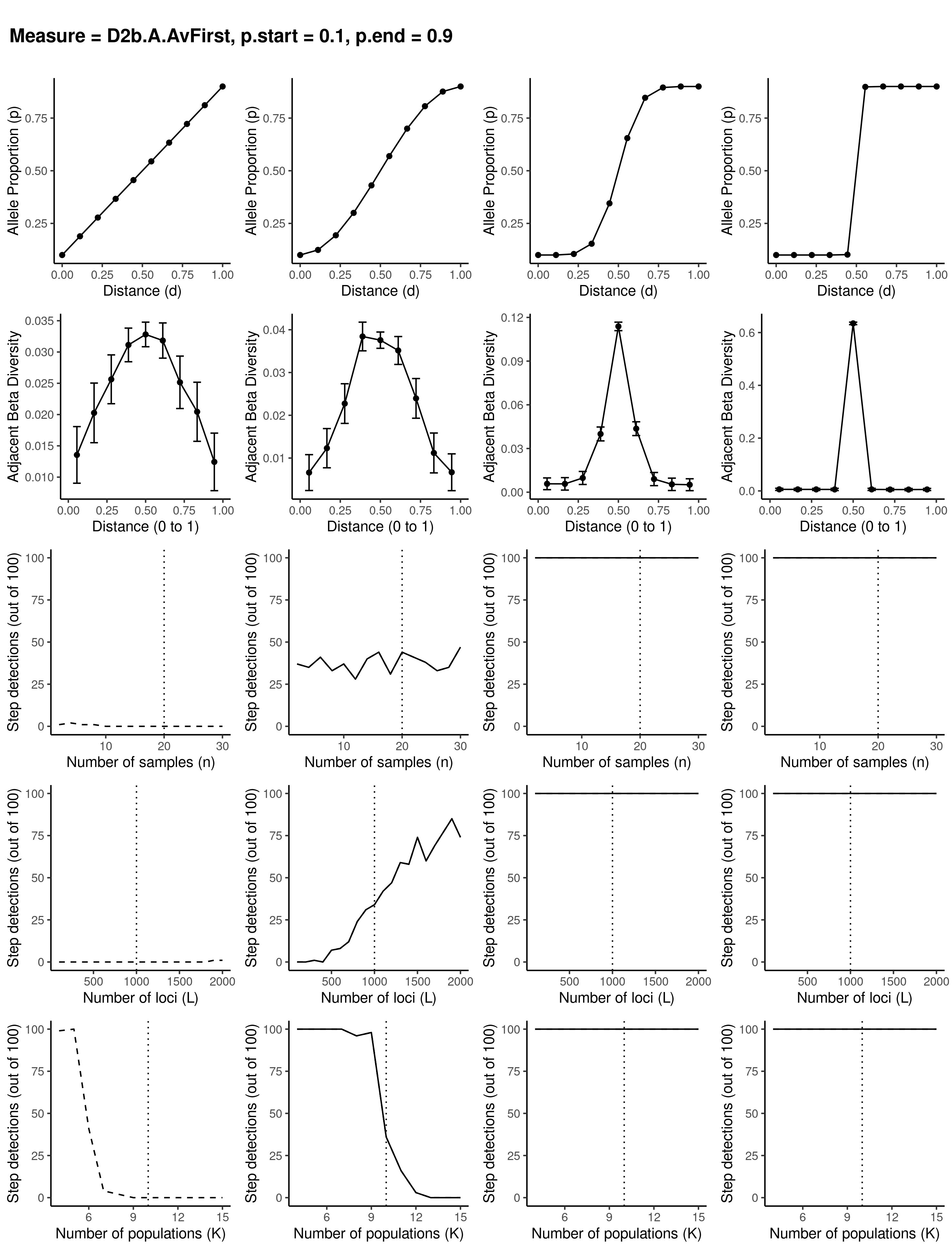
**

**S3.6.23
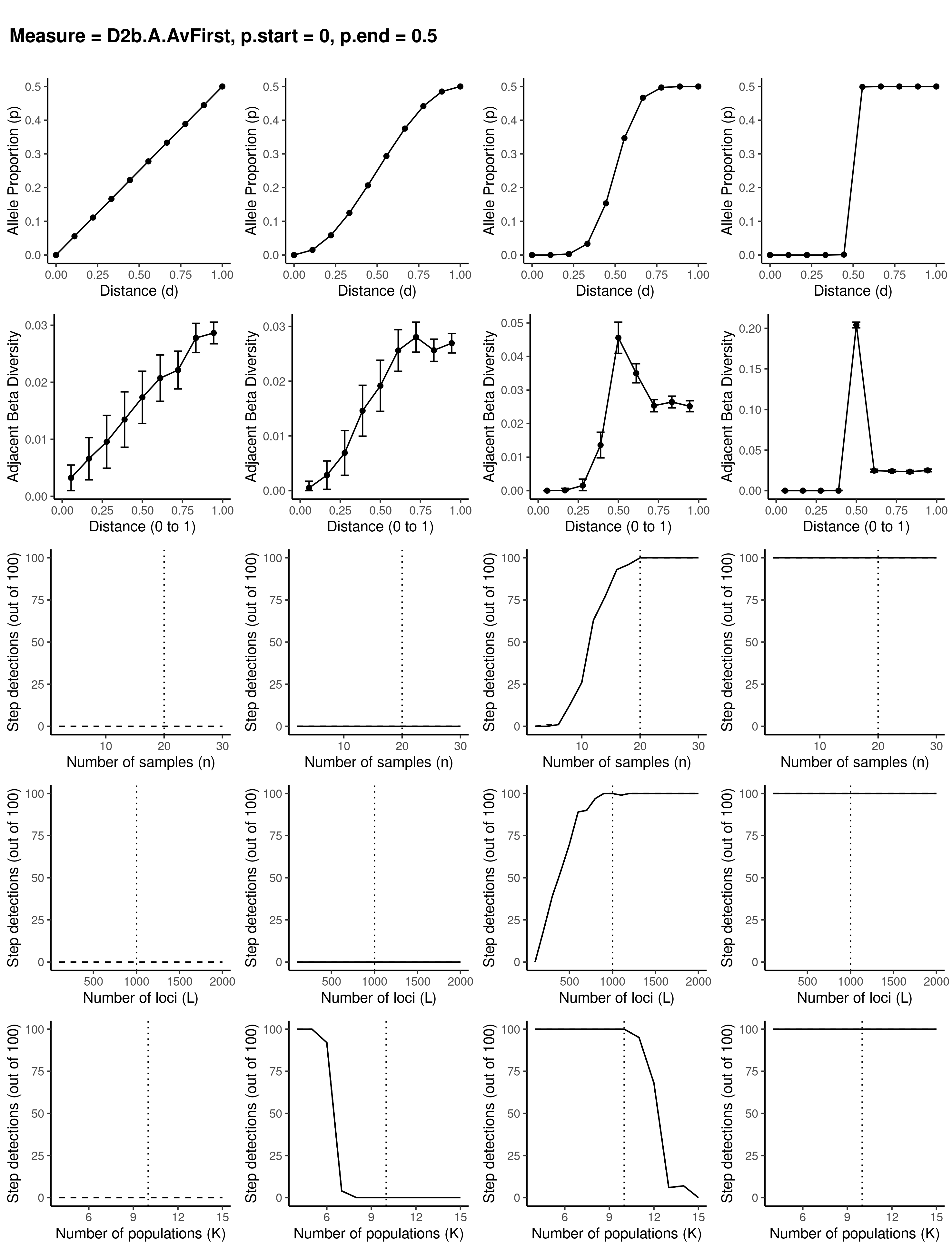
**

**S3.6.24
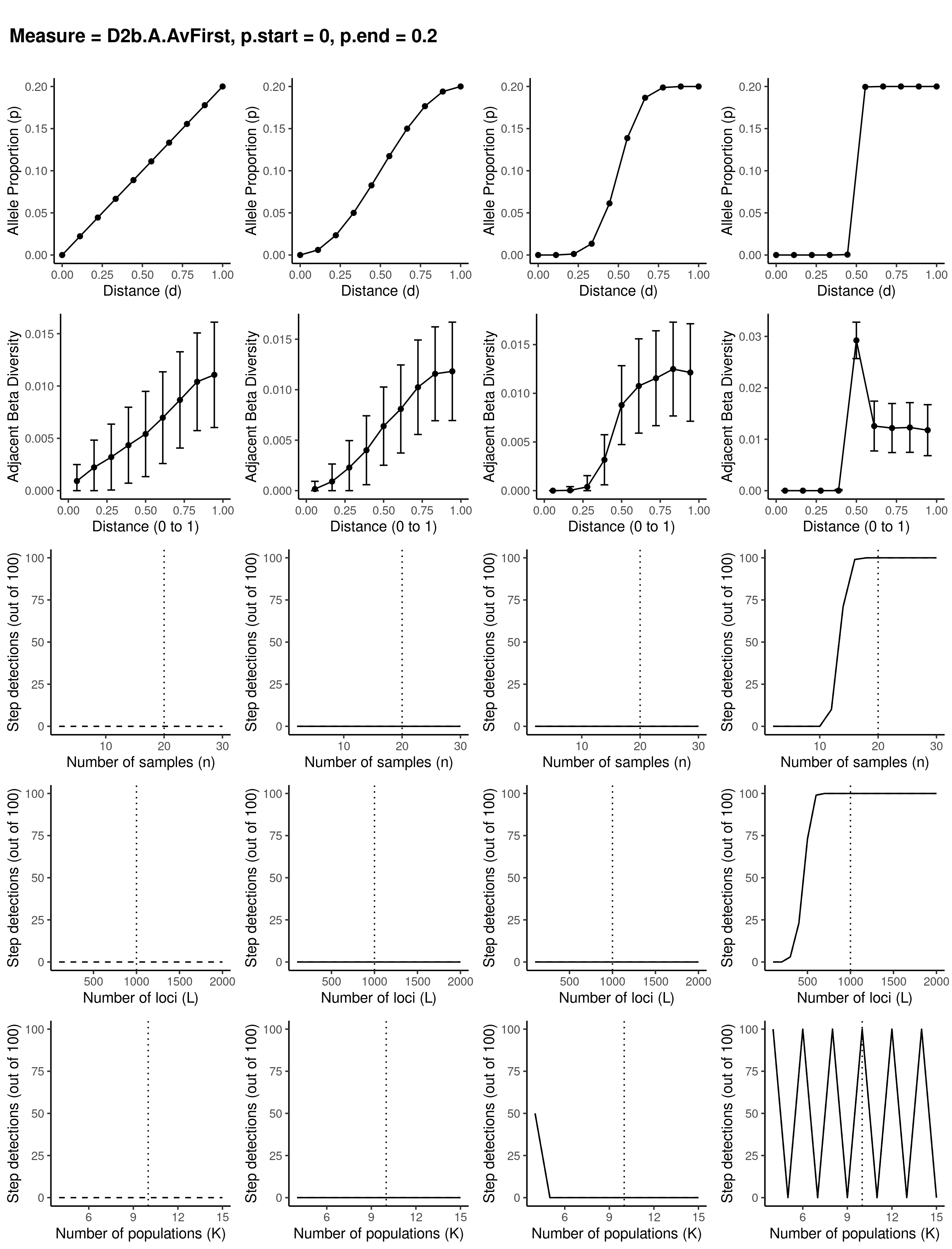
**

**S3.6.25
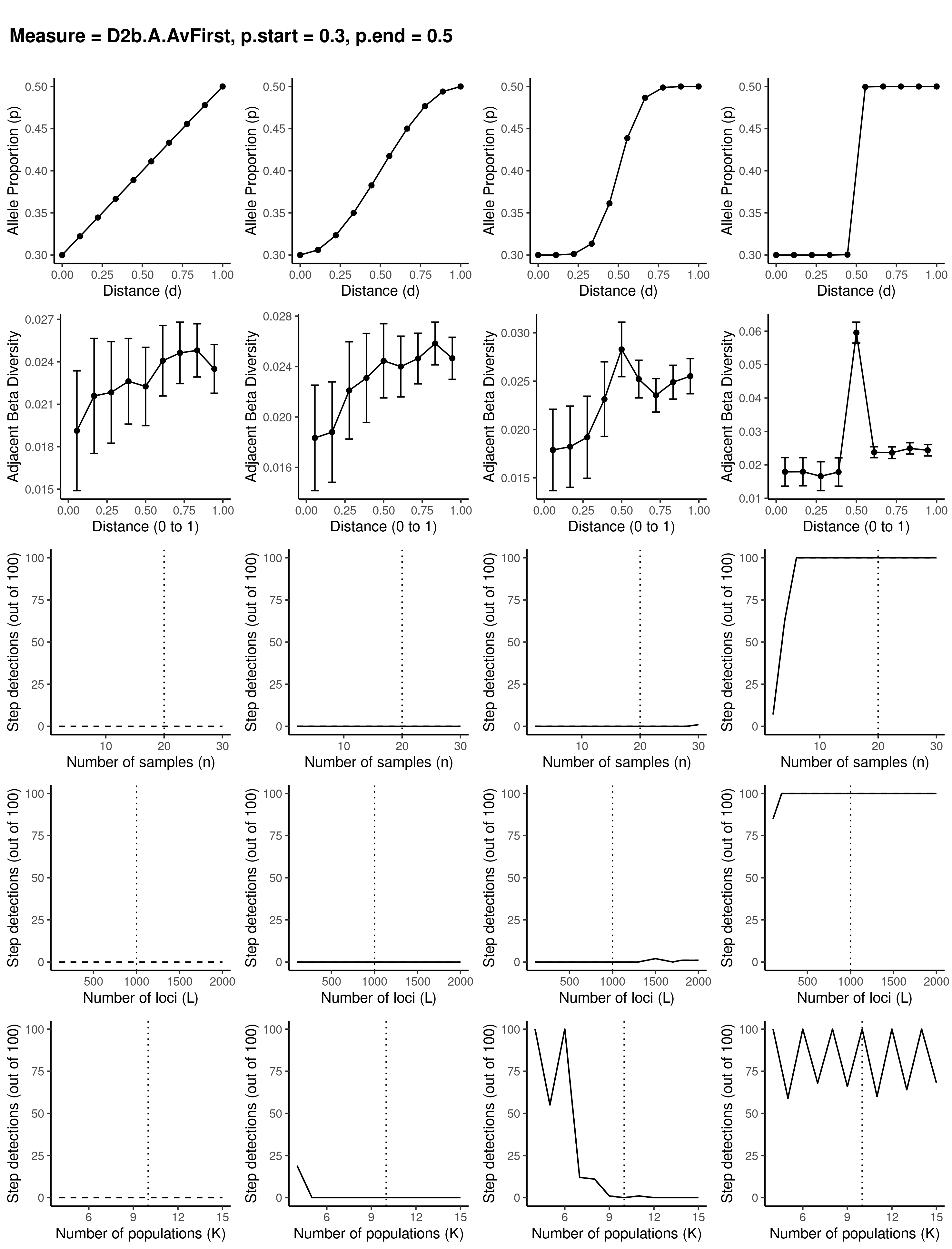
**

**S3.6.26
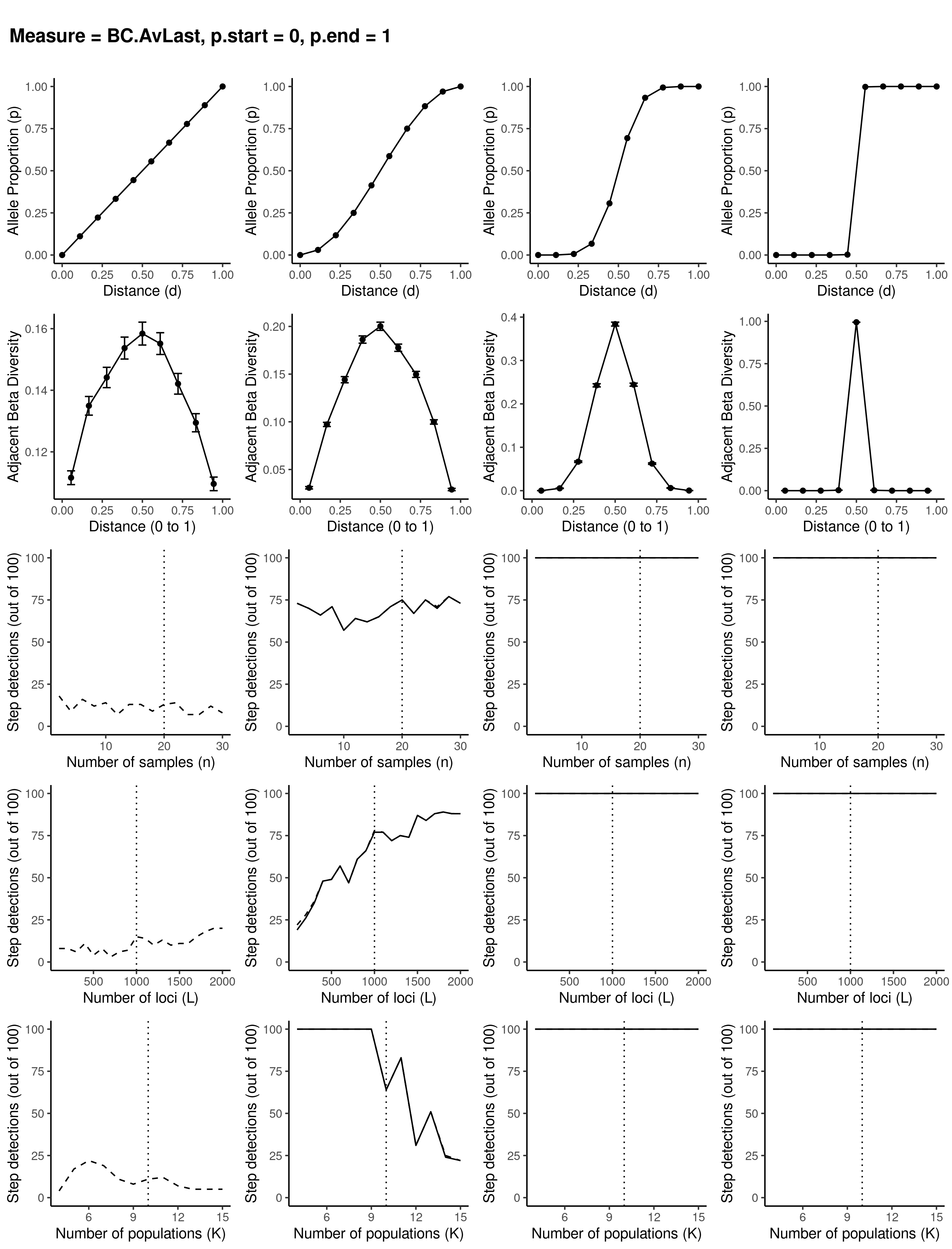
**

**S3.6.27
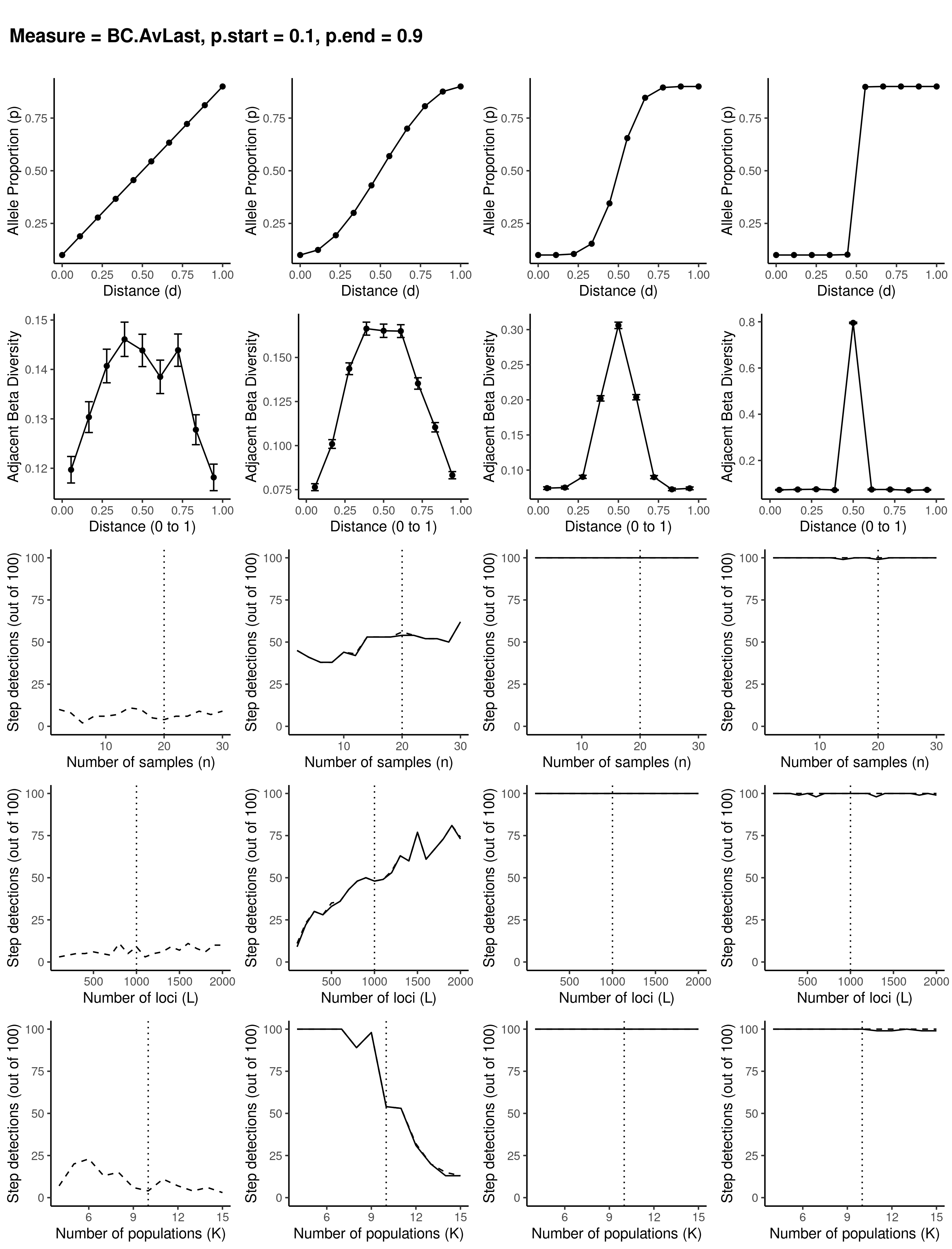
**

**S3.6.28
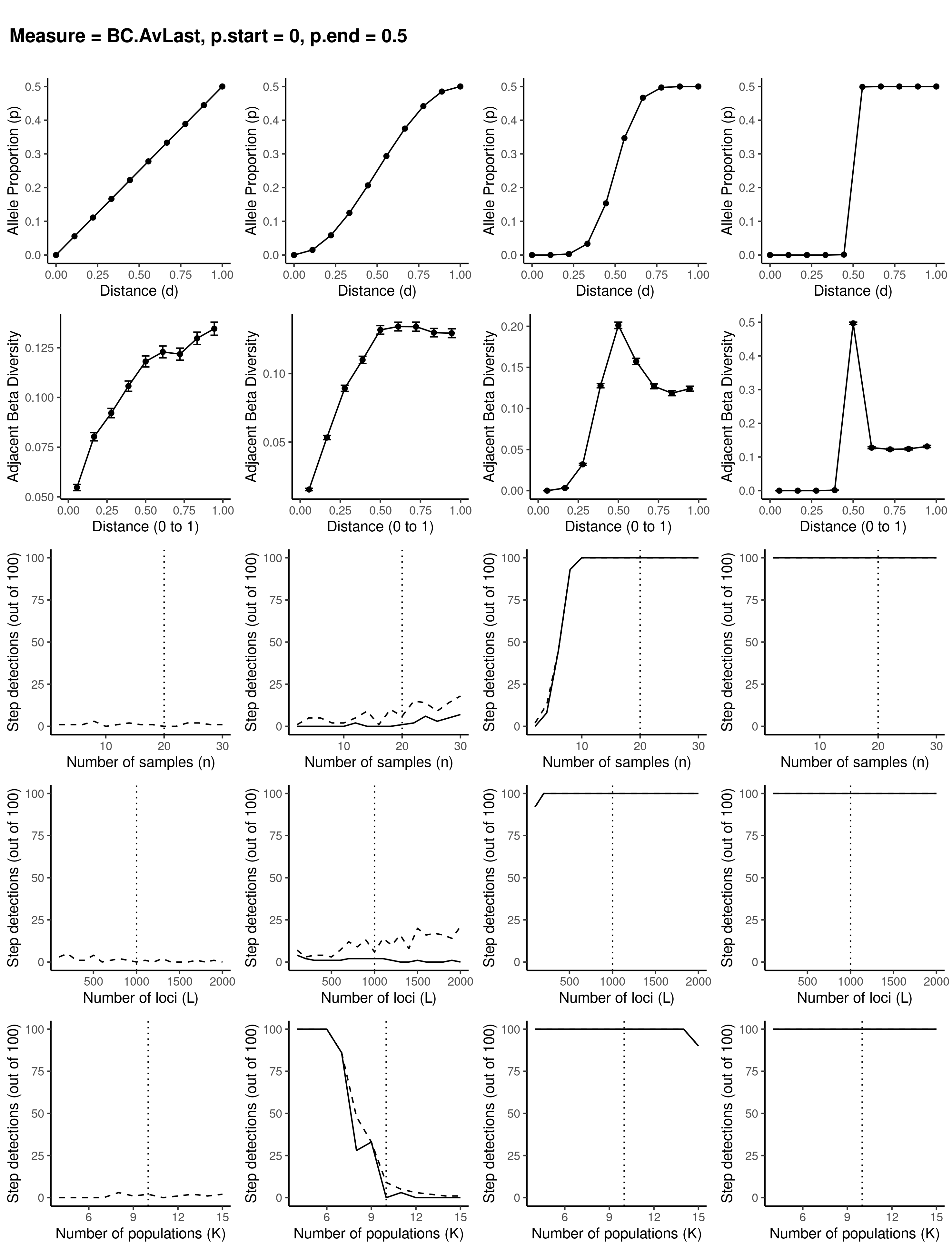
**

**S3.6.29
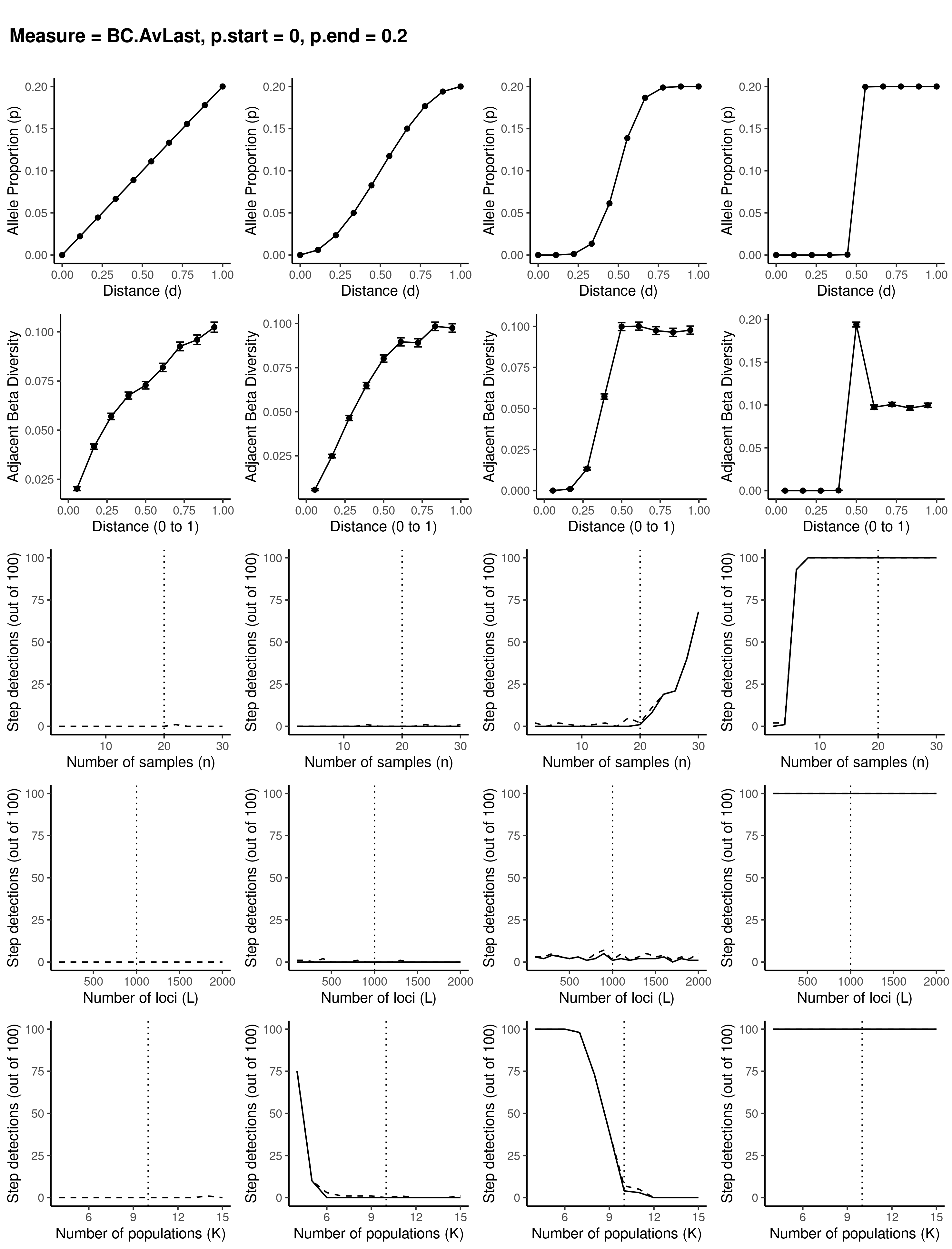
**

**S3.6.30
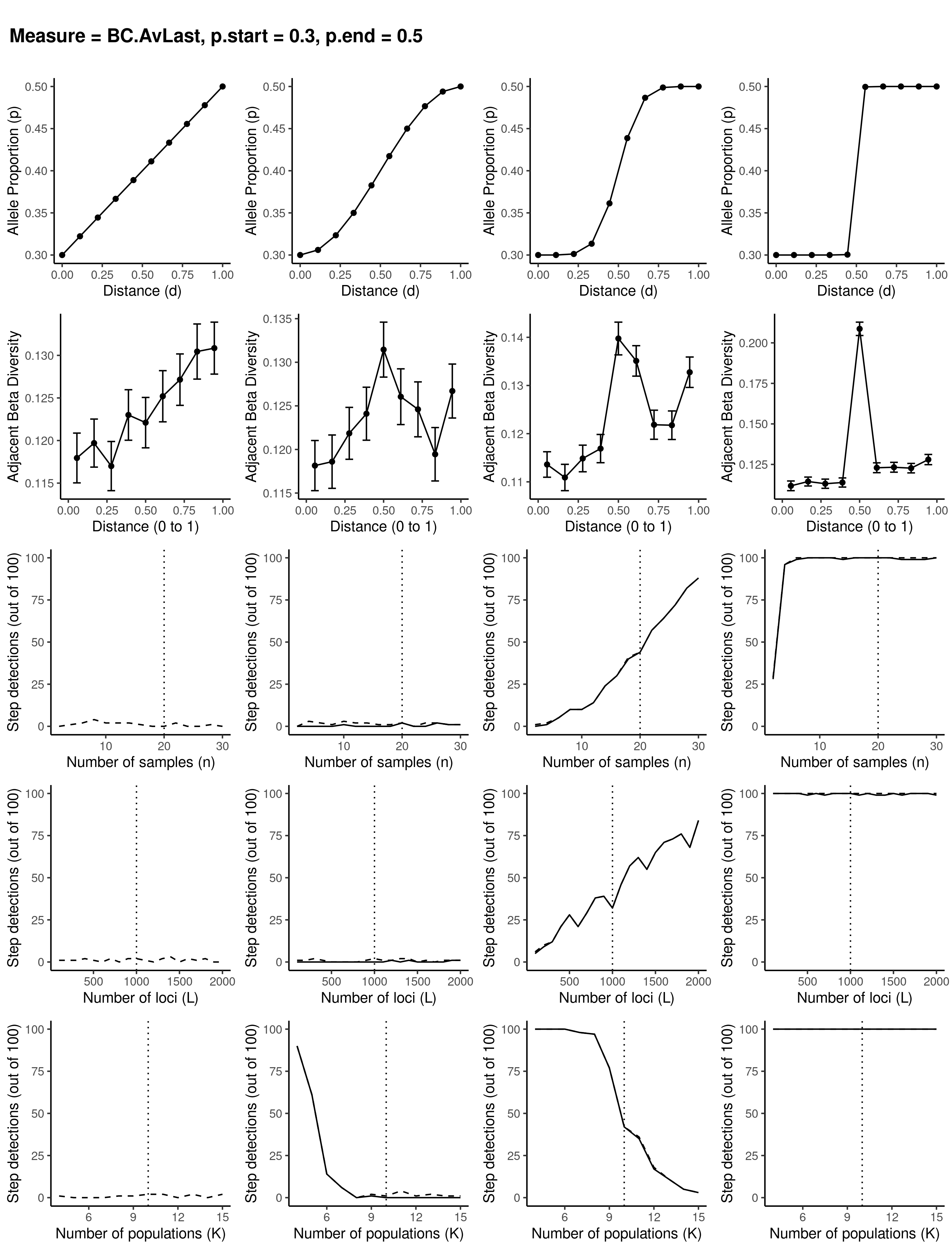
**
