# Supplementary material for: Detecting steps in spatial genetic data: Which diversity measures are best?
Source: PLoS One. 2022 Mar 14;17(3):e0265110. doi: 10.1371/journal.pone.0265110 (PMC8920294; doi:10.1371/journal.pone.0265110)
Supplement: S4 File — (DOCX) [file pone.0265110.s004.docx]

Supplemental information S4 – Code used for study

Below is the code used for this study. The packages used are listed first, followed by “the plan” script which runs all the following scripts (using the drake package).

packages.R

## These are all the packages used for this project

library(conflicted) #to avoid package conflicts
library(dotenv)
library(drake) #for project management and caching of results
library(tidyverse) #for data wrangling, plotting, iteration (purrr)
library(cowplot) #for insets in ggplot, and merging figures
library(future) #for parallel computing
library(furrr) #for using parallel computing in purrr
library(future.batchtools) #for parallel computing
library(gt)
library(kableExtra)
library(formattable)

#Which packages to prefer when there is a conflict
conflict_prefer("filter", "dplyr")

conflict_prefer("expand", "tidyr")

conflict_prefer("lag", "dplyr")

plan.R

#The plan - runs all of the code for this project

the_plan <- drake_plan(

 #Default variables for step sensitivity tests
 default_values = list(
 step = c(0, 1, 5, 50), #4 levels of step
 n.samples = 20, #default
 p.start = c(0, 0.1, 0.3), #Different allele proportion treatments
 p.end = c(0.2, 0.5, 0.9, 1), #Different allele proportion treatments
 n.loci = 1000, #default
 n.pops = 10, #default
 n_reps = 100 #replication number
 ),

 #Run simulations of all combinations of default values and varied n.samples
 step_sensitivities_samples = measure_step_detection_sensitivity(
 step = default_values$step,
 n.samples = seq(2, 30, 2), #varied input
 p.start = default_values$p.start,
 p.end = default_values$p.end,
 n.loci = default_values$n.loci,
 n.pops = default_values$n.pops,
 n_reps = default_values$n_reps
 ),

 # Run simulations of all combinations of default values and varied n.loci
 step_sensitivities_loci = measure_step_detection_sensitivity(
 step = default_values$step,
 n.samples = default_values$n.samples,
 p.start = default_values$p.start,
 p.end = default_values$p.end,
 n.loci = seq(100, 2000, 100), #varied input
 n.pops = default_values$n.pops,
 n_reps = default_values$n_reps
 ),

 # Run simulations of all combinations of default values and varied n.pops
 step_sensitivities_pops = measure_step_detection_sensitivity(
 step = default_values$step,
 n.samples = default_values$n.samples,
 p.start = default_values$p.start,
 p.end = default_values$p.end,
 n.loci = default_values$n.loci,
 n.pops = seq(4, 15, 1), #varied input
 n_reps = default_values$n_reps
 ),

 #Merge results into a single data frame
 merged_results = merge_results(step_sensitivities_samples,
 step_sensitivities_loci,
 step_sensitivities_pops),

 #Turn results into tables for publication, including all formatting
 #Standard treatment results for q = 0, q = 1,q = 2 and BC)
 results_table = get_results_table(merged_results),

 #Percentage of all step sensitivities for best 6 measures
 relative_sensitivities = extract_relative_senstivities(merged_results),

 #Summary of measures' properties, all treatments
 properties_table = make_properties_summary_table(merged_results),

 #Creates supplementary figures for all tested measures
 individual_figures = generate_sensitivity_figures(merged_results),

 alpha_peaks = vis_alpha_peaks()

 )

count_step_detections.R

Takes specific inputs of: step, n.samples, p.start, p.end, n.loci, n.pops and counts how many times each of the measures detects correct/incorrect steps repeats this “n_reps” times

Simulates new data with these variables each time and returns the sum of correct/incorrect steps across all replicates

functions it calls: simulate_data - main simulation and calculation script

count_step_detections <- function(step, n.samples, p.start, p.end,
 n.loci, n.pops, n_reps = 10) {

 # Simple function for running a simulation, then extracting the no. of steps
 extract_steps <- function(x) {

 sim_results <- simulate_data(step = step, #strength of step(s)
 n.samples = n.samples, #number of samples from each site
 p.start = p.start, #allele proportion at start
 p.end = p.end, #allele proportion at end
 n.loci = n.loci, #number of loci
 n.pops = n.pops, #number of populations
 detect_step = T)

 step_results <- sim_results$step_results

 total_steps <- as.numeric(step_results[[2]])

 correct_steps <- ifelse(step_results[[3]] <= 0.5 & step_results[[4]] >= 0.5, 1, 0)

 correct_steps <- ifelse(is.na(correct_steps), 0, correct_steps)

 names(total_steps) <- step_results[[1]]

 names(correct_steps) <- paste0(step_results[[1]], "_correct")

 output <- c(total_steps, correct_steps)

 return(output)
 }

 # Add up total number of steps
 number_of_steps <- summarise(map_dfr(1:n_reps, extract_steps),
 across(1:76, sum))

 return(number_of_steps)
}

create_sensitivities_figure.R

create_sensitivities_figure <- function(measures = c("H1b.MI.AvLast"),
 colours = c("black"),
 p.start = 0,
 p.end = 1,
 n.samples = 20,
 n.loci = 1000,
 n.pops = 10) {

 #Plot the intensity of a step as Distance (d) vs. Allele Proportion (p)
 vis.steps <- function(step) {

 step_plot <- ggplot(data = simulate_data(step = step,
 p.start = p.start,
 p.end = p.end,
 n.samples = n.samples,
 n.loci = n.loci,
 n.pops = n.pops)[[2]],
 aes(d, p_mean)) +
 geom_point() +
 geom_line() +
 xlab("Distance (d)") +
 ylab("Allele Proportion (p)") +
 theme_classic()

 return(step_plot)
 }

 #Plot the step sensitivities while varying an input (n.samples, n.loci, n.pops)
 vis.sensitivities <- function(step, variable) {

 #Which tested variable is being visualised?
 if (variable == "n.samples") {
 data <- readd(step_sensitivities_samples)

 label_for_x <- "Number of samples (n)"
 }

 if (variable == "n.loci") {
 data <- readd(step_sensitivities_loci)

 label_for_x <- "Number of loci (L)"
 }

 if (variable == "n.pops") {
 data <- readd(step_sensitivities_pops)

 label_for_x <- "Number of populations (K)"
 }

 p <- ggplot()+
 ylab("Step detections (out of 100)") +
 geom_vline(xintercept = get(variable),
 linetype = "dotted") +
 ylim(0,100) +
 theme_classic()

 for (i in seq(1, length(measures))) {
 p <- p +
 geom_line(data = filter(data, step == !!step & p.start == !!p.start & p.end == !!p.end),
 aes(x= !!sym(variable), y = !!sym(measures[[i]])), colour = colours[[i]], linetype = "dashed") +
 xlab(label = label_for_x)

 if (step != 0) { #this makes sure no "correct" steps are plotted for step = 0
 p <- p +
 geom_line(data = filter(data, step == !!step & p.start == !!p.start & p.end == !!p.end),
 aes(x = !!sym(variable), y = !!sym(paste0(measures[[i]], "_correct"))), colour = colours[[i]]) +
 xlab(label = label_for_x)

 }
 }

 return(p)
 }

 #Plot an example single simulation with the specified inputs
 vis.betas <- function(step){

 beta_plot <- plot_betas(simulate_data(step = step,
 p.start = p.start,
 p.end = p.end,
 n.samples = n.samples,
 n.loci = n.loci,
 n.pops = n.pops),
 measures = measures,
 colours = colours,
 insert = F)[[1]]

 return(beta_plot)
 }

 #Make a 4 by 3 grid of plots
 step_row <- plot_grid(vis.steps(0),
 vis.steps(1),
 vis.steps(5),
 vis.steps(50),
 nrow = 1)

 samples_row <- plot_grid(
 vis.sensitivities(0, "n.samples"),
 vis.sensitivities(1, "n.samples"),
 vis.sensitivities(5, "n.samples"),
 vis.sensitivities(50, "n.samples"),
 nrow = 1
 )

 loci_row <- plot_grid(
 vis.sensitivities(0, "n.loci"),
 vis.sensitivities(1, "n.loci"),
 vis.sensitivities(5, "n.loci"),
 vis.sensitivities(50, "n.loci"),
 nrow = 1
 )

 pops_row <- plot_grid(
 vis.sensitivities(0, "n.pops"),
 vis.sensitivities(1, "n.pops"),
 vis.sensitivities(5, "n.pops"),
 vis.sensitivities(50, "n.pops"),
 nrow = 1
 )


 beta_row <- plot_grid(vis.betas(0),
 vis.betas(1),
 vis.betas(5),
 vis.betas(50),
 nrow = 1)

 title_row <- ggdraw() +
 draw_label(paste0("Measure = ", str_flatten(measures, "_"),
 ", p.start = ", p.start,
 ", p.end = ", p.end),
 fontface = 'bold', x = 0, hjust = 0) +
 theme(
 # add margin on the left of the drawing canvas,
 # so title is aligned with left edge of first plot
 plot.margin = margin(0, 0, 0, 7)
 )


 final_figure <- plot_grid(title_row, step_row, beta_row,
 samples_row, loci_row, pops_row,
 nrow = 6, rel_heights = c(0.3, 1, 1, 1, 1, 1))

 folder_name <- ifelse(length(measures) > 1,
 "Comparison",
 str_flatten(measures, "_"))

 dir.create(paste0("./Outputs/", folder_name))

 ggsave(final_figure,
 filename = paste0("./Outputs/", folder_name,
 "/", folder_name,
 "_", p.start, "_", p.end, ".pdf"),
 height = 297, width = 210, unit = "mm")


 ggsave(final_figure,
 filename = paste0("./Outputs/", folder_name,
 "/", folder_name,
 "_", p.start, "_", p.end, ".png"),
 height = 410 *0.8, width = 250, unit = "mm")

 return(final_figure)
}

extract_relative_senstivities.R

extract_relative_senstivities <- function(merged_results) {

 #Get full dataset of all simulations
 data <- merged_results

 #Vector stating the order you want the measures to be displayed
 measure_order <- c("H1b.MI.AvLast", "H1b.MI.AvFirst",
 "H2b.GST.AvLast", "H2b.GST.AvFirst",
 "D2b.A.AvFirst","BC.AvLast")

 #Filter down to the six best measures
 data <- data %>%
 filter(Measure %in% measure_order)

 #Make a column for false positives
 #When step is zero, all steps detected are false
 #Otherwise only those not at 0.5 are false
 data <- data %>%
 mutate(false_pos = ifelse(step == 0, Total.Steps, Incorrect.Steps),
 true_pos = ifelse(step == 0, NA, Correct.Steps))

 #Split into individual allele proportion treatments
 data_0_1 <- data %>%
 filter(p.start == 0) %>%
 filter(p.end == 1) %>%
 group_by(Measure, step) %>%
 summarise(true_pos = round(sum(true_pos)/(n()), digits = 1),
 false_pos = round(sum(false_pos)/(n()), digits = 1)) %>%
 pivot_longer(names_to = "step_type", cols = c(true_pos, false_pos)) %>%
 pivot_wider(names_from = step, values_from = value) %>%
 rename_with(.cols = -c(Measure, step_type), ~ paste(.x, "0_1", sep = "_"))

 data_0.1_0.9 <- data %>%
 filter(p.start == 0.1) %>%
 filter(p.end == 0.9) %>%
 group_by(Measure, step) %>%
 summarise(true_pos = round(sum(true_pos)/(n()), digits = 1),
 false_pos = round(sum(false_pos)/(n()), digits = 1)) %>%
 pivot_longer(names_to = "step_type", cols = c(true_pos, false_pos)) %>%
 pivot_wider(names_from = step, values_from = value) %>%
 rename_with(.cols = -c(Measure, step_type), ~ paste(.x, "0.1_0.9", sep = "_"))


 data_0_0.5 <- data %>%
 filter(p.start == 0) %>%
 filter(p.end == 0.5)%>%
 group_by(Measure, step) %>%
 summarise(true_pos = round(sum(true_pos)/(n()), digits = 1),
 false_pos = round(sum(false_pos)/(n()), digits = 1)) %>%
 pivot_longer(names_to = "step_type", cols = c(true_pos, false_pos)) %>%
 pivot_wider(names_from = step, values_from = value) %>%
 rename_with(.cols = -c(Measure, step_type), ~ paste(.x, "0_0.5", sep = "_"))


 data_0_0.2 <- data %>%
 filter(p.start == 0) %>%
 filter(p.end == 0.2) %>%
 group_by(Measure, step) %>%
 summarise(true_pos = round(sum(true_pos)/(n()), digits = 1),
 false_pos = round(sum(false_pos)/(n()), digits = 1)) %>%
 pivot_longer(names_to = "step_type", cols = c(true_pos, false_pos)) %>%
 pivot_wider(names_from = step, values_from = value) %>%
 rename_with(.cols = -c(Measure, step_type), ~ paste(.x, "0_0.2", sep = "_"))


 data_0.3_0.5 <- data %>%
 filter(p.start == 0.3) %>%
 filter(p.end == 0.5) %>%
 group_by(Measure, step) %>%
 summarise(true_pos = round(sum(true_pos)/(n()), digits = 1),
 false_pos = round(sum(false_pos)/(n()), digits = 1)) %>%
 pivot_longer(names_to = "step_type", cols = c(true_pos, false_pos)) %>%
 pivot_wider(names_from = step, values_from = value) %>%
 rename_with(.cols = -c(Measure, step_type), ~ paste(.x, "0.3_0.5", sep = "_"))


 #Put all the data back together
 data_merge <- data_0_1 %>%
 left_join(data_0.1_0.9, by = c("Measure", "step_type"))%>%
 left_join(data_0_0.5, by = c("Measure", "step_type"))%>%
 left_join(data_0_0.2, by = c('Measure', "step_type"))%>%
 left_join(data_0.3_0.5, by = c("Measure", "step_type")) %>%
 rename_with(.cols = -c(Measure, step_type), ~ paste("step", .x, sep = "_"))


 data_merge_format <- data_merge


 data_merge_format <- data_merge_format %>%
 mutate(Measure = factor(Measure, levels = measure_order)) %>%
 # mutate(Measure = fct_relevel(Measure, measure_order)) %>%
 arrange(Measure)


 data_merge_format$Measure <- c("MI \n (AvLast)", " ", "MI \n (AvFirst)", " ",
 "GST \n (AvLast)", " ", "GST \n (AvFirst)", " ",
 "D2b.A (AvFirst)", " ", "BC (AvLast)", " ")

 data_merge_format <- data_merge_format %>%
 mutate_all(~replace(., is.na(.), "-"))


 data_merge_format$step_type[data_merge_format$step_type == "true_pos"] <- "True Positives"


 data_merge_format$step_type[data_merge_format$step_type == "false_pos"] <- "False Positives"


 #correct steps formatting
 data_merge_format[3:22] <- lapply(data_merge_format[3:22], function(x) {
 v <- 1:14
 v[c(1,3,5,7,9,11,13,14)] <- color_tile("transparent", "green")(c(x[c(1,3,5,7,9,11)], 0, 100))
 v[c(2,4,6,8,10,12,13,14)] <- color_tile("transparent", "red")(c(x[c(2,4,6,8,10,12)], 0, 100))

 return(v[1:12])
 })


 options(knitr.kable.NA = '-')


 #create basic table
 results_table <- data_merge_format %>%
 kbl(col.names = c("Measure", "Type of Step", "0", "1", "5", "50", "0", "1", "5", "50",
 "0", "1", "5", "50", "0", "1", "5", "50", "0", "1", "5", "50"),
 escape = FALSE) %>%
 kable_classic(full_width = F, html_font = "Cambria") %>%
 #Add heading

 #Remove every second Measure name

 #Replace Measure name with better formatting
 #Add stub head for each allele treatment
 add_header_above(c(" " = 2,
 "Maximal \n range: \n p = 0 - 1" = 4,
 "Maximal range \n without fixation: \n p = 0.1 - 0.9" = 4,
 "Halfmaximal \n range: \n p = 0 - 0.5" = 4,
 "Narrow range \n near fixation: \n p = 0 - 0.2" = 4,
 "Narrow range \n far from fixation: \n p = 0.3 - 0.5" = 4)
 )
 #Make Step names shorter
 #Add conditional formatting

 results_table

 save_kable(results_table, file = "./Outputs/relative_sensitivities_table.html")


 return(results_table)
}

generate_sensitivity_figures.R

generate_sensitivity_figures <- function(step_sensitivities_samples,
 step_sensitivities_loci,
 step_sensitivities_pops) {

 measures <- c("H0b.Jac.AvLast", "H0b.Sor.AvLast",
 "D0b.A.AvLast", "D0b.B.AvLast",
 "H0b.Jac.AvFirst", "H0b.Sor.AvFirst",
 "D0b.A.AvFirst", "D0b.B.AvFirst",
 "H1b.MI.AvLast", "H1b.ShD.AvLast",
 "D1b.A.AvLast", "D1b.B.AvLast",
 "H1b.MI.AvFirst", "H1b.ShD.AvFirst",
 "D1b.A.AvFirst", "D1b.B.AvFirst",
 "H2b.GST.AvLast", "H2b.JOST.AvLast",
 'D2b.A.AvLast', 'D2b.B.AvLast',
 "H2b.GST.AvFirst", "H2b.JOST.AvFirst",
 'D2b.A.AvFirst', 'D2b.B.AvFirst',
 "BC.AvLast") #skip relative measures

 #Create pdf of results for each measure, for each allele treatment
 figs1 <- map(measures, create_sensitivities_figure, p.start = 0, p.end = 1)

 figs2 <- map(measures, create_sensitivities_figure, p.start = 0.1, p.end = 0.9)

 figs3 <- map(measures, create_sensitivities_figure, p.start = 0, p.end = 0.5)

 figs4 <- map(measures, create_sensitivities_figure, p.start = 0, p.end = 0.2)

 figs5 <- map(measures, create_sensitivities_figure, p.start = 0.3, p.end = 0.5)

 #After saving the initial files, put them all together in one pdf (130 pages)

 #add them all to a list
 l <- c(figs1, figs2, figs3, figs4, figs5)

 pdf("Outputs/all_supp_figs.pdf")
 invisible(lapply(l, print))
 dev.off()

}

get_results_table.R

Takes merged_results and creates Table 2 results under standard conditions

get_results_table <- function(merged_results){

 #Get full dataset of all simulations
 data <- merged_results

 #Vector stating the order you want the measures to be displayed
 measure_order <- c("H0b.Jac.AvLast", "H0b.Sor.AvLast",
 "D0b.A.AvLast", "D0b.B.AvLast",
 "H0b.Jac.AvFirst", "H0b.Sor.AvFirst",
 "D0b.A.AvFirst", "D0b.B.AvFirst",
 "H1b.MI.AvLast", "H1b.ShD.AvLast",
 "D1b.A.AvLast", "D1b.B.AvLast",
 "H1b.MI.AvFirst", "H1b.ShD.AvFirst",
 "D1b.A.AvFirst", "D1b.B.AvFirst",
 "H2b.GST.AvLast", "H2b.JOST.AvLast",
 'D2b.A.AvLast', 'D2b.B.AvLast',
 "H2b.GST.AvFirst", "H2b.JOST.AvFirst",
 'D2b.A.AvFirst', 'D2b.B.AvFirst',
 "BC.AvLast")

 #Filter data down to default variables
 data <- data %>%
 filter(Measure %in% measure_order,
 n.samples == 20,
 n.loci == 1000,
 n.pops == 10)

 #Make a column for false positives
 #When step is zero, all steps detected are false
 #Otherwise only those not at 0.5 are false
 data <- data %>%
 mutate(false_pos = ifelse(step == 0, Total.Steps, Incorrect.Steps),
 Correct.Steps = ifelse(step == 0, NA, Correct.Steps))

 #Split into individual allele proportion treatments
 data_0_1 <- data %>%
 filter(p.start == 0) %>%
 filter(p.end == 1) %>%
 group_by(Measure, step) %>%
 summarise(correct = round(sum(Correct.Steps)/(n()), digits = 1),
 false_pos = round(sum(false_pos)/(n()), digits = 1)) %>%
 pivot_longer(names_to = "step_type", cols = c(correct, false_pos)) %>%
 pivot_wider(names_from = step, values_from = value) %>%
 rename_with(.cols = -c(Measure, step_type), ~ paste(.x, "0_1", sep = "_"))

 data_0.1_0.9 <- data %>%
 filter(p.start == 0.1) %>%
 filter(p.end == 0.9) %>%
 group_by(Measure, step) %>%
 summarise(correct = round(sum(Correct.Steps)/(n()), digits = 1),
 false_pos = round(sum(false_pos)/(n()), digits = 1)) %>%
 pivot_longer(names_to = "step_type", cols = c(correct, false_pos)) %>%
 pivot_wider(names_from = step, values_from = value) %>%
 rename_with(.cols = -c(Measure, step_type), ~ paste(.x, "0.1_0.9", sep = "_"))


 data_0_0.5 <- data %>%
 filter(p.start == 0) %>%
 filter(p.end == 0.5)%>%
 group_by(Measure, step) %>%
 summarise(correct = round(sum(Correct.Steps)/(n()), digits = 1),
 false_pos = round(sum(false_pos)/(n()), digits = 1)) %>%
 pivot_longer(names_to = "step_type", cols = c(correct, false_pos)) %>%
 pivot_wider(names_from = step, values_from = value) %>%
 rename_with(.cols = -c(Measure, step_type), ~ paste(.x, "0_0.5", sep = "_"))


 data_0_0.2 <- data %>%
 filter(p.start == 0) %>%
 filter(p.end == 0.2) %>%
 group_by(Measure, step) %>%
 summarise(correct = round(sum(Correct.Steps)/(n()), digits = 1),
 false_pos = round(sum(false_pos)/(n()), digits = 1)) %>%
 pivot_longer(names_to = "step_type", cols = c(correct, false_pos)) %>%
 pivot_wider(names_from = step, values_from = value) %>%
 rename_with(.cols = -c(Measure, step_type), ~ paste(.x, "0_0.2", sep = "_"))


 data_0.3_0.5 <- data %>%
 filter(p.start == 0.3) %>%
 filter(p.end == 0.5) %>%
 group_by(Measure, step) %>%
 summarise(correct = round(sum(Correct.Steps)/(n()), digits = 1),
 false_pos = round(sum(false_pos)/(n()), digits = 1)) %>%
 pivot_longer(names_to = "step_type", cols = c(correct, false_pos)) %>%
 pivot_wider(names_from = step, values_from = value) %>%
 rename_with(.cols = -c(Measure, step_type), ~ paste(.x, "0.3_0.5", sep = "_"))


 #Put all the data back together
 data_merge <- data_0_1 %>%
 left_join(data_0.1_0.9, by = c("Measure", "step_type"))%>%
 left_join(data_0_0.5, by = c("Measure", "step_type"))%>%
 left_join(data_0_0.2, by = c('Measure', "step_type"))%>%
 left_join(data_0.3_0.5, by = c("Measure", "step_type")) %>%
 rename_with(.cols = -c(Measure, step_type), ~ paste("step", .x, sep = "_"))

 #Make table in correct order
 data_merge <- data_merge %>%
 mutate(Measure = factor(Measure, levels = measure_order)) %>%
 arrange(Measure)

 #Format NAs as "-"
 data_merge <- data_merge %>%
 mutate_all(~replace(., is.na(.), "-"))

 # Change Labels for T/F positives
 data_merge$step_type[data_merge$step_type == "correct"] <- "True Positives"
 data_merge$step_type[data_merge$step_type == "false_pos"] <- "False Positives"


 #T/F positive steps formatting
 data_merge[3:22] <- lapply(data_merge[3:22], function(x) {

 v <- 1:(length(x) + 4)

 v[c(T, F)] <- color_tile("transparent", "lightblue")(c(x[c(T, F)], 0, 100))

 v[c(F, T)] <- color_tile("transparent", "tomato")(c(x[c(F, T)], 0, 100))

 return(v[1:length(x)])
 })

 #Make blank measure rows for easier reading
 data_merge$Measure <-c("H0b Jac. (AvLast)", " ", "H0b Sor. (AvLast)"," ",
 "D0b.A (AvLast)"," ", "D0b.B (AvLast)"," ",
 "H0b Jac. (AvFirst)"," ", "H0b Sor. (AvFirst)"," ",
 "D0b.A (AvFirst)"," ", "D0b.B (AvFirst)"," ",
 "H1b MI (AvLast)"," ", "H1b ShD (AvLast)"," ",
 "D1b.A (AvLast)"," ", "D1b.B (AvLast)"," ",
 "H1b MI (AvFirst)"," ", "H1b ShD (AvFirst)"," ",
 "D1b.A (AvFirst)"," ", "D1b.B (AvFirst)"," ",
 "H2b GST (AvLast)"," ", "H2b Jost-D (AvLast)"," ",
 'D2b.A (AvLast)'," ", 'D2b.B (AvLast)'," ",
 "H2b GST (AvFirst)"," ", "H2b Jost-D (AvFirst)"," ",
 'D2b.A (AvFirst)'," ", 'D2b.B (AvFirst)'," ",
 "BC (AvLast)", " ")


 #create one big table
 results_table <- data_merge %>%
 ungroup %>%
 kbl(col.names = c("Measure", "Type of Step", "0", "1", "5", "50", "0", "1", "5", "50",
 "0", "1", "5", "50", "0", "1", "5", "50", "0", "1", "5", "50"),
 escape = FALSE) %>%
 kable_classic(full_width = F, html_font = "Cambria") %>%
 #Add stub head for each allele treatment
 add_header_above(c(" " = 2,
 "Maximal \n range: \n p = 0 - 1" = 4,
 "Maximal range \n without fixation: \n p = 0.1 - 0.9" = 4,
 "Halfmaximal \n range: \n p = 0 - 0.5" = 4,
 "Narrow range \n near fixation: \n p = 0 - 0.2" = 4,
 "Narrow range \n far from fixation: \n p = 0.3 - 0.5" = 4)
 ) %>%
 #Add conditional formatting
 pack_rows("q = 0 Measures", 1, 16)%>%
 pack_rows("q = 1 Measures", 17, 32) %>%
 pack_rows("q = 2 Measures", 33, 48)%>%
 pack_rows("Bray-Curtis", 49, 50)

 save_kable(results_table, file = "./Outputs/standard_treatment_table.html")

 return(results_table)
}

higher_lower_same.R

#Runs a t-test and checks if sample 2 is "greater", "less"
# or ~ "notdifferent" (not significantly different)
# than sample 1
higher_lower_same <- function(m1, m2, s1, s2, n1, n2) {

 se <- sqrt((s1^2/n1) + (s2^2/n2))

 t <- (m1 - m2)/se

 # welch-satterthwaite df
 df <- ((s1^2/n1 + s2^2/n2)^2) / ((s1^2/n1)^2/(n1-1) + (s2^2/n2)^2/(n2-1))

 p.value <- 2*pt(-abs(t), df)

 p.value <- ifelse(is.na(p.value), 0, p.value)

 mean.difference <- m1 - m2

 if (is.na(mean.difference)) return(NA)

 if (p.value > 0.05) return("notdifferent")

 if (mean.difference == 0) return("notdifferent")

 if (mean.difference > 0) return("greater")

 if (mean.difference < 0) return("less")

}

make_properties_summary_table.R

make_properties_summary_table <- function(merged_results) {

## Rows - Measures

## Columns - Properties of measures
 # True Positive Detection - Sum all true positives
 # False Positive Detection - Sum all false positives
 # True Negative Detection - Sum all true negatives
 # Effect of Allele Proportion Position
 # Effect of Allele proportion difference


 #Get full dataset of all simulations
 data <- merged_results

 #Vector stating the order you want the measures to be displayed
 measure_order <- c("H1b.MI.AvLast", "H1b.MI.AvFirst",
 "H2b.GST.AvLast", "H2b.GST.AvFirst",
 "D2b.A.AvFirst","BC.AvLast")

 #Filter down to the six best measures
 data <- data %>%
 filter(Measure %in% measure_order)

 #Make a column for false positives
 #When step is zero, all steps detected are false
 #Otherwise only those not at 0.5 are false
 data <- data %>%
 mutate(false_pos = ifelse(step == 0, Total.Steps, Incorrect.Steps),
 true_pos = ifelse(step == 0, NA, Correct.Steps),
 true_neg = ifelse(step == 0, 100 - Total.Steps, NA))


 data_filt <- data %>%
 filter(
 (p.start == 0 & p.end == 1) |
 (p.start == 0.1 & p.end == 0.9) |
 (p.start == 0 & p.end == 0.5) |
 (p.start == 0 & p.end == 0.2) |
 (p.start == 0.3 & p.end == 0.5)
 ) %>%
 group_by(Measure) %>%
 summarise(true_pos_mean = round(mean(true_pos, na.rm = T), digits = 1),
 false_pos_mean = round(mean(false_pos, na.rm = T), digits = 1),
 true_neg_mean = round(mean(true_neg, na.rm = T), digits = 1))


 data_filt <- data_filt %>%
 mutate(Measure = factor(Measure, levels = measure_order)) %>%
 arrange(Measure)


 #create basic table
 results_table <- data_filt %>%
 kbl(col.names = c("Measures", "True Positive Detection",
 "False Positive Detection",
 "True Negative Detection")) %>%
 kable_classic(full_width = F, html_font = "Cambria")

 save_kable(results_table, file = "./Outputs/properties_summary_table.html")

 return(results_table)
 }

measure_step_detection_sensitivity.R

Takes inputs of: step, n.samples, p.start, p.end, n.samples, n.loci, n.pops, n_reps

And tallies number of correct and incorrect steps detected for each diversity measure

If any input is a vector, runs a set of simulations for each combination

and checks for a step 1000 times for each sample size returns data and plot

To allow rerunning of later functions, this is skipped if “merged_results” already exists

@param step Intensity of step (default = 0). Can be vector of values.

@param p.start Starting allele proportion (default = 0). Can be vector of values.

@param p.end End allele proportion (default = 1). Can be vector of values.

@param n.samples Number of genomes sampled (default = 0). Can be vector of values.

@param n.loci Number of loci (default = 1000). Can be vector of values.

@param n.pops Number of localities (default = 10). Can be vector of values.

@param n_reps Number of times to repeat simulation (default = 10). Can be vector of values.

measure_step_detection_sensitivity <- function(step = 0, p.start = 0, p.end = 1,
 n.samples = 20, n.loci = 1000,
 n.pops = 10, n_reps = 10) {

 #Create a data frame of every possible combination of input variables
 input_combinations <- purrr::cross_df(list(step = step,
 n.samples = n.samples,
 p.start = p.start,
 p.end = p.end,
 n.loci = n.loci,
 n.pops = n.pops,
 n_reps = n_reps))


 # Count number of steps detected for each combinations (run n_reps times)
 # Create a table with the model inputs, number of model replicates
 # steps detected for each measure and correct steps detected for each measure
 replication_table_step <- future_pmap_dfr(input_combinations,
 count_step_detections) %>%
 add_column(input_combinations, .before = 1)

 return(replication_table_step)

}

merge_results.R

Merges the results from step_sensitivities_samples, step_sensitivities_loci, step_sensitivities_pops into a single table - merged_results

merge_results <- function(step_sensitivities_samples,
 step_sensitivities_loci,
 step_sensitivities_pops) {

 #Convert to long format
 data_samples_long <- step_sensitivities_samples %>%
 #Take columns with "correct" and make new columns: Measure, Correct.Steps
 pivot_longer(contains("_correct"),
 names_to = "Measure.Correct", values_to = "Correct.Steps") %>%
 #Take columns with "coefvar" and make new columns: Measure, coefvar
 pivot_longer(contains("_coefvar"),
 names_to = "Measure.coefvar", values_to = "coefvar") %>%
 #Take columns with "correct" and make new columns: Measure, Correct.Steps
 pivot_longer(-c(step:n_reps, Measure.Correct, Correct.Steps, Measure.coefvar, coefvar),
 names_to = "Measure.Total", values_to = "Total.Steps") %>%
 #Remove "_correct" from Measure.Correct column
 mutate(Measure.Correct = sub("_correct", "", Measure.Correct)) %>%
 #Remove "_coefvar" from Measure.coefvar column
 mutate(Measure.coefvar = sub("_coefvar", "", Measure.coefvar)) %>%
 #just keep rows with matching Measures
 filter(Measure.Correct == Measure.Total) %>%
 #just keep rows with matching Measures
 filter(Measure.Correct == Measure.coefvar) %>%
 #Rename measure column
 rename(Measure = Measure.Correct) %>%
 #Select specific columns for output
 select(c(Measure, step, p.start, p.end, n.samples,
 n.loci, n.pops, Total.Steps, Correct.Steps, coefvar))


 data_loci_long <- step_sensitivities_loci %>%
 #Take columns with "correct" and make new columns: Measure, Correct.Steps
 pivot_longer(contains("_correct"),
 names_to = "Measure.Correct", values_to = "Correct.Steps") %>%
 #Take columns with "coefvar" and make new columns: Measure, coefvar
 pivot_longer(contains("_coefvar"),
 names_to = "Measure.coefvar", values_to = "coefvar") %>%
 #Take columns with "correct" and make new columns: Measure, Correct.Steps
 pivot_longer(-c(step:n_reps, Measure.Correct, Correct.Steps, Measure.coefvar, coefvar),
 names_to = "Measure.Total", values_to = "Total.Steps") %>%
 #Remove "_correct" from Measure.Correct column
 mutate(Measure.Correct = sub("_correct", "", Measure.Correct)) %>%
 #Remove "_coefvar" from Measure.coefvar column
 mutate(Measure.coefvar = sub("_coefvar", "", Measure.coefvar)) %>%
 #just keep rows with matching Measures
 filter(Measure.Correct == Measure.Total) %>%
 #just keep rows with matching Measures
 filter(Measure.Correct == Measure.coefvar) %>%
 #Rename measure column
 rename(Measure = Measure.Correct) %>%
 #Select specific columns for output
 select(c(Measure, step, p.start, p.end, n.samples,
 n.loci, n.pops, Total.Steps, Correct.Steps, coefvar))

 data_pops_long <- step_sensitivities_pops %>%
 #Take columns with "correct" and make new columns: Measure, Correct.Steps
 pivot_longer(contains("_correct"),
 names_to = "Measure.Correct", values_to = "Correct.Steps") %>%
 #Take columns with "coefvar" and make new columns: Measure, coefvar
 pivot_longer(contains("_coefvar"),
 names_to = "Measure.coefvar", values_to = "coefvar") %>%
 #Take columns with "correct" and make new columns: Measure, Correct.Steps
 pivot_longer(-c(step:n_reps, Measure.Correct, Correct.Steps, Measure.coefvar, coefvar),
 names_to = "Measure.Total", values_to = "Total.Steps") %>%
 #Remove "_correct" from Measure.Correct column
 mutate(Measure.Correct = sub("_correct", "", Measure.Correct)) %>%
 #Remove "_coefvar" from Measure.coefvar column
 mutate(Measure.coefvar = sub("_coefvar", "", Measure.coefvar)) %>%
 #just keep rows with matching Measures
 filter(Measure.Correct == Measure.Total) %>%
 #just keep rows with matching Measures
 filter(Measure.Correct == Measure.coefvar) %>%
 #Rename measure column
 rename(Measure = Measure.Correct) %>%
 #Select specific columns for output
 select(c(Measure, step, p.start, p.end, n.samples,
 n.loci, n.pops, Total.Steps, Correct.Steps, coefvar))


 #Merge all data together
 data <- data_samples_long %>%
 full_join(data_loci_long) %>%
 full_join(data_pops_long) %>%
 distinct(across(Measure:n.pops), .keep_all = T)

 #Add in incorrect steps column
 merged_results <- data %>%
 mutate(Incorrect.Steps = Total.Steps - Correct.Steps)

 saveRDS(merged_results, "Outputs/merged_results")

 return(merged_results)
}

plot_alphas.R

plot_alphas <- function(one_locus_data, measures, colours,
 expected = T, errorbars = T, insert = F) {
 #select data
 data <- one_locus_data$data_summary

 #select variables to print at the end
 variables <- one_locus_data$variables

 #calculate n for standard error
 n.se <- variables$n.loci

 #create base plot
 p <- ggplot(data) +
 ylab("Alpha Diversity") +
 xlab("Distance (0 to 1)") +
 xlim(0, 1) +
 theme_classic()

 #Function for adding error bars to plots
 gg_errorbars <- function(measure, colour){

 # Calculate standard errors
 data <- data %>%
 mutate(measure_se = !!sym(paste0(measure, "_sd"))/sqrt(n.se),
 measure_mean = !!sym(paste0(measure, "_mean")))

 p <- p +
 geom_errorbar( data = data,
 aes(
 x = d,
 y = measure_mean,
 ymin = ifelse(measure_mean - measure_se < 0, 0,
 measure_mean - measure_se),
 ymax = measure_mean + measure_se
 ),
 colour = colour,
 width = .05,
 position = position_dodge(10))

 return(p)
 }


 for (i in seq(1, length(measures))) {

 p <- p +
 geom_point(aes(d, !!sym((paste0(measures[i], "_mean")))), colour = colours[i]) +
 geom_line(aes(d, !!sym((paste0(measures[i], "_mean")))), colour = colours[i])

 if(errorbars == T) p <- gg_errorbars(measures[i], colours[i])

 }

 #Toggle of an iset plot of the underlying allele proportions
 if(insert == T){

 inset.plot <- ggplot(data) +
 geom_point(aes(d, p_mean))+
 theme(
 panel.background = element_rect(fill = "transparent"), # bg of the panel
 plot.background = element_rect(fill = "transparent", color = NA), # bg of the plot
 panel.grid.major = element_blank(), # get rid of major grid
 panel.grid.minor = element_blank(), # get rid of minor grid
 legend.background = element_rect(fill = "transparent"), # get rid of legend bg
 legend.box.background = element_rect(fill = "transparent") # get rid of legend panel bg
 )

 p <- ggdraw(p) +
 draw_plot(inset.plot, x = 0.7, y = .7, width = .3, height = .3)

 }

 return(alpha_plot = p)
}

plot_betas.R

plot_betas <- function(one_locus_data, measures, colours,
 expected = T, errorbars = T, insert = T) {
 #select data
 data <- one_locus_data$data_summary

 #select variables to print at the end
 variables <- one_locus_data$variables

 #calculate n for standard error
 n.se <- variables$n.loci

 #create base plot
 p <- ggplot(data) +
 ylab("Adjacent Beta Diversity") +
 xlab("Distance (0 to 1)") +
 xlim(0, 1) +
 theme_classic()


 if("all.AvLast" %in% measures) {measures <- c(measures,
 "H0b.Jac.AvLast", "H0b.Sor.AvLast",
 "H1b.MI.AvLast", "H1b.ShD.AvLast",
 "H2b.JOST.AvLast", "H2b.GST.AvLast",
 "D0b.A.AvLast", "D0b.B.AvLast",
 "D1b.A.AvLast", "D1b.B.AvLast",
 "D2b.A.AvLast", "D2b.B.AvLast", "BC.AvLast")}

 if("all.H.AvLast" %in% measures) {measures <- c(measures,
 "H0b.Jac.AvLast", "H0b.Sor.AvLast",
 "H1b.MI.AvLast", "H1b.ShD.AvLast",
 "H2b.JOST.AvLast", "H2b.GST.AvLast")}

 if("all.D.AvLast" %in% measures) {measures <- c(measures,
 "D0b.A.AvLast", "D0b.B.AvLast",
 "D1b.A.AvLast", "D1b.B.AvLast",
 "D2b.A.AvLast", "D2b.B.AvLast")}

 if("all.AvFirst" %in% measures) {measures <- c(measures,
 "H0b.Jac.AvFirst", "H0b.Sor.AvFirst",
 "H1b.MI.AvFirst", "H1b.ShD.AvFirst",
 "H2b.JOST.AvFirst", "H2b.GST.AvFirst",
 "D0b.A.AvFirst", "D0b.B.AvFirst",
 "D1b.A.AvFirst", "D1b.B.AvFirst",
 "D2b.A.AvFirst", "D2b.B.AvFirst")}

 if("all.H.AvFirst" %in% measures) {measures <- c(measures,
 "H0b.Jac.AvFirst", "H0b.Sor.AvFirst",
 "H1b.MI.AvFirst", "H1b.ShD.AvFirst",
 "H2b.JOST.AvFirst", "H2b.GST.AvFirst")}

 if("all.D.AvFirst" %in% measures) {measures <- c(measures,
 "D0b.A.AvFirst", "D0b.B.AvFirst",
 "D1b.A.AvFirst", "D1b.B.AvFirst",
 "D2b.A.AvFirst", "D2b.B.AvFirst")}

 #Function for adding error bars to plots
 gg_errorbars <- function(measure, colour){

 D_adjust <- ifelse(startsWith(measures[i], "D") == T, -1, 0)


 # Calculate standard errors
 data <- data %>%
 mutate(measure_se = !!sym(paste0(measure, "_sd"))/sqrt(n.se),
 measure_mean = !!sym(paste0(measure, "_mean")))

 p <- p +
 geom_errorbar( data = data,
 aes(
 x = d + i_mean / 2,
 y = measure_mean + D_adjust,
 ymin = ifelse(measure_mean - measure_se + D_adjust < 0, 0,
 measure_mean - measure_se + D_adjust),
 ymax = measure_mean + measure_se +D_adjust
 ),
 colour = colour,
 width = .05,
 position = position_dodge(10))

 return(p)
 }


 for (i in seq(1, length(measures))) {

 D_adjust <- ifelse(startsWith(measures[i], "D") == T, -1, 0)

 p <- p +
 geom_point(aes(d + i_mean / 2, !!sym((paste0(measures[i], "_mean"))) + !!D_adjust), colour = colours[i]) +
 geom_line(aes(d + i_mean / 2, !!sym((paste0(measures[i], "_mean"))) + !!D_adjust), colour = colours[i])

 if(errorbars == T) p <- gg_errorbars(measures[i], colours[i])

 }

 #Toggle of an iset plot of the underlying allele proportions
 if(insert == T){

 inset.plot <- ggplot(data) +
 geom_point(aes(d, p_mean))+
 theme(
 panel.background = element_rect(fill = "transparent"), # bg of the panel
 plot.background = element_rect(fill = "transparent", color = NA), # bg of the plot
 panel.grid.major = element_blank(), # get rid of major grid
 panel.grid.minor = element_blank(), # get rid of minor grid
 legend.background = element_rect(fill = "transparent"), # get rid of legend bg
 legend.box.background = element_rect(fill = "transparent") # get rid of legend panel bg
 )

 p <- ggdraw(p) +
 draw_plot(inset.plot, x = 0.7, y = .7, width = .3, height = .3)

 }

 return(list(beta_plot = p, variables = variables))
 }

q_diversity_functions.R

# Functions to calculate q-profile diversity measures
# From a vector of allele proportions (p)
# Assuming biallelic loci (i.e. p + (1-p) = 1)
# Both Entropy (H) and Diversity (D)
# Both Alpha (within population) and Beta (between population) measures
# For Beta measures, H/D is calculated per locus,
# Gamma calculations should use Alpha methods when all populations are pooled

### Alpha Diversity Functions

# For alpha diversity, take minor allele proportion of (p), and value of q
# p can either be a single value, or a vector of values
# each p is calculated independently,
# as you are calculating the diversity of each LOCUS (not all loci together)
# p can only range from 0 (100% one type) to 1 (100% alternate type)
# Missing data (NA), returns NA
get.Hq.alpha <- function (p, q){

 #Return error if q is not 0, 1, or 2
 if (!(q == 0|q == 1|q == 2)) {
 stop("Invalid value of q. Must be one of: 0, 1, 2")
 }

 #Return error if p>1 or p<0
 if (any(p > 1, na.rm = T) | any(p < 0, na.rm = T) ) {
 stop("Invalid value(s) of p. Must be between 0 and 1")
 }

 if (q == 0) {
 entropy <- ifelse((p == 0) | (p == 1), 0, 1)
 }

 if (q == 1) {
 entropy <- ifelse((p == 0) | (p == 1), 0,
 -(p * log(p)) - ((1 - p) * log(1 - p)))
 }

 if (q == 2) {
 entropy <- 1 - p*p - (1-p)*(1-p)
 }

 return(entropy)
}

# Take entropy (H) and convert it to effective number diversity (D), for q = 0,1,2
# Works with a single H, or H as a vector of values
# Note: to get average values of D, you should average H THEN convert to D
H.to.D.alpha <- function(H, q){

 #Return error if q is not 0, 1, or 2
 if (!(q == 0|q == 1|q == 2)) {
 stop("Invalid value of q. Must be one of: 0, 1, 2")
 }

 if (q == 0) {
 div <- H + 1
 }

 if (q == 1) {
 div <- exp(H)
 }

 if (q == 2) {
 div <- 1/(1-H)
 }

 return(div)
}

# Takes a vector of p (including a vector of length 1)
# It will only return a *single* D value (mean of H values, THEN converted to D)
# If you want to get average D measures, you should average H measures first
# THEN convert to a D measure, NOT the other way round
# (see Jensen's inequality for why)
get.Dq.alpha <- function (p, q){

 div <- H.to.D.alpha(mean(get.Hq.alpha(p, q), na.rm = T), q)

 return(div)
}

### Beta calculations

# Accepts two vectors of allele proportions to be compared (p1 and p2)
# And the value of q you want to calculate (0, 1, or 2)
# per.locus = T (locus variant)
# Returns a vector of entropies
# per.locus = F (global variant)
# Returns only 1 value, not a vector
# Optional variants for:
# q0measure - "Jaccard"/"Sorenson"
# q1measure - "Mutual Information"/"Shannon Differentiation"
# q2measure - "Jost-D"/ "GST"
get.Hq.beta <- function (p1, p2, q, per.locus = T,
 q0measure = "Jaccard",
 q1measure = "Mutual Information",
 q2measure = "Jost-D"){

 if (!(q == 0|q == 1|q == 2)) stop("Invalid value of q. Must be one of: 0, 1, 2")

 p.av <- (p1 + p2)/2 #Average minor allele proportion

 #When calculating entropy for each locus
 if (per.locus == T) {

 #Mean alpha diversity of localities, per locus
 Hqa.mean <- (get.Hq.alpha(p1, q) + get.Hq.alpha(p2, q))/2

 Hqa.mean.plus1 <- (get.Hq.alpha(p1, q) + get.Hq.alpha(p2, q) + 2)/2

 #Gamma diversity of localities, per locus
 Hqgamma <- get.Hq.alpha(p.av, q)

 #Number of shared alleles, per locus
 shared <- ifelse(p1 %% 1 > 0 & p2 %% 1 > 0 ,2, #both alleles shared
 ifelse((p1 == 0 & p2 == 1)| (p1 == 1 & p2 == 0 ), 0, #no shared alleles
 1)) #else, one shared allele
 }

 #When calculating global variant of diversity
 #Average entropies for each locus before calculating beta
 if (per.locus == F) {

 #AVERAGE Mean alpha diversity of localities, across all loci
 Hqa.mean <- mean((get.Hq.alpha(p1, q) + get.Hq.alpha(p2, q))/2, na.rm = T)

 Hqa.mean.plus1 <- mean((get.Hq.alpha(p1, q) + get.Hq.alpha(p2, q) + 2)/2, na.rm = T)

 #AVERAGE Gamma diversity of localities, across all loci
 Hqgamma <- mean(get.Hq.alpha(p.av, q), na.rm = T)

 shared <- mean(ifelse(p1 %% 1 > 0 & p2 %% 1 > 0 , 2, #both alleles shared
 ifelse((p1 == 0 & p2 == 1)|(p1 == 1 & p2 == 0 ), 0, #no shared alleles
 1)), #else, one shared alleles
 na.rm = T)
 }

 if (q == 0) {
 #Jaccard
 if (q0measure == "Jaccard") entropy <- 1 - (shared/(Hqgamma + 1))
 #Sorenson
 if (q0measure == "Sorenson") entropy <- 1 - (shared/Hqa.mean.plus1)
 }

 if (q == 1) {
 #Mutual Information (I)
 if (q1measure == "Mutual Information") entropy <- (Hqgamma - Hqa.mean)
 #Shannon differentiation - I normalised to a [0,1] scale
 if (q1measure == "Shannon Differentiation") entropy <- (Hqgamma - Hqa.mean)/log(2)
 }

 if (q == 2) {
 #Jost-D
 if (q2measure == "Jost-D") entropy <- ((Hqgamma - Hqa.mean)/(1 - Hqa.mean)) * 2
 #Gst
 if (q2measure == "GST") entropy <- ifelse(Hqgamma == 0, 0, (Hqgamma - Hqa.mean)/Hqgamma)
 }

 return(entropy)
}

get.Hq.beta.sd <- function (p1, p2, q,
 q0measure = "Jaccard",
 q1measure = "Mutual Information",
 q2measure = "Jost-D"){

 if (!(q == 0|q == 1|q == 2)) stop("Invalid value of q. Must be one of: 0, 1, 2")

 p.av <- (p1 + p2)/2 #Pooled allele proportion

 #AVERAGE Mean alpha diversity of localities, across all loci
 A_mean <- mean((get.Hq.alpha(p1, q) + get.Hq.alpha(p2, q))/2, na.rm = T)
 #sd
 A_var <- var((get.Hq.alpha(p1, q) + get.Hq.alpha(p2, q))/2, na.rm = T)


 #AVERAGE Mean alpha diversity of localities, across all loci
 S_mean <- mean((get.Hq.alpha(p1, q) + get.Hq.alpha(p2, q))/2 + 1, na.rm = T)
 #sd
 S_var <- var((get.Hq.alpha(p1, q) + get.Hq.alpha(p2, q))/2 + 1, na.rm = T)

 #AVERAGE Gamma diversity of localities, across all loci
 G_mean <- mean(get.Hq.alpha(p.av, q), na.rm = T)
 #sd
 G_var <- var(get.Hq.alpha(p.av, q), na.rm = T)


 R_mean <- mean(ifelse(p1 %% 1 > 0 & p2 %% 1 > 0 , 2, #both alleles shared
 ifelse((p1 == 0 & p2 == 1)|(p1 == 1 & p2 == 0 ), 0, #no shared alleles
 1)), #else, one shared alleles
 na.rm = T)

 R_var <- var(ifelse(p1 %% 1 > 0 & p2 %% 1 > 0 , 2, #both alleles shared
 ifelse((p1 == 0 & p2 == 1)|(p1 == 1 & p2 == 0 ), 0, #no shared alleles
 1)), #else, one shared alleles
 na.rm = T)


 if (q == 0) {
 #Jaccard
 if (q0measure == "Jaccard") entropy_var <- R_var/((G_mean +1)^2 )+ ((R_mean^2)*G_var)/((G_mean +1)^4)
 #Sorenson
 if (q0measure == "Sorenson") entropy_var <- 4*R_var/(S_mean^2) + 4*((R_mean^2)*R_var)/(S_mean^4)
 }

 if (q == 1) {
 #Mutual Information (I)
 if (q1measure == "Mutual Information") entropy_var <- G_var + A_var
 #Shannon differentiation - I normalised to a [0,1] scale
 if (q1measure == "Shannon Differentiation") entropy_var <- (G_var + A_var) / (log(2)^2)
 }

 if (q == 2) {
 #Jost-D
 if (q2measure == "Jost-D") entropy_var <- 4 * (G_var/(A_mean +1)^2) + (4*(G_mean -1)^2 *A_var)/ ((A_mean +1)^4)
 #Gst
 if (q2measure == "GST") entropy_var <- (A_mean^2 * G_var)/(G_mean^4) + G_var/(G_mean^2)
 }

 return(sqrt(entropy_var)) #standard deviation
}

# Accepts two vectors of allele proportions to be compared
# per.locus = T
# Returns a vector of diversities
# per.locus = F
# Returns only 1 value, not a vector
get.Dq.beta <- function (p1, p2, q, per.locus = T){

 if (!(q == 0|q == 1|q == 2)) stop("Invalid value of q. Must be one of: 0, 1, 2")

 p.av <- (p1 + p2)/2 #Pooled allele proportion

 if (per.locus == T) {
 #Mean alpha diversity of localities, per loci
 #Then converted to D, per loci
 Dqa.mean <- H.to.D.alpha((get.Hq.alpha(p1, q) + (get.Hq.alpha(p2, q)))/2, q)

 #Gamma diversity of localities, per loci
 #Then converted to D
 Dqgamma <- H.to.D.alpha(get.Hq.alpha(p.av, q), q)
 }

 if (per.locus == F) {

 #AVERAGE Mean alpha diversity of localities, across all loci
 Hqa.mean <- mean((get.Hq.alpha(p1, q) + get.Hq.alpha(p2, q))/2, na.rm = T)

 #Then converted to D
 Dqa.mean <- H.to.D.alpha(Hqa.mean, q)

 #AVERAGE Gamma diversity of localities, across all loci
 #Then converted to D
 Dqgamma <- H.to.D.alpha(mean(get.Hq.alpha(p.av, q), na.rm = T), q)
 }

 #Calculate diversity (D) measure
 #works for vectors (per.locus) or single values (global)
 div <- Dqgamma/Dqa.mean

 return(div)
}

get.Dq.beta.sd <- function (p1, p2, q){

 if (!(q == 0|q == 1|q == 2)) stop("Invalid value of q. Must be one of: 0, 1, 2")

 p.av <- (p1 + p2)/2 #Pooled allele proportion

 #AVERAGE Mean alpha diversity of localities, across all loci
 A_mean <- mean(H.to.D.alpha((get.Hq.alpha(p1, q) + get.Hq.alpha(p2, q))/2, q), na.rm = T)

 A_var <- var(H.to.D.alpha((get.Hq.alpha(p1, q) + get.Hq.alpha(p2, q))/2, q), na.rm = T)


 #AVERAGE Gamma diversity of localities, across all loci
 #Then converted to D
 G_mean <- mean(H.to.D.alpha(get.Hq.alpha(p.av, q), q), na.rm = T)

 G_var <- var(H.to.D.alpha(get.Hq.alpha(p.av, q), q), na.rm = T)


 #Calculate diversity (D) measure
 #works for vectors (per.locus) or single values (global)
 div_var <- (G_var)/(A_mean^2) + (G_mean ^2 * A_var)/(A_mean^4)

 return(sqrt(div_var))
}

get.Hq.relative.beta <- function (p1, p2, q, per.locus = T,
 q0measure = "Jaccard",
 q1measure = "Mutual Information",
 q2measure = "Jost-D"){

 if (!(q == 0|q == 1|q == 2)) stop("Invalid value of q. Must be one of: 0, 1, 2")

 p.av <- (p1 + p2)/2 #Pooled allele proportion

 #When calculating entropy for each locus
 if (per.locus == T) {

 #Mean alpha diversity of localities, per locus
 Hqa.mean <- (get.Hq.alpha(p1, q) + get.Hq.alpha(p2, q))/2

 Hqa.mean.plus1 <- (get.Hq.alpha(p1, q) + get.Hq.alpha(p2, q) + 2)/2

 #Gamma diversity of localities, per locus
 Hqgamma <- get.Hq.alpha(p.av, q)

 #Number of shared alleles, per locus
 shared <- ifelse(p1 %% 1 > 0 & p2 %% 1 > 0 , 2, #both alleles shared
 ifelse((p1 == 0 & p2 == 1)|(p1 == 1 & p2 == 0 ), 0, #no shared alleles
 1)) #else, one shared allele
 }

 #When calculating global entropy
 #Average entropies for each locus before calculating beta
 if (per.locus == F) {

 #AVERAGE Mean alpha diversity of localities, across all loci
 Hqa.mean <- mean((get.Hq.alpha(p1, q) + get.Hq.alpha(p2, q))/2, na.rm = T)

 Hqa.mean.plus1 <- mean((get.Hq.alpha(p1, q) + get.Hq.alpha(p2, q) + 2)/2, na.rm = T)

 #AVERAGE Gamma diversity of localities, across all loci
 Hqgamma <- mean(get.Hq.alpha(p.av, q), na.rm = T)

 shared <- mean(ifelse(p1 %% 1 > 0 & p2 %% 1 > 0 , 2, #both alleles shared
 ifelse((p1 == 0 & p2 == 1)|(p1 == 1 & p2 == 0 ), 0, #no shared alleles
 1)), #else, one shared alleles
 na.rm = T)
 }

 if (q == 0) {
 #Jaccard
 if (q0measure == "Jaccard") entropy <- 1 - (shared/(Hqgamma + 1))
 #Sorenson
 if (q0measure == "Sorenson") entropy <- 1 - (shared/Hqa.mean.plus1)
 }

 if (q == 1) {
 #Mutual Information (I)
 if (q1measure == "Mutual Information") entropy <- (Hqgamma - Hqa.mean)
 #Shannon differentiation - I normalised to a [0,1] scale
 if (q1measure == "Shannon Differentiation") entropy <- (Hqgamma - Hqa.mean)/log(2)
 }

 if (q == 2) {
 #Jost-D
 if (q2measure == "Jost-D") entropy <- ((Hqgamma - Hqa.mean)/(1 - Hqa.mean)) * 2
 #Gst
 if (q2measure == "GST") entropy <- ifelse(Hqgamma == 0, 0, (Hqgamma - Hqa.mean)/Hqgamma)
 }

 return(entropy/Hqa.mean)
}


get.Dq.relative.beta <- function (p1, p2, q, per.locus = T){

 if (!(q == 0|q == 1|q == 2)) stop("Invalid value of q. Must be one of: 0, 1, 2")

 p.av <- (p1 + p2)/2 #Pooled allele proportion

 if (per.locus == T) {
 #Mean alpha diversity of localities, per loci
 #Then converted to D, per loci
 Dqa.mean <- H.to.D.alpha((get.Hq.alpha(p1, q) + (get.Hq.alpha(p2, q)))/2, q)

 #Gamma diversity of localities, per loci
 #Then converted to D
 Dqgamma <- H.to.D.alpha(get.Hq.alpha(p.av, q), q)
 }

 if (per.locus == F) {

 #AVERAGE Mean alpha diversity of localities, across all loci
 Hqa.mean <- mean((get.Hq.alpha(p1, q) + get.Hq.alpha(p2, q))/2, na.rm = T)

 #Then converted to D
 Dqa.mean <- H.to.D.alpha(Hqa.mean, q)

 #AVERAGE Gamma diversity of localities, across all loci
 #Then converted to D
 Dqgamma <- H.to.D.alpha(mean(get.Hq.alpha(p.av, q), na.rm = T), q)
 }

 #Calculate diversity (D) measure
 #works for vectors (per.locus) or single values (global)
 div <- Dqgamma/(Dqa.mean^2)

 return(div)
}


# Accepts two vectors of allele proportions to be compared
# Returns a vector of Bray-Curtis
get.BC <- function (p1, p2) abs(p1-p2)

#Calculate relative Bray-Curtis
get.RBC <- function (p1, p2) abs((p1-p2)/((p1+p2)/2))

read_specific_plot.R

Get a plot of beta diversities from one_locus_data

read_specific_plot <- function(step, n.samples, p.start, p.end, n.loci,
 n.pops, ...) {


plot_index <- detect_index(one_locus_data[c(T,F,F)], ~
 ((.x$step == step) & #options: 0, 1, 5, 10, 50
 (.x$n.samples == n.samples) & #options: 5, 10, 25
 (.x$p.start == p.start) & #options: 0, 0.1, 0.3
 (.x$p.end == p.end) & #options: 0.4, 0.5, 0.9, 1
 (.x$n.loci == n.loci) & #options: 10, 50, 1000
 (.x$n.pops == n.pops))) #options: 4, 5, 6, 7, 10, 11


beta_plot <- get.one.locus.plot(readd(one_locus_data, subtargets = plot_index), ...)$beta_plot

return(beta_plot)
}


#read_specific_plot(step = 0, n.samples = 10, p.start = 0.3,
 # p.end = 0.4, n.loci = 1000, n.pops = 11,
 # div_type = "D", expected = T, errorbars = T, ratios = F)

simulate_data.R

Simulate genetic data and check for steps

@title simulate_data @param step strength of step: 0 (linear) to x (steep step e.g. 50)

@param p.start starting allele proportion, 0 to 1

@param p.end end allele proportion, 0 to 1

@param n.samples number of genomes sampled from a locality

@param n.loci number of loci per genome

@param n.pops number of localities sampled along gradient

@param detect_step T/F for detecting steps

Calculated variables d = distance from 0 to 1, increments of increment.size - 1/(n.pops - 1)

@return output variables table of inputted variables

@return output data_summary table of simulated data

@return output$step_results table of step detection results

simulate_data <- function(step = 0, p.start = 0, p.end = 1, n.samples = 20,
 n.loci = 1000, n.pops = 10, detect_step = T) {

 #Make a table of the variables used for this run
 data_variables <- tibble(step, n.samples, p.start, p.end, n.loci, n.pops)

 #Add variables to output
 output <- list(variables = data_variables)

 #Make a variable which is the distance between each value of d (distance)
 increment.size = 1/(n.pops - 1)

 #Create a table with input variables and calculate p along distance (d)
 data <- tibble(
 step = step,
 n.samples = n.samples,
 i = increment.size,
 n.pops = n.pops,
 p.start = p.start,
 p.end = p.end,
 d = seq(0, 1, increment.size)) %>% #Location of population along distance (0 to 1)
 mutate(p = qbeta(d, 1 / (1 + step), 1 / (1 + step)) * #true population allele frequency
 (p.end - p.start) + p.start) #offsets allele proportion from 0 and 1

 ## Take random samples from p to get variable allele frequencies
 # Take n.samples from a binomial distribution around allele proportion p
 # Divide by n.samples to get 'measured' allele frequency
 # e.g. n.samples = 5, allele proportion p = 0.1
 # rbinom(1, 5, 0.1) / 5
 # Can only be 0, 0.2, 0.4, 0.6, 0.8, 1
 # But would more likely be 0/0.2
 # repeat over n.loci
 data <- data %>%
 .[rep(1:nrow(.), times = n.loci),] %>% #replicate over multiple loci with same p
 rowwise() %>% #allows for mutate to work row by row (rather than as a vector)
 mutate(p.binom = rbinom(1, n.samples, p)/n.samples) %>% #p.binom
 ungroup() #stops rowwise operations


 ## Alpha diversities

 #Calculate alpha diversities of each p.binom (i.e. sampled allele frequency)
 #Calculated at the locus level
 data <- data %>%
 mutate(
 H0a = get.Hq.alpha(p.binom, 0),
 H1a = get.Hq.alpha(p.binom, 1),
 H2a = get.Hq.alpha(p.binom, 2),
 D0a = H.to.D.alpha(H0a, 0),
 D1a = H.to.D.alpha(H1a, 1),
 D2a = H.to.D.alpha(H2a, 2)
 )

 ## Beta diversities - per locus - AvLast variants

 # Calculate beta diversities of each p.binom (i.e. sampled allele frequencies)
 # with the lead p.binom (distance + increment.size)
 #
 # Calculated at the locus level
 #
 # Don't calculate betas comparing distance 1 and 0 (d = 1), return NA instead
 data <- data %>%
 mutate(p.lead = lead(p.binom)) %>% #Add a column containing next p.binom
 mutate(
 H0b.Jac.AvLast = if_else(d == 1, NA_real_, get.Hq.beta(p.binom, p.lead, 0, q0measure = "Jaccard")),
 H0b.Sor.AvLast = if_else(d == 1, NA_real_, get.Hq.beta(p.binom, p.lead, 0, q0measure = "Sorenson")),
 H1b.MI.AvLast = if_else(d == 1, NA_real_, get.Hq.beta(p.binom, p.lead, 1, q1measure = "Mutual Information")),
 H1b.ShD.AvLast = if_else(d == 1, NA_real_, get.Hq.beta(p.binom, p.lead, 1, q1measure = "Shannon Differentiation")),
 H2b.JOST.AvLast = if_else(d == 1, NA_real_, get.Hq.beta(p.binom, p.lead, 2, q2measure = "Jost-D")),
 H2b.GST.AvLast = if_else(d == 1, NA_real_, get.Hq.beta(p.binom, p.lead, 2, q2measure = "GST")),
 D0b.A.AvLast = if_else(d == 1, NA_real_, get.Dq.beta(p.binom, p.lead, 0)),
 D0b.B.AvLast = H.to.D.alpha(H0b.Jac.AvLast, 0),
 D1b.A.AvLast = if_else(d == 1, NA_real_, get.Dq.beta(p.binom, p.lead, 1)),
 D1b.B.AvLast = H.to.D.alpha(H1b.MI.AvLast, 1),
 D2b.A.AvLast = if_else(d == 1, NA_real_, get.Dq.beta(p.binom, p.lead, 2)),
 D2b.B.AvLast = H.to.D.alpha(H2b.JOST.AvLast, 2),
 BC.AvLast = if_else(d == 1, NA_real_, get.BC(p.binom, p.lead)),
 RBC.AvLast = if_else(d == 1, NA_real_, get.RBC(p.binom, p.lead)),
 H0b.Jac.rel.AvLast = if_else(d == 1, NA_real_, get.Hq.relative.beta(p.binom, p.lead, 0, q0measure = "Jaccard")),
 H0b.Sor.rel.AvLast = if_else(d == 1, NA_real_, get.Hq.relative.beta(p.binom, p.lead, 0, q0measure = "Sorenson")),
 H1b.MI.rel.AvLast = if_else(d == 1, NA_real_, get.Hq.relative.beta(p.binom, p.lead, 1, q1measure = "Mutual Information")),
 H1b.ShD.rel.AvLast = if_else(d == 1, NA_real_, get.Hq.relative.beta(p.binom, p.lead, 1, q1measure = "Shannon Differentiation")),
 H2b.JOST.rel.AvLast = if_else(d == 1, NA_real_, get.Hq.relative.beta(p.binom, p.lead, 2, q2measure = "Jost-D")),
 H2b.GST.rel.AvLast = if_else(d == 1, NA_real_, get.Hq.relative.beta(p.binom, p.lead, 2, q2measure = "GST")),
 D0b.A.rel.AvLast = if_else(d == 1, NA_real_, get.Dq.relative.beta(p.binom, p.lead, 0)),
 D0b.B.rel.AvLast = H.to.D.alpha(H0b.Jac.rel.AvLast, 0),
 D1b.A.rel.AvLast = if_else(d == 1, NA_real_, get.Dq.relative.beta(p.binom, p.lead, 1)),
 D1b.B.rel.AvLast = H.to.D.alpha(H1b.MI.rel.AvLast, 1),
 D2b.A.rel.AvLast = if_else(d == 1, NA_real_, get.Dq.relative.beta(p.binom, p.lead, 2)),
 D2b.B.rel.AvLast = H.to.D.alpha(H2b.JOST.rel.AvLast, 2),
 )

 ## Beta diversities - AvFirst (average gamma, alphas before calculating beta)

 # Calculate beta diversities of each p.binom (i.e. sampled allele frequencies)
 # with the lead p.binom (distance + increment.size)
 #
 # Calculated overall per group (each site)
 #
 #Don't calculate betas comparing distance 1 and 0 (d =1), return NA instead
 AvFirst_data <- data %>%
 group_by(d) %>%
 filter(d != 1) %>% #Avoid calculating at d = 1
 mutate(
 H0b.Jac.AvFirst_mean = get.Hq.beta(p.binom, p.lead, 0, per.locus = F, q0measure = "Jaccard"),
 H0b.Sor.AvFirst_mean = get.Hq.beta(p.binom, p.lead, 0, per.locus = F, q0measure = "Sorenson"),
 H1b.MI.AvFirst_mean = get.Hq.beta(p.binom, p.lead, 1, per.locus = F, q1measure = "Mutual Information"),
 H1b.ShD.AvFirst_mean = get.Hq.beta(p.binom, p.lead, 1, per.locus = F, q1measure = "Shannon Differentiation"),
 H2b.JOST.AvFirst_mean = get.Hq.beta(p.binom, p.lead, 2, per.locus = F, q2measure = "Jost-D"),
 H2b.GST.AvFirst_mean = get.Hq.beta(p.binom, p.lead, 2, per.locus = F, q2measure = "GST"),
 D0b.A.AvFirst_mean = get.Dq.beta(p.binom, p.lead, 0, per.locus = F),
 D0b.B.AvFirst_mean = H.to.D.alpha(H0b.Jac.AvFirst_mean, 0),
 D1b.A.AvFirst_mean = get.Dq.beta(p.binom, p.lead, 1, per.locus = F),
 D1b.B.AvFirst_mean = H.to.D.alpha(H1b.MI.AvFirst_mean, 1),
 D2b.A.AvFirst_mean = get.Dq.beta(p.binom, p.lead, 2, per.locus = F),
 D2b.B.AvFirst_mean = H.to.D.alpha(H2b.JOST.AvFirst_mean, 2)
 ) %>%
 mutate(
 H0b.Jac.AvFirst_sd = get.Hq.beta.sd(p.binom, p.lead, 0, q0measure = "Jaccard"),
 H0b.Sor.AvFirst_sd = get.Hq.beta.sd(p.binom, p.lead, 0, q0measure = "Sorenson"),
 H1b.MI.AvFirst_sd = get.Hq.beta.sd(p.binom, p.lead, 1, q1measure = "Mutual Information"),
 H1b.ShD.AvFirst_sd = get.Hq.beta.sd(p.binom, p.lead, 1, q1measure = "Shannon Differentiation"),
 H2b.JOST.AvFirst_sd = get.Hq.beta.sd(p.binom, p.lead, 2, q2measure = "Jost-D"),
 H2b.GST.AvFirst_sd = get.Hq.beta.sd(p.binom, p.lead, 2, q2measure = "GST"),
 D0b.A.AvFirst_sd = get.Dq.beta.sd(p.binom, p.lead, 0),
 D0b.B.AvFirst_sd = H0b.Jac.AvFirst_sd, #same sd as H
 D1b.A.AvFirst_sd = get.Dq.beta.sd(p.binom, p.lead, 1),
 D1b.B.AvFirst_sd = H1b.MI.AvFirst_sd, #same sd as H
 D2b.A.AvFirst_sd = get.Dq.beta.sd(p.binom, p.lead, 2),
 D2b.B.AvFirst_sd = H2b.JOST.AvFirst_sd #same sd as H
 ) %>%
 summarise(across( everything(), mean)) %>%
 select("d", ends_with("_mean"), ends_with("_sd"))


 #Create data summary table,
 #what are the mean and sd of each measure at each distance (d)
 data_summary <- data %>%
 group_by(d) %>%
 summarise_each(list(mean = mean, sd = sd, var = var)) %>%
 left_join(AvFirst_data, by = "d")

 #Add data summary to output
 output <- c(output, list(data_summary = data_summary))

 #Check for presence and location of step (optional)
 if(detect_step == T){

 #Names of each beta measure
 beta_measure_names <- c("H0b.Jac", "H0b.Sor", "H1b.MI", "H1b.ShD",
 "H2b.JOST", "H2b.GST", "D0b.A", "D0b.B",
 "D1b.A", "D1b.B", "D2b.A", "D2b.B")

 #Names of each beta measure including their by AvLast and AvFirst variant
 beta_measures <- c(paste0(beta_measure_names, ".AvLast"),
 paste0(beta_measure_names, ".rel.AvLast"),
 paste0(beta_measure_names, ".AvFirst"),
 "BC.AvLast", "RBC.AvLast")

 #Detect for a step for each measure, also calculate coefficient of variation
 step_results <- map_dfr(beta_measures, step_check, data = data_summary, n.loci)

 #Add step results to output
 output <- c(output, list(step_results = step_results))
 }

return(output)
}

step_check.R

Checks for step in data by finding beta values at point: d being significantly lower than d + 1 AND d + 1 being significantly higher than d + 2

OR

d being significantly lower than d + 1 AND d + 1 being not different to d + 2 AND d + 2 being significantly higher than d + 3

@param measure Diversity measure to check for step

@param data one_locus_data

@param n.loci Number of loci

@return A tibble containing: measure = name of measure step_present = Is the step present (T/F) step_location_start = location of start of step range step_location_end = location of end of step range

@title Step check

step_check <- function(measure, data, n.loci) {

 #Column containing mean beta values for the measure being tested
 mean_vec <- paste0(measure, "_mean")

 #Column containing sd beta values for the measure being tested
 sd_vec <- paste0(measure, "_sd")

 #Run higher_lower_same function (t.test) over each beta diversity value
 beta_diff <- pmap_chr(list(m1 = data[[mean_vec]], m2 = lead(data[[mean_vec]]),
 s1 = data[[sd_vec]], s2 = lead(data[[sd_vec]])),
 higher_lower_same,
 n1 = n.loci, n2 = n.loci)

 x <- data[c("d", mean_vec, sd_vec)] %>% #select just d, mean, sd of measure
 cbind(beta_diff) %>% #add higher_lower_same results
 mutate(step_location_start = ifelse((lag(beta_diff) == "less" & #Is there a step at this d
 beta_diff == "greater"),
 d, #Step between d and d + 1
 ifelse(lag(beta_diff) == "less" & #Is there a step between this d
 beta_diff == "notdifferent" & # and the next d
 lead(beta_diff) == "greater",
 d, #Step between d and d +2
 NA)), #else NA
 step_location_end = ifelse((lag(beta_diff) == "less" & #Is there a step at this d
 beta_diff == "greater"),
 lead(d), #Step between d and d + 1
 ifelse(lag(beta_diff) == "less" & #Is there a step between this d
 beta_diff == "notdifferent" & # and the next d
 lead(beta_diff) == "greater",
 lead(d, 2), #Step between d and d +2
 NA)))

 #Replace any Inf/ -Inf values with NA
 step_location_start <- ifelse(!is.null((na.omit(x$step_location_start))),
 (na.omit(x$step_location_start)), NA)

 #Replace any Inf/ -Inf values with NA
 step_location_end <- ifelse(!is.null((na.omit(x$step_location_end))),
 (na.omit(x$step_location_end)), NA)

 #T/F column indictating if a step is present
 step_present <- ifelse(!is.na(step_location_start), T, F)

 return(tibble(measure = measure,
 step_present = step_present,
 step_location_start = step_location_start,
 step_location_end = step_location_end
 ))
}

vis_alpha_peaks.R

vis_alpha_peaks <- function() {

 alpha_peaks <- plot_grid(plot_alphas(simulate_data(step =0,),"H0a", "red"),
 plot_alphas(simulate_data(step =50),"H0a", "red"),
 plot_alphas(simulate_data(step =0),"H1a", "blue"),
 plot_alphas(simulate_data(step =50),"H1a", "blue"),
 plot_alphas(simulate_data(step =0),"H2a", "darkgreen"),
 plot_alphas(simulate_data(step =50),"H2a", "darkgreen"),
 nrow = 3,
 labels = c("A", "B", "C", "D", "E", "F"))

 return(alpha_peaks)

 }

visualise_beta_trends.R

visualise_beta_trends <- function() {

 #How do each of the measures vary from 0 to 1
 #i.e. location 1 has a allele frequency of (p = 0)
 # and location 2 has a allele frequency of (p = 0 to 1)
 #So differentiation should range from 0 (no differentiation)
 # to 1 (complete differentiation)

 p.start = 0.1
 p.end = 0.9
 n.samples = 10
 n.loci = 1000
 #Make a variable which is the distance between each value of d (distance)
 increment.size = 0.01


 #Create a table with
 data <- tibble(
 n.samples = n.samples,
 i = increment.size,
 p.start = p.start,
 p.end = p.end,
 d = seq(0, 1, increment.size)) %>% #Location of population along distance (0 to 1)
 mutate(p = p.start + d*(p.end - p.start))

 #p.binom:
 #Take n.samples from a binomial distribution around allele proportion p
 #Divide by n.samples to get 'measured' allele frequency
 #e.g. n.samples = 5, allele proportion p = 0.1
 # rbinom(1,5,0.1) / 5
 # Can only be 0, 0.2, 0.4, 0.6, 0.8, 1
 # But would more likely be 0/0.2
 # repeat over n.loci
 data <- data %>%
 .[rep(1:nrow(.), times = n.loci),] %>% #replicate over multiple loci with same p
 rowwise() %>% #allows for mutate to work row by row (rather than as a vector)
 mutate(p.binom = rbinom(1, n.samples, p)/n.samples) %>% #p.binom
 ungroup() #stops rowwise operations

 data <- data %>%

 mutate(H0b = get.Hq.beta.per.locus(p.start, p.binom, 0),
 H1b = get.Hq.beta.per.locus(p.start, p.binom, 1),
 H2b.JOST = get.Hq.beta.per.locus(p.start, p.binom, 2),
 H2b.GST = get.Hq.beta.per.locus(p.start, p.binom, 2, "Gst"),
 D0b = get.Dq.beta.per.locus(p.start, p.binom, 0),
 D1b = get.Dq.beta.per.locus(p.start, p.binom, 1),
 D2b = get.Dq.beta.per.locus(p.start, p.binom, 2),
 AFD = get.AFD(p.start, p.binom)
 )

 data_summary <- data %>%
 group_by(d) %>%
 summarise_each(list(mean = mean, sd = sd)) %>%
 as_tibble()%>%
 mutate(H0b = get.Hq.beta.per.locus(p.start_mean, p_mean, 0),
 H1b = get.Hq.beta.per.locus(p.start_mean, p_mean, 1),
 H2b.JOST = get.Hq.beta.per.locus(p.start_mean, p_mean, 2),
 H2b.GST = get.Hq.beta.per.locus(p.start_mean, p_mean, 2, "Gst"),
 D0b = get.Dq.beta.per.locus(p.start_mean, p_mean, 0),
 D1b = get.Dq.beta.per.locus(p.start_mean, p_mean, 1),
 D2b = get.Dq.beta.per.locus(p.start_mean, p_mean, 2),
 AFD = get.AFD(p.start_mean, p_mean)
 )

 ggplot(data_summary, aes(x = d))+
 geom_point(shape = 3, aes(y = H2b.GST_mean), colour = "darkgreen")+
 geom_point(shape = 3, aes(y = H1b_mean), colour = "lightblue")+
 geom_point(shape = 3, aes(y = H0b_mean), colour = "red")+
 geom_point(shape = 3, aes(y = H2b.JOST_mean), colour = "purple")+
 geom_point(shape = 3, aes(y = AFD_mean), colour = "orange")+
 geom_point(shape = 3, aes(y = D1b_mean-1), colour = "blue")+
 geom_point(aes(y = D1b-1), colour = "blue")+
 geom_point(aes(y = H2b.GST), colour = "darkgreen")+
 geom_point(aes(y = H1b), colour = "lightblue")+
 geom_point(aes(y = H0b), colour = "red")+
 geom_point(aes(y = H2b.JOST), colour = "purple")+
 geom_point(aes(y = AFD), colour = "orange")

 ggplot(data_summary)+
 geom_point(aes(x = H2b.GST, y = H2b.GST_mean), colour = "darkgreen")+
 geom_point(aes(x = D1b-1, y = D1b_mean-1), colour = "green")+
 geom_point(aes(x = D1b-1, y = D1b_mean-1), colour = "blue")+
 geom_point(aes(x = H0b, y = H0b_mean), colour = "pink")+
 geom_point(aes(x = D0b-1, y = D0b_mean-1), colour = "red")+
 geom_point(aes(x = H1b, y = H1b_mean), colour = "lightblue")+
 geom_point(aes(x = H2b.JOST, y = H2b.JOST_mean), colour = "purple")+
 geom_point(aes(x = AFD, y = AFD_mean), colour = "orange")+
 geom_abline(slope=1)+
 xlab("True differentiation (0 to 1)")+
 ylab("Measured differentiation (0 to 1)")


}

visualise_step_detections.R

#variable - one of: n.samples, n.loci, n.pops

visualise_step_sensitivities <- function(measure, variable, colour) {

 if (variable == "n.samples") data <- readd(step_sensitivities_samples)

 if (variable == "n.loci") data <- readd(step_sensitivities_loci)

 if (variable == "n.pops") data <- readd(step_sensitivities_pops)

 #Creates 4 plots for each value of step (0, 1, 5, 50)
 # as a horizontal grid
 #Takes inputs:
 # measure - genetic diversity measure results to extract
 # colour - colour of lines to be plotted
 vis.beta.step.grid <- function(variable, p.start, p.end){

 #Base properties of each plot
 p <- ggplot()+
 ylab("Step detections (out of 100)") +
 ylim(0,100) +
 theme_classic()

 #step == 0
 p1 <- p +
 geom_line(data = filter(data, step == 0 & p.start == !!p.start & p.end == !!p.end),
 aes(x= !!sym(variable), y = !!sym(measure)), colour = colour, linetype = "dashed")

 #step == 1
 p2 <- p +
 geom_line(data = filter(data, step == 1 & p.start == !!p.start & p.end == !!p.end),
 aes(x= !!sym(variable), y = !!sym(measure)), colour = colour, linetype = "dashed") +
 geom_line(data = filter(data, step == 1 & p.start == !!p.start & p.end == !!p.end),
 aes(x = !!sym(variable), y = !!sym(paste0(measure, "_correct"))), colour = colour)

 #step == 5
 p3 <- p +
 geom_line(data = filter(data, step == 5 & p.start == !!p.start & p.end == !!p.end),
 aes(x= !!sym(variable), y = !!sym(measure)), colour = colour, linetype = "dashed") +
 geom_line(data = filter(data, step == 5 & p.start == !!p.start & p.end == !!p.end),
 aes(x = !!sym(variable), y = !!sym(paste0(measure, "_correct"))), colour = colour)

 #step == 50
 p4 <- p +
 geom_line(data = filter(data, step == 50 & p.start == !!p.start & p.end == !!p.end),
 aes(x= !!sym(variable), y = !!sym(measure)), colour = colour, linetype = "dashed") +
 geom_line(data = filter(data, step == 50 & p.start == !!p.start & p.end == !!p.end),
 aes(x = !!sym(variable), y = !!sym(paste0(measure, "_correct"))), colour = colour)

 #Merge the four plots together
 p_grid <- plot_grid(p1, p2, p3, p4, ncol = 4,
 labels = c('Step = 0', 'Step = 1', 'Step = 5', 'Step = 50'),
 label_size = 12, hjust = -1, vjust= 5)


 return(p_grid)
 }


 final_plot <- plot_grid(
 vis.beta.step.grid(variable, 0, 1),
 vis.beta.step.grid(variable, 0.1, 0.9),
 vis.beta.step.grid(variable, 0, 0.5),
 vis.beta.step.grid(variable, 0, 0.2),
 vis.beta.step.grid(variable, 0.3, 0.5),
 ncol = 1,
 labels = c("0 to 1", "0.1 to 0.9", "0 to 0.5", "0 to 0.2", "0.3 to 0.5"))


 ggsave(final_plot,
 filename = paste0("./Outputs/", measure, "_", variable, ".pdf"),
 height = 297, width = 210, unit = "mm")

}

create_measure_pdfs <- function() {

 beta_measure_names <- c("H0b.Jac", "H0b.Sor", "H1b.MI", "H1b.ShD",
 "H2b.JOST", "H2b.GST", "D0b.A", "D0b.B",
 "D1b.A", "D1b.B", "D2b.A", "D2b.B")

 #Names of each beta measure including their by locus and global variant
 beta_measures <- c(paste0(beta_measure_names, ".locus"),
 paste0(beta_measure_names, ".rel.locus"),
 paste0(beta_measure_names, ".global"),
 "BC.locus", "RBC.locus")

 test_variables <- c("n.samples", "n.loci", "n.pops")

 sens_vars <- cross_df(list(beta_measures = beta_measures,
 test_variables = test_variables))

 map2(sens_vars$beta_measures, sens_vars$test_variables,
 visualise_step_sensitivities, "black")

}
